# Amazon carbon emissions driven by human actions amplified by droughts

**DOI:** 10.1038/s41586-026-11168-1

**Published:** 2026-10-21

**Authors:** Luciana V. Gatti, Camilla L. Cunha, Juan C. G. Blas, A. Scott Denning, Mariane S. Reis, Luciano Marani, Egidio Arai, Cassiano Gustavo Messias, Dhemerson E. Conciani, John B. Miller, Vanúcia Schumacher, Kleber P. Naccarato, Jose Marengo, Ane A. C. Alencar, Luciana Soler, Claudio Almeida, Lucas Gatti Domingues, Caio S. C. Correia, Raiane A. L. Neves, Carlos Nobre, Sergio M. Corrêa, Elliott Campbell, Jennifer E. Johnson, Joseph A. Berry, Alber H. Sanchez, Francine M. da Silva, Vagner L. Camilotti, Christiane Silvestrini, Filipe Loyola Lopes, Xin Lan, Gabrielle Pétron, Marcio Malacarne, Guilherme B. M. Machado

**Affiliations:** 1 General Coordination of Earth Science (CGCT), National Institute for Space Research (INPE), São Paulo, Brazil; 2 Nuclear and Energy Research Institute (IPEN), São Paulo, Brazil; 3 Yale University, New Haven, CT USA; 4 Amazon Environmental Research Institute (IPAM), Brasilia, Brazil; 5 National Oceanic and Atmospheric Administration (NOAA), Boulder, CO USA; 6 CEMADEN, São Paulo, Brazil; 7 Institute of Advanced Studies, University of São Paulo, São Paulo, Brazil; 8 Rio de Janeiro State University (UERJ), Rio de Janeiro, Brazil; 9 Environmental Studies Department, University of California, Santa Cruz, Santa Cruz, CA USA; 10 University of Kansas, Lawrence, KS USA; 11 Department of Global Ecology, Carnegie Institution for Science, Stanford, CA USA

**Keywords:** Environmental impact, Atmospheric chemistry

## Abstract

The Amazon carbon sink is weakening as deforestation^1–5^, rising tree mortality^6–8^ and climate change disrupt the climatic conditions that sustain a humid tropical forest^9–11^. Forest loss reduces precipitation, increases temperatures and lengthens the dry season, driving higher carbon emissions^1–4,11,12^. Climate stress is stronger in more deforested regions, rendering forests progressively more flammable^1,9,13^. Large-scale climate events, such as El Niño, further amplify regional climate impacts^9,14–17^. Here we present an analysis of 15 years of Amazonian carbon fluxes derived from atmospheric CO_2_ measurements. We determine a carbon balance of 0.32 ± 0.08 PgC yr^−1^, with fires acting as the dominant source (0.40 ± 0.07 PgC yr^−1^) and forest uptake becoming increasingly unable to offset emissions. Furthermore, we investigate key drivers of the carbon emissions of Amazonia and identify three distinct year groups. Group 1 (2011–2014, 2017 and 2018) represents the role of the Amazon in the global carbon balance (0.11 ± 0.08 PgC yr^−1^) during wetter years with low human activity, such as deforestation, biomass burning and degradation. Group 2 (2019–2022) represents Amazon carbon emissions (0.43 ± 0.13 PgC yr^−1^) from human activities (mainly deforestation and degradation) during years with low climatic stress. Group 3 (2010, 2015, 2016, 2023 and 2024) has the highest carbon emissions (0.49 ± 0.07 PgC yr^−1^) and comprises warm and dry years (high climate stress), such as El Niño and the warming of the Tropical North Atlantic Ocean, associated with biomass burning.

## Abstract (web summary)

Fifteen years of atmospheric CO_2_ measurements show that the Amazon carbon sink is weakening, with fires, deforestation and climate stress driving increasingly high carbon emissions.

## Main

Amazonia, the largest tropical forest in the world, has lost substantial carbon stocks due to large-scale deforestation and other land-use changes. Remaining forests in the region have offset some of this carbon loss through carbon sequestration processes. However, this capacity is declining, primarily because of increased tree mortality^6–8,18^ and changes in regional climatic conditions modified by deforestation^1,3,9,13^. The Amazon forest stores approximately 123 ± 23 billion tonnes of carbon (PgC) in vegetation and soils^19,20^. The interactions among the forest ecosystem, hydrological cycle and regional climate are complex, with atmospheric moisture recycling by evapotranspiration accounting for up to half of precipitation^16^. Direct human activities within Amazonia, including deforestation, forest degradation and biomass burning, have profoundly altered the carbon balance of the region and climate^11^. Forest loss reduces precipitation, raises temperatures and lengthens the dry season, delaying the onset of the wet season^1,3,4,14,16^. During periods of extreme climatic variability, such as El Niño or anomalously high Tropical North Atlantic (TNA) sea-surface temperatures (SSTs), these impacts are amplified through the interaction of accumulated forest loss, global warming, changing circulation and large-scale anomalies^9,14,16^.

Many questions remain about the main drivers of carbon emissions and variability in Amazonia. Studies based on modelling and bottom-up techniques show increased emissions during extreme climate (warm and dry) years, pointing to extreme events as the reason for increased carbon emissions. However, our direct atmospheric measurements show a more complex situation. Here, we use our 15-year history of Amazonian carbon fluxes derived from atmospheric CO_2_ measurements to answer important questions about the main factors driving increased carbon emissions and variability in the Amazon and whether they are caused by global extreme climate events or direct human activities (deforestation, biomass burning and degradation) in the Amazon.

## Historic land use change

**Fig. 1:**
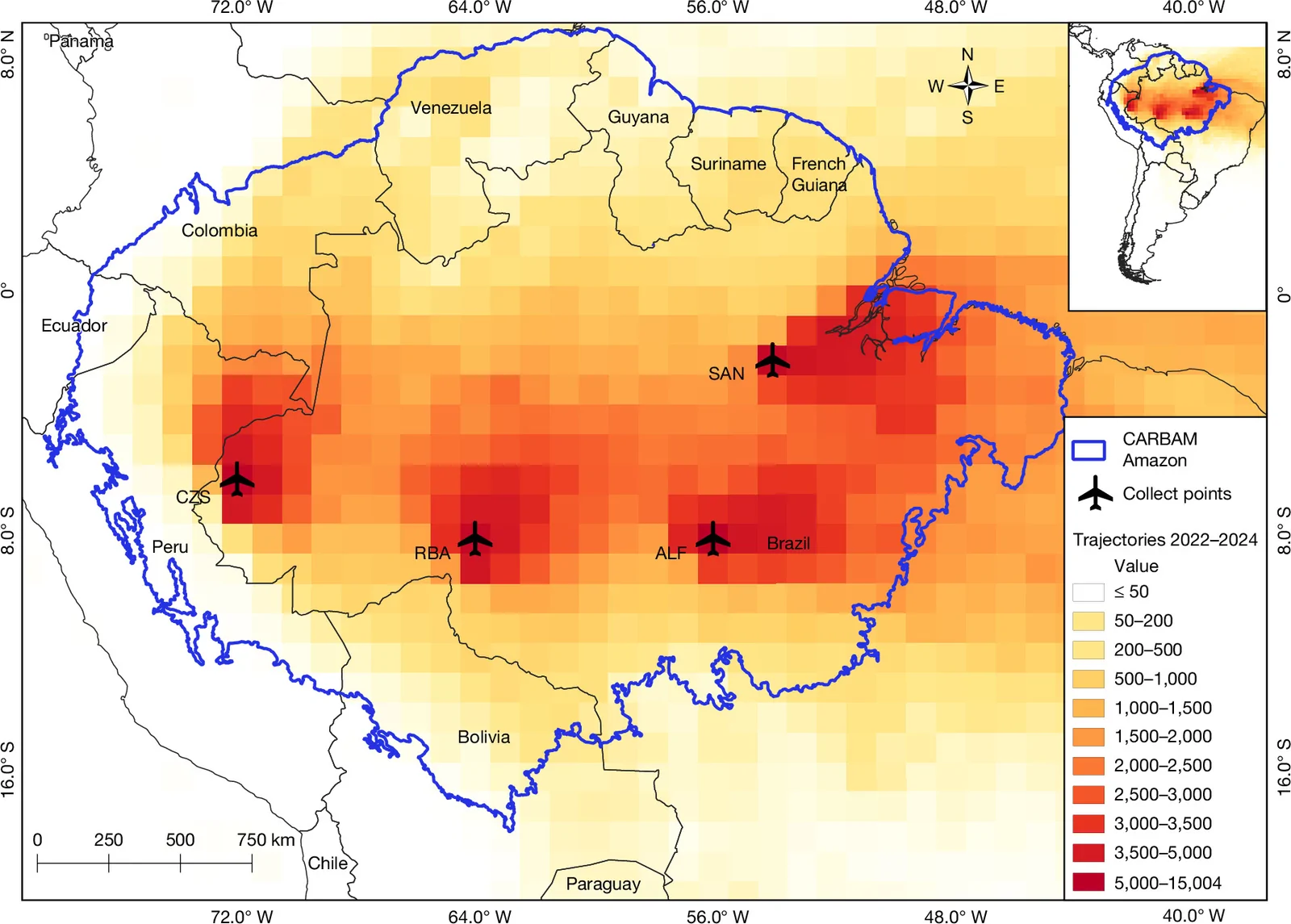
Aircraft sampling region of influence. Annual mean regions of influence, integrating RBA, ALF, SAN and CZS sites, based on back-trajectory density along the years 2022–2024, calculated by the HYSPLIT trajectory model for each flask (sample height). The blue line corresponds to Amazonia area of 6.7 × 10^6^ km^2^ adopted in this study (Methods). The regions of influence based on these four sites correspond to 95% of Amazonia. The regions of influence for the periods 2010–2012 and 2013–2021, as well as for each site, are presented in Extended Data Fig. 2.

In 2000, deforestation in the Brazilian Amazon represented a total area of 474,770 km^2^ (12.5% of the Brazilian Amazon, 3.8 × 10^6^ km^2^). The Brazilian Amazon represents 58% of the Pan-Amazonia region (Fig. 1). By 2004, the PRODES programme^21^ of Brazil for monitoring native vegetation had recorded cumulative deforestation of 612,225 km^2^ across the Legal Amazon, equivalent to 15.5% of the original forest cover of the region. Between 2004 and 2012, the annual rate of deforestation fell 84% as a result of public policies designed to curb clearing^22,23^, reaching a cumulative loss of 697,319 km^2^ (18.4% of the total forest area). The rate began to rise again from 2013 to 2018, when total forest loss reached 19.5%, and accelerated sharply after 2019, reaching 791,835 km^2^ (20.9%) in 2022. Although the annual rate of deforestation declined strongly in 2023 and 2024 (30% and 47%, respectively) relative to the 2019–2022 period, cumulative forest loss still reached 807,301 km^2^ (21.3%). From 2010 to 2024, pasture area expanded by 19%, soybean plantation area by 193% and mining by 272% (ref. ^24^) to estimated values of 561,416 km^2^, 59,368 km^2^ and 3,956 km^2^, respectively (Supplementary Fig. 1).

## Amazon precipitation and temperature changes

**Extended Data Fig. 1:**
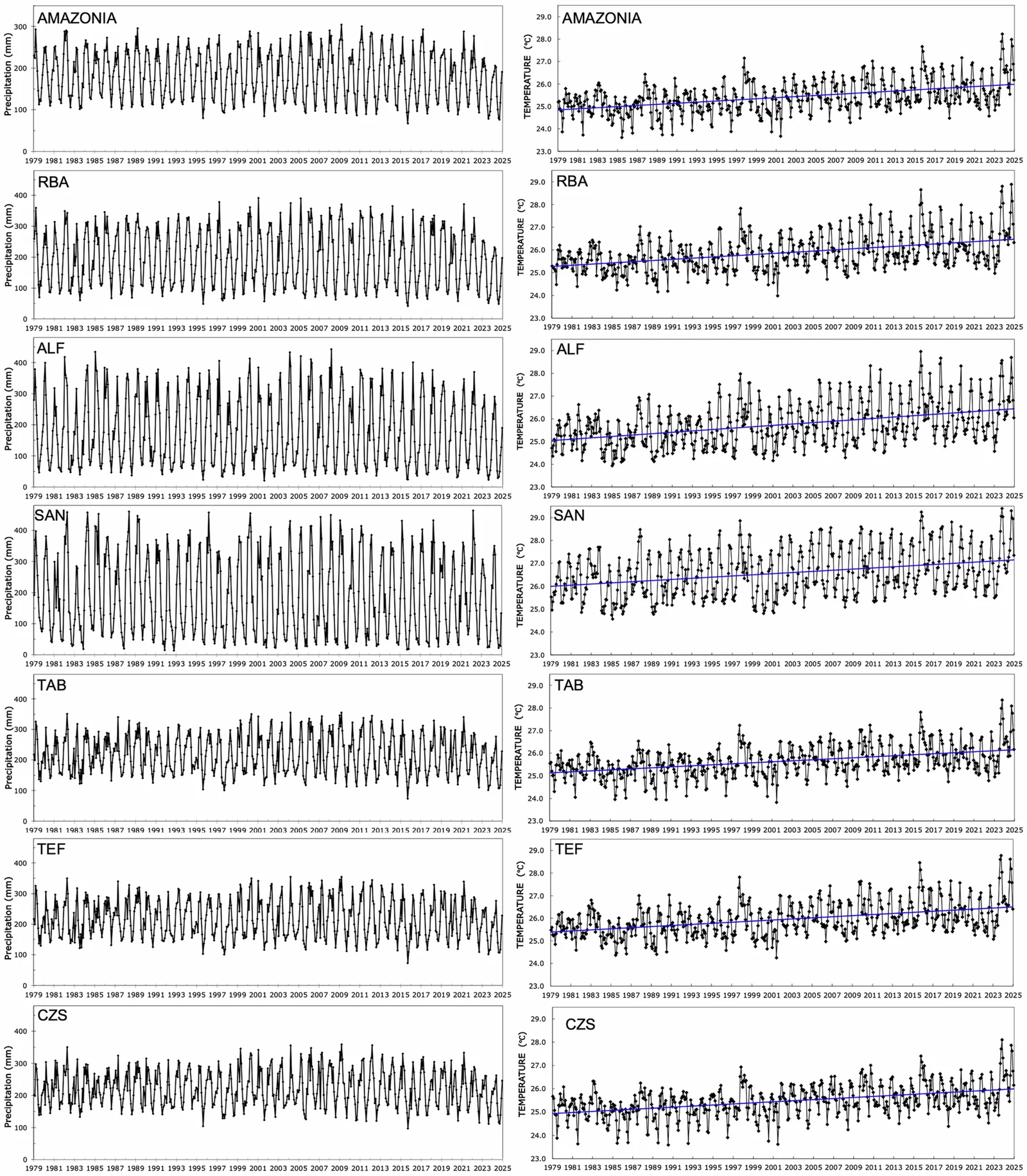
1979-2024 Amazonian precipitation and temperature. Amazonia monthly accumulated precipitation^54^, monthly mean temperature^27^ (Amazon mask area, Fig. 1 green line) for the period 1979 to 2024. Weighted monthly accumulated precipitation and weighted monthly mean temperature for the studied sites RBA, ALF, SAN, TAB, TEF, CZS (see regions in E. D. Fig. 1c–h), for the period 1979 to 2024 (see Methods), where it was calculated the weighted mean for each site using the region of influence from 2010–2024 as a spatial weighting function (see Methods).

We present precipitation and temperature time series from 1979 to 2024 in Extended Data Fig. 1. To assess the impact of the past six years, during which deforestation accelerated sharply between 2019 and 2022, on precipitation and temperature, we compared the previously studied period^1^ (1979–2018) with the current period from 1979 to 2024, using the same database for both periods (Methods). From 1979 to 2018, the changes in annual precipitation were not statistically significant (27 ± 69 mm); however, from 1979 to 2024, there was a reduction of 174 ± 69 mm, representing an 8% decrease. During the dry-season peak period (August–October), from 1979 to 2018, precipitation decreased by 17% (64 ± 19 mm), and from 1979 to 2024, by 20% (83 ± 18 mm). The increase in annual temperature from 1979 to 2018 was 0.93 ± 0.13 °C, and from 1979 to 2024 was 1.13 ± 0.13 °C, representing a 0.20 °C increase over 6 years. During the dry-season peak period, the increase in temperature was 1.51 ± 0.17 °C from 1979 to 2018 and 1.81 ± 0.18 °C from 1979 to 2024, representing a 0.30 °C increase over 6 years. Between 2018 and 2024, Amazonia lost an additional 60,964 km^2^ of primary forest and 564,532 km^2^ of burned area, representing a significant increase in human activity impacting the region since 2019. Further discussion of changes in climatic conditions in Amazonia and the studied regions is provided in Extended Data Fig. 1, Extended Data Table 1 and Supplementary Discussion 1.

Regional analyses show that the most pronounced temperature increase occurred in the southeast region (ALF), with warming of 2.19 ± 0.21 °C during the dry-season peak and 1.37 ± 0.15 °C in the annual mean between 1979 and 2024. From 2018 to 2024, this region lost 37,930 km^2^ (62% of the total deforestation of the Brazilian Amazon) of primary forest. The most intense decline in precipitation happened in the northeast region (SAN). From 1979 to 2024, annual precipitation decreased by 304 ± 150 mm (12%), and during the dry-season peak, it declined by 37% (77 ± 23 mm). Over the same six-year period, SAN lost 15,023 km^2^ (25% of the total deforestation) of primary forest. More details are presented in Extended Data Fig. 1, Extended Data Table 1 and Supplementary Discussion 3.

## Annual mean vertical profiles

**Fig. 2:**
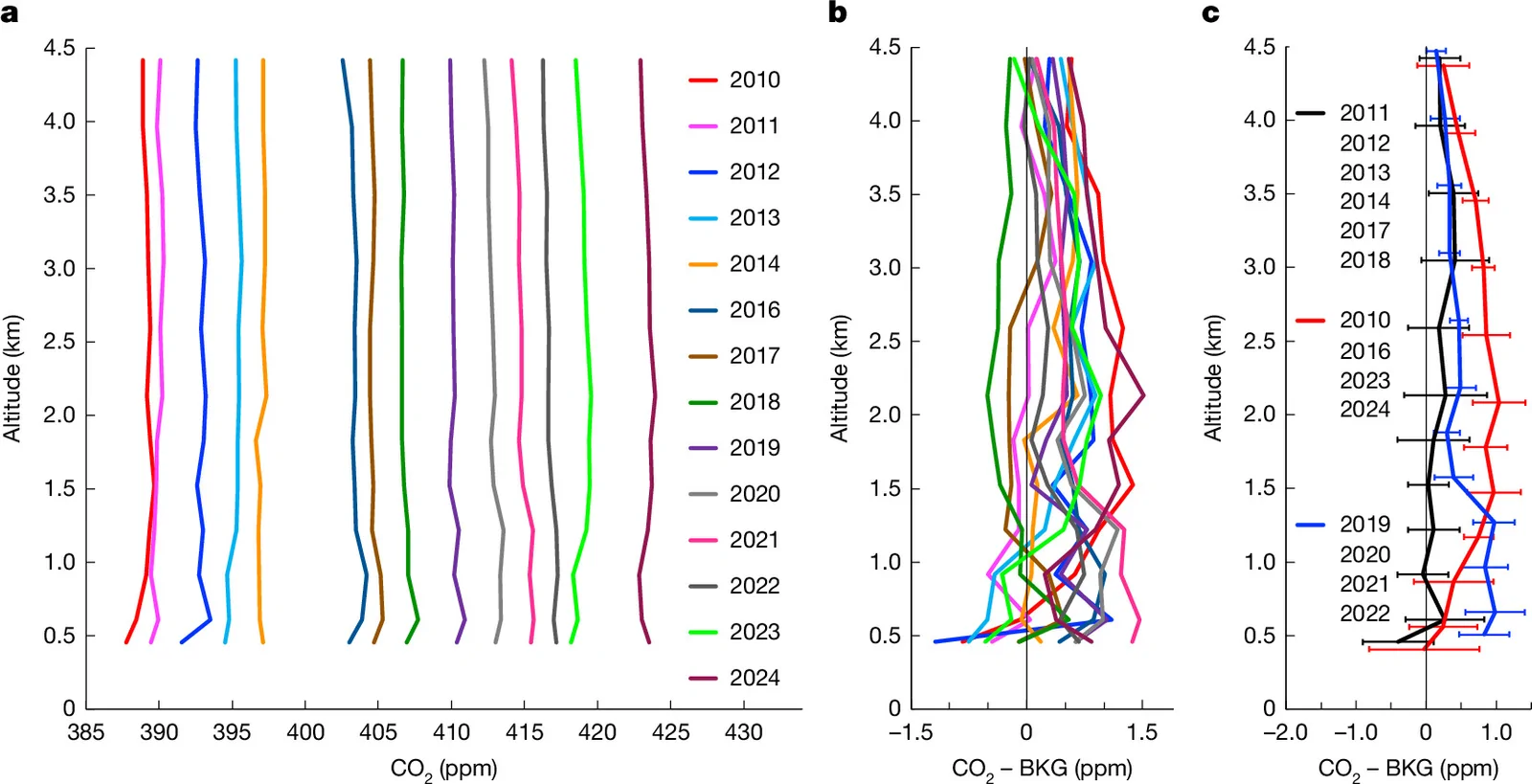
Amazon annual mean vertical profile. **a**, Amazon CO_2_ annual mean mole fraction (micromole of CO_2_ per mole dry air, or ppm) by height and by year. **b**, Same as **a**, but differenced from the CO_2_ background (BKG). **c**, Same as **b**, but multi-year means grouped according to statistical analysis of the years (group 1 of wet years and low human activities; group 2 of years with high human activities and low climatic stress; group 3 of dry and warm years and associated biomass burning) (Methods). The bars represent the standard deviation of the mean for the year.

**Extended Data Fig. 2:**
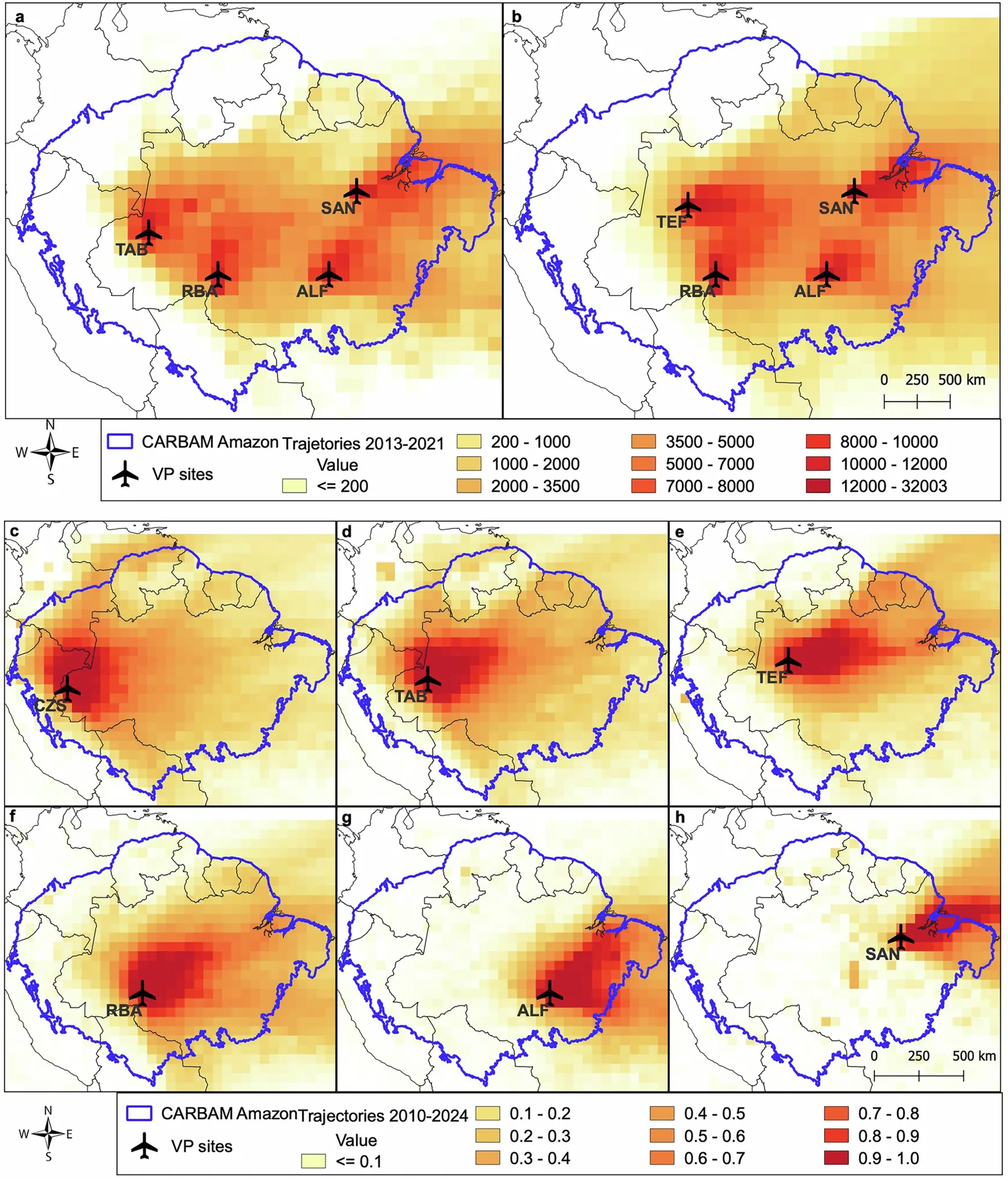
Amazonia and VP sites regions of influence. **a**, Annual mean regions of influence, integrating RBA (f), ALF (g), SAN (h), and TAB (d) sites, based on back trajectories density along the years 2010-2012, calculated by the HYSPLIT trajectory model for each flask (sample height). **b**, Similar to a, but the northwest site between 2013 to 2021 was TEF (h). The regions of influence were based on the back trajectory density along the years, but with additional upgrade in our method described in the methods. **c**, **d**, **e**, **f**, **g**, and **h**, present the annual mean for each region of influence from the sites CZS, TAB, TEF, RBA, ALF, and SAN, respectively.

**Extended Data Fig. 3:**
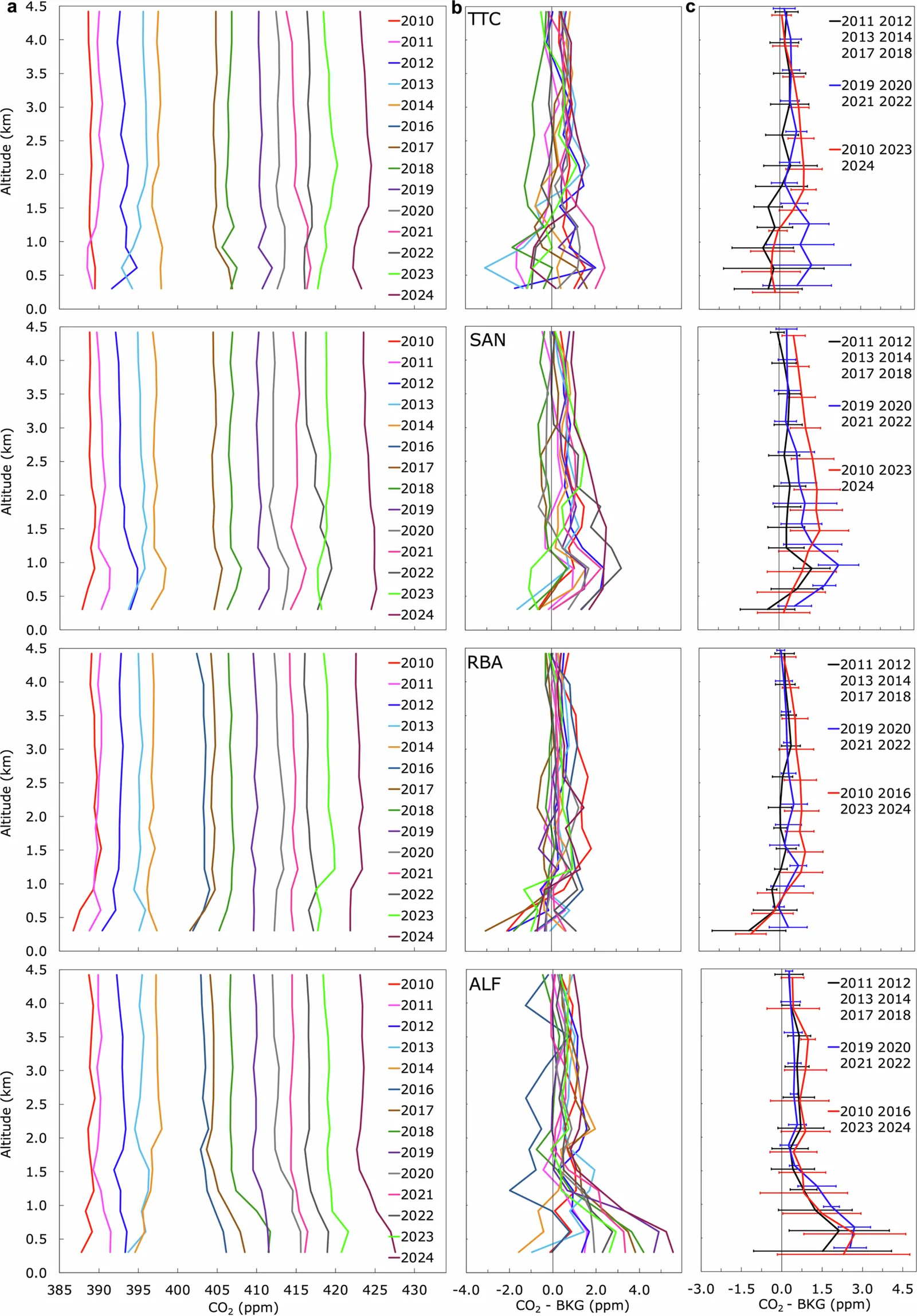
Annual mean VP, and ΔVPs per site. **a**, Annual mean VPs for each site TAB_TEF_CZS, SAN, RBA, and ALF for the time series (2010–2024), constructed from the annual mean VP (see Methods). **b**, Annual mean ΔVPs for each site for the time series (2010–2024), constructed from the annual mean VP, where the background was subtracted from each height, each flask (see Methods). **c**, Annual mean ΔVPs according the three groups of years presented in the right legend.

This study includes 1,017 vertical profiles conducted between 2010 and 2024 at four Amazonian sites, whose upwind areas of influence represent nearly the entirety of Amazonia (Fig. 1 and Extended Data Fig. 2). Vertical profiles were typically performed twice per month, from 4.4 km above sea level (a.s.l.) to near the surface (about 0.3 km a.s.l.), sampling air at 12 heights in a descending helical trajectory at each of the four sites between 12:00 and 13:00 local time. Air samples were analysed for CO_2_ and CO at the Greenhouse Gas Laboratory (LaGEE/INPE), São José dos Campos, Brazil. The Amazon exhibits a dominant lower-tropospheric circulation pattern, with air masses originating over the tropical Atlantic Ocean, crossing the Amazon towards the Andes, then to the southern regions, and returning to the Atlantic in the southern regions. This circulation enables integration of both natural and anthropogenic processes by computing atmospheric mass balance across vertical profiles of sites. Here, we present Amazonian annual mean CO_2_ vertical profiles (VP; Fig. 2a), and the profiles differenced from the CO_2_ background (∆VP; Fig. 2b) for 2010–2024, calculated from the annual mean profiles at each site (Extended Data Fig. 3), using the same weighting methodology used for Amazonia carbon balance calculations^1,13^ (Methods). Most years show a positive ∆VP near the surface (Fig. 2b), indicating that Amazonia is a net carbon source rather than a sink and cannot offset regional anthropogenic emissions.

## Carbon flux from column budget technique

**Fig. 3:**
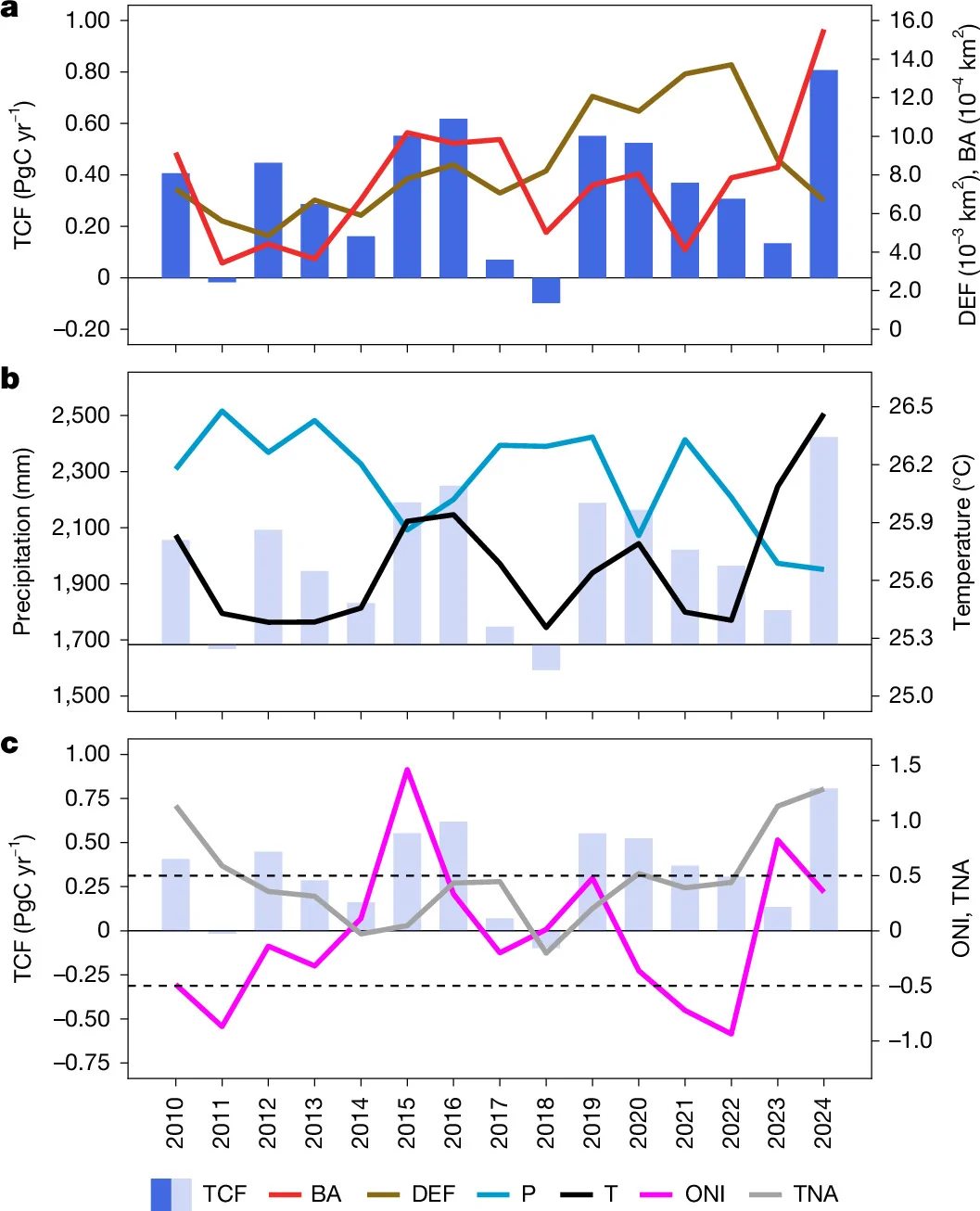
Amazon carbon balance and its drivers. **a**, Amazon annual mean TCF (blue bars, repeated as translucent blue bars in **b** and **c**), annual deforestation (DEF) from PRODES^21^ (brown line) and total burned area (BA) integrating all vegetation types^25^ (red line). **b**, Annual mean precipitation (cyan line) and annual mean temperature (black line). **c**, Annual mean ONI (pink line) and annual mean TNA index (grey line). The positive dashed line indicates years with values exceeding 0.5, corresponding to extreme El Niño and TNA events (Methods).

Carbon fluxes were calculated using the atmospheric column budget technique^1^ for each vertical profile and site over a 15-year study period. Total carbon flux  (TCF), fire carbon flux (FCF) and net biome exchange (NBE) monthly mean carbon fluxes (TCF subtracted by FCF = NBE) for each site were derived from the two vertical profiles collected per month, and Amazonia-wide monthly and annual mean fluxes were computed using a weighted mean approach that accounts for regional contributions^1,13^ (Methods). Figure 3 shows the Amazon carbon balance over 15 years, alongside some climatic and anthropogenic drivers. Examining the annual mean vertical profiles (Fig. 2) and the annual carbon balance (Fig. 3, blue bars) shows strong variability over the 15-year period.

## Results and discussion of grouping of years

**Extended Data Fig. 4:**
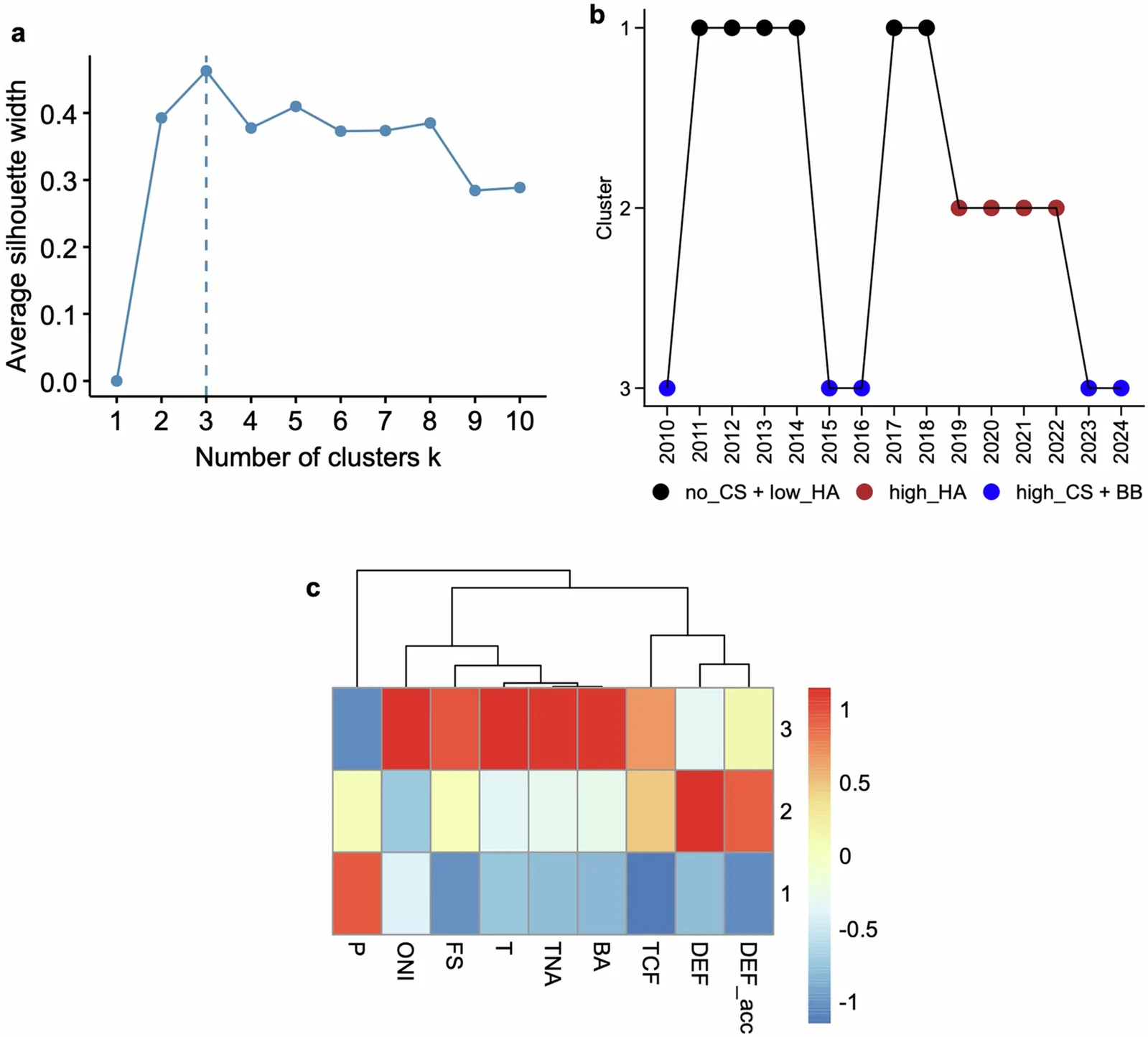
K-means clustering in annual variables. **a**, Determination of the optimal number of clusters using the silhouette method, indicating k = 3 as the best partition. **b**, Distribution of observations across clusters derived from K-means clustering (k = 3), based on the selected variables (TCF, P, T, ONI, TNA, BA, FS, DEF and DEF_acc), highlighting separation in the multivariate space. **c**, Heatmap of standardised variables (z-scores), showing distinct multivariate signatures across clusters, with contrasting patterns in precipitation (P), temperature (T), fire spots (FS), burned area (BA), deforestation (DEF), accumulated deforestation (DEF_acc) and carbon emission (TCF).

To better understand the variability in Amazonia annual carbon balance, we use *k*-means clustering analysis to group years, taking into account the following parameters: annual carbon balance (TCF), precipitation, temperature, deforestation (annual increment and accumulated), burned area (integrating all vegetation types)^25^, fire spots, Oceanic Niño Index^26^ (ONI) and TNA warming^27^. The cluster analysis identified the 15 years as belonging to three clusters: group 1 (2011, 2012, 2013, 2014, 2017 and 2018); group 2 (2019, 2020, 2021 and 2022); and group 3 (2010, 2015, 2016, 2023 and 2024). See the statistical analysis in Methods, Extended Data Fig. 4, Extended Data Tables 2 and 3, Supplementary Discussion 2 and Supplementary Table 1. Group 1 is characterized by wetter years and low human activities. Group 2 reflects a period with intense human activity—deforestation and degradation (biomass-burning, selective logging, mining and edge effects), and low climatic stress. Group 3 comprises years characterized by intense global climate events, such as El Niño and TNA events with associated biomass burning.

**Extended Data Fig. 5:**
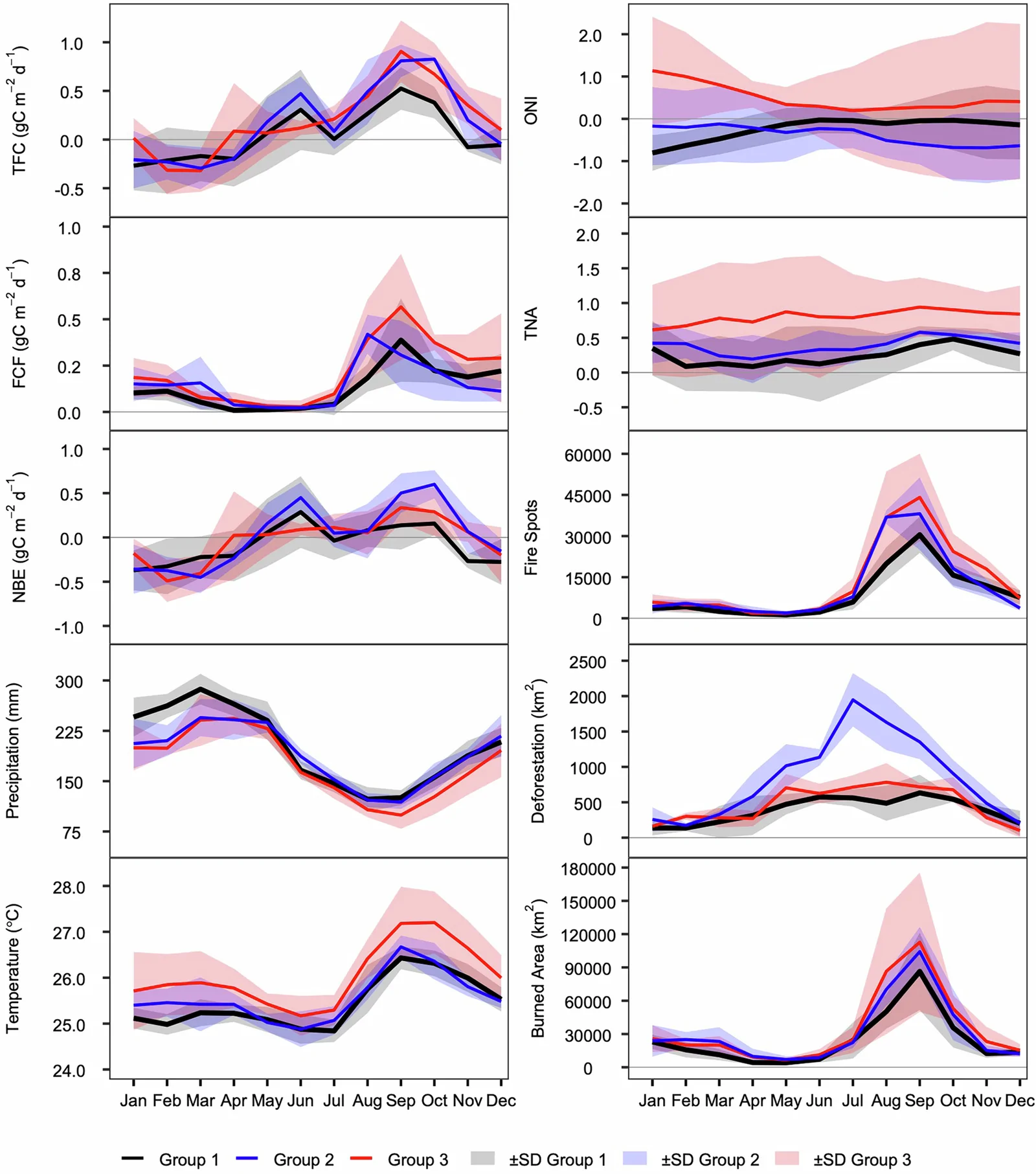
Seasonal Amazon C fluxes and drivers. Seasonal patterns of Amazonian carbon fluxes and drivers across the three groups. Seasonal monthly mean Amazonia Total C Flux (TCF), Fire C Flux (FCF) and NBE, and the group monthly mean for all drivers: precipitation^54^, temperature^27^, ONI^26^, TNA^27^, Fire Spots^63^, Deforestation^61^ (starting 2016), and burned area by MODIS^58^ (Amazonia area, blue line Fig. 1).

Group 1 (Fig. 2c, Extended Data Table 3a and Extended Data Fig. 5), representing the wettest years with low levels of human disturbance (deforestation and degradation), shows a near-neutral annual mean total carbon flux (TCF) of 0.11 ± 0.08 PgC yr^−1^ and ∆VP (VP – BKG) of 0.14 ppm, resulting from a carbon sink (−0.20 ± 0.12 PgC yr^−1^) that offsets 64% of fire emissions (0.32 ± 0.07 PgC yr^−1^). The annual deforestation and burned area were 6,389 km^2^ and 55,115 km^2^, respectively. This result indicates that Amazonia did not significantly contribute to the global carbon balance during wet years and with low human disturbance.

**Fig. 4:**
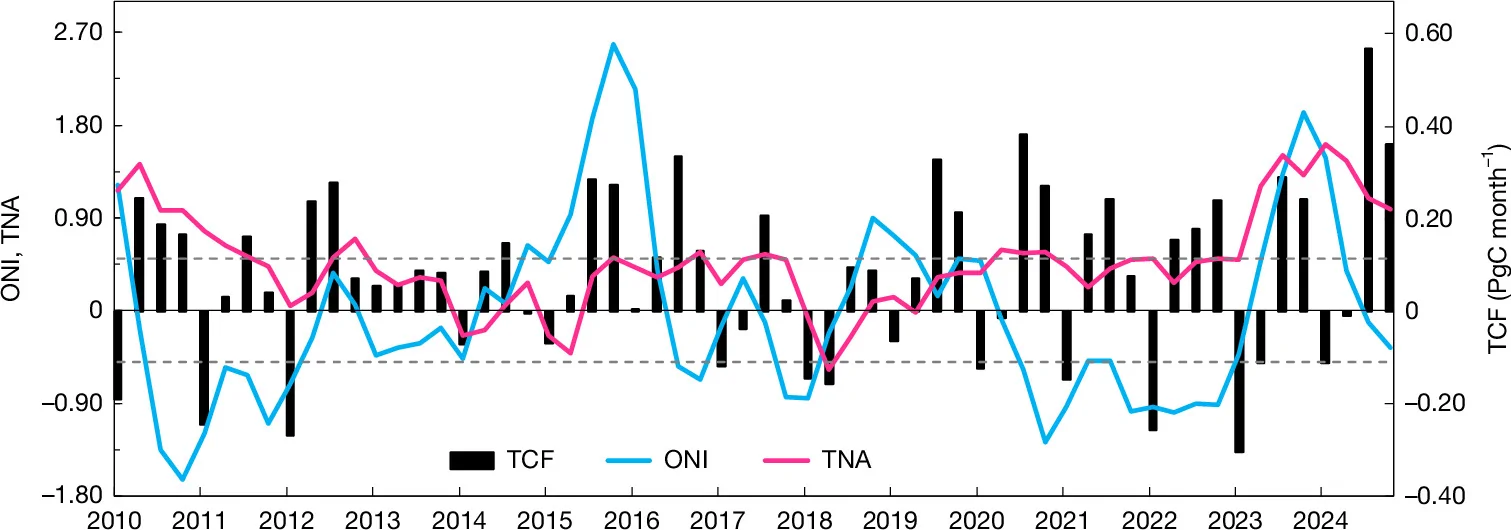
Quarterly Amazon carbon flux, ONI and TNA index. Quarterly mean TCF (black bars), ONI (cyan line)^26^ and TNA index (pink line)^27^ of Amazon. The positive dashed line indicates years with values exceeding 0.5, corresponding to extreme El Niño and TNA events.

Group 2 years (Figs. 2c and 3, Extended Data Table 3b and Extended Data Fig. 5) were characterized by a period with higher levels of human disturbance and weak governance^13^. During this period, the annual deforestation rate rose by 97%, whereas the burned area increased by 25%, resulting in a TCF of 0.43 ± 0.09 PgC yr^−1^ and a ∆VP of 0.53 ppm, representing increases of 291% and 379% relative to group 1, respectively. The annual mean temperature was the same as in group 1, and precipitation was 134 mm (6%) lower (mainly due to the 2020 wet season), indicating low climatic stress during this period. In August 2020, a strong and prolonged La Niña event began and lasted until February 2023 (Fig. 4). During this period, carbon uptake gradually increased in the first quarter of each year (Fig. 4), offsetting an ever-greater proportion of anthropogenic emissions. As climate stress was very low during this period, it mainly represents the carbon emissions from human activity in Amazonia.

**Extended Data Fig. 6:**
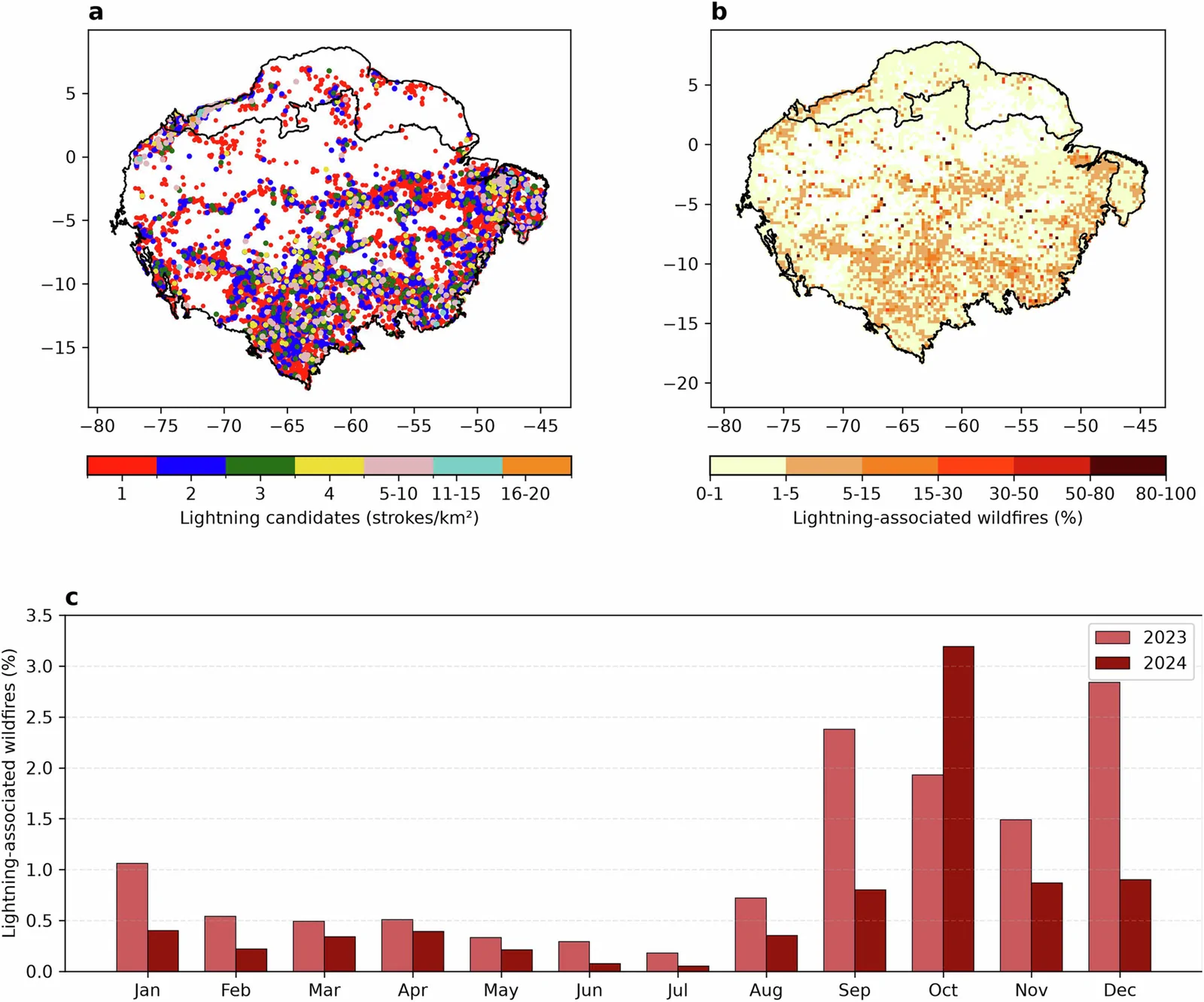
2023 and 2024 Amazonia lightning-induced ignition. **a**, Spatial distribution of lightning candidates with 1 km spatial resolution. Label bar values indicate the number of candidate strokes per km2. **b**, Lightning-associated wildfires with 25 km resolution. **c**, Monthly distribution of lightning-associated wildfires for Amazonia, from 2023 to 2024.

**Extended Data Fig. 7:**
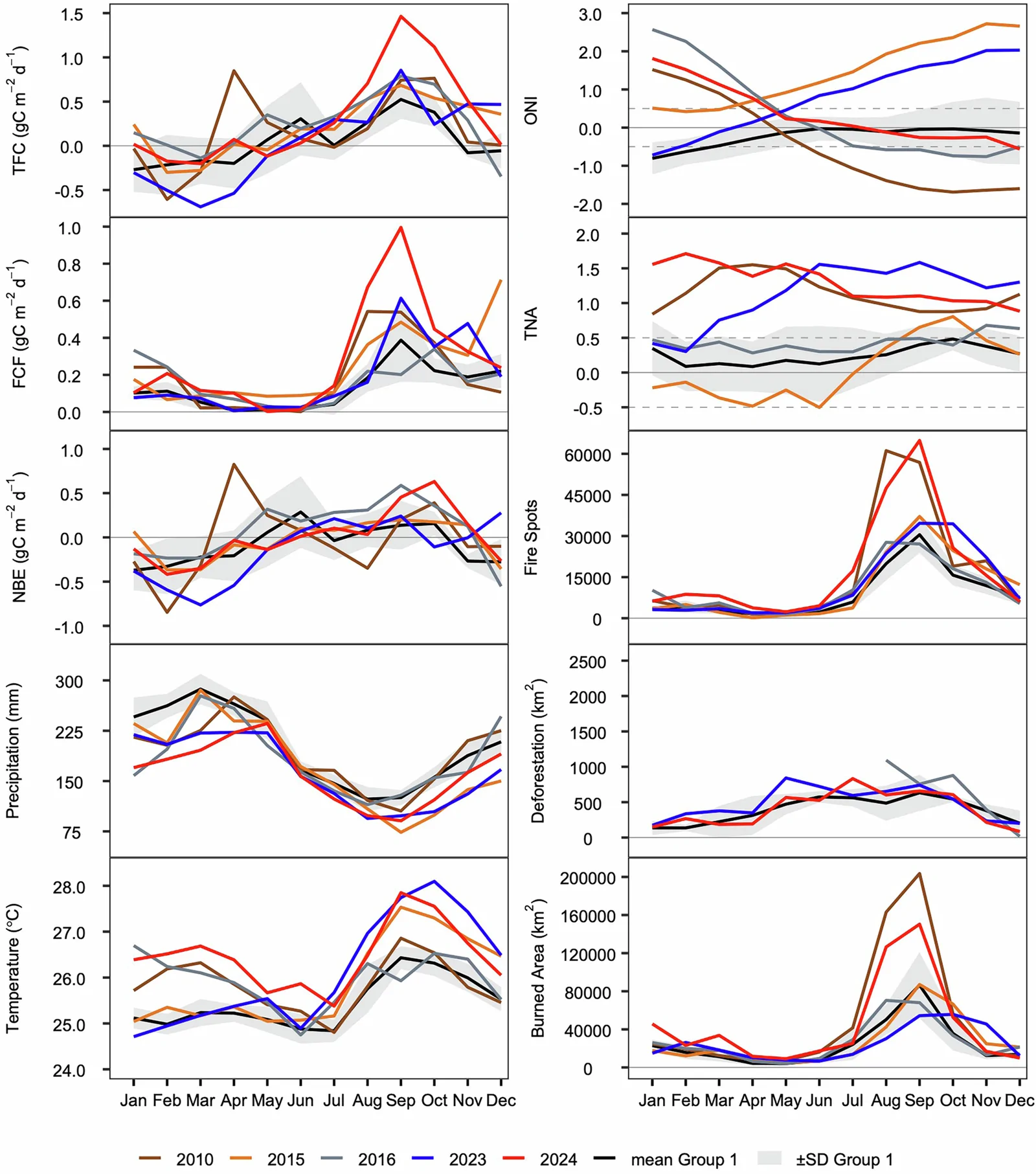
Group 3 Seasonal Amazon C fluxes and drivers. Seasonal behaviour of Amazon carbon fluxes and drivers for the years from Group 3 (2010, 2015, 2016, 2023, 2024). Seasonal monthly mean Amazonia Total C Flux, Fire C Flux and NBE, and the monthly mean for all drivers: precipitation^54^, temperature^27^, ONI^26^, TNA^27^, Fire Spots^63^, Deforestation (starting 2016)^61^, and burned area by MODIS^58^, values corresponding to the Amazon mask area – blue line in the Fig. 1.

Group 3 (Extended Data Table 3c and Extended Data Fig. 5) comprises years characterized by intense global climate events, such as El Niño and TNA warming. The mean ONI and TNA values for group 3 were higher than those for groups 1 and 2. Any years from groups 1 and 2 presented El Niño and/or warming TNA events. The El Niño events occurred in 2015–2016 (strong–very strong) and 2023 (strong; Fig. 3c), and the warming TNA events occurred in 2010 (moderate) and 2023–2024 (moderate–strong). We used the same ONI classification criterion^28^ for the TNA events (Methods). We also observed in Fig. 2c that the group 3 vertical profile showed an increase in the free-troposphere layer, unlike the other two; this is due to convective transport induced by the fire. Both El Niño and TNA warming contribute to reduced precipitation and increased temperatures in Amazonia. On average, group 3 received 308 mm (13%) less annual precipitation than group 1, with 20% less during the wet-season peak (January–March), and 17% less during the dry-season peak (August–October). The annual mean, wet-season peak and dry-season peak temperatures were higher than those of group 1 by 0.5 °C, 0.4 °C and 0.8 °C, respectively. These drought and warming conditions in Amazonia increased the flammability of the forest. We observed a 92% increase in burned area^25^ compared with group 1, whereas deforestation^21^ was 22% higher. TCF was 0.49 ± 0.07 PgC y^−1^, and ∆VP was 0.61 ppm, both of which were 325% higher than Group 1. However, for this group, NBE was −0.06 ± 0.10 PgC y^−1^ (nearly neutral sink), indicating that the main source of carbon is not the forest itself, but, again, human influence that promotes fires. Furthermore, recent climatic conditions interfere with the consequences of an intense El Niño year on the forest, as can be observed in 2023 and 2024 (Extended Data Tables 2 and 3 and Extended Data Fig. 7). FCF was 0.55 ± 0.10 PgC yr^−1^, 72% higher than group 1 and 53% higher than group 2, showing that fire emissions are an important characteristic of group 3. This finding highlighted the importance of clarifying the origin of fires, whether natural or anthropogenic. Therefore, a study was conducted to identify the relationship between lightning and forest fires in group 3. During the years of this group, it was found that lightning-induced ignition for fire events represented 1.6% and 1.0%, respectively, for the years 2023 and 2024 (Methods and Extended Data Fig. 6), allowing us to conclude that the emissions from group 3 originated primarily from human-induced fires, which were amplified by drought conditions. The years 2023 and 2024 were chosen to ensure gathering a more accurate cloud-to-ground lightning dataset, as higher-quality and more reliable data are only available from 2017 onwards.

**Fig. 5:**
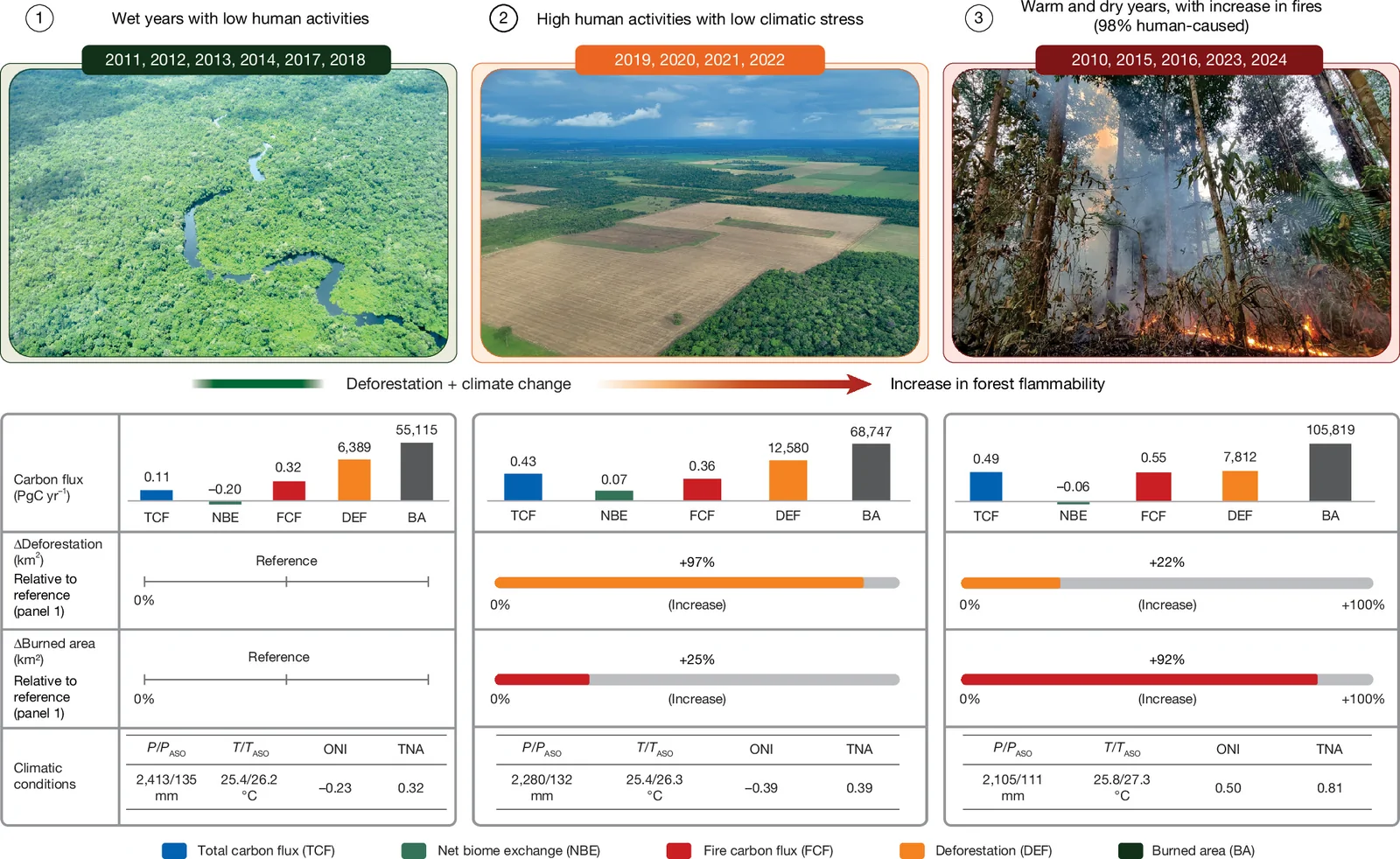
Schematic grouping summary. Schematic summary of the main results of the 15-year groups: (1) wet years, with low human activity of deforestation and burning area (0.11 ± 0.08 PgC yr^−1^); (2) low climatic stress years, with high human activity, mainly by deforestation (0.43 ± 0.13 PgC yr^−1^); and (3) warm and dry years, with high fire increase, 98% caused by humans (0.49 ± 0.07 PgC yr^−1^). *P*, annual accumulated precipitation; *P*_ASO_, monthly mean precipitation for August, September and October; *T*, annual mean temperature; *T*_ASO_, monthly mean temperature for August, September and October.

These findings underscore the dominant role of direct human disturbance in Amazonia (Fig. 5, a schematic grouping summary), driving year-to-year changes in carbon fluxes and highlighting the urgent need for mitigation to prevent the approach of the region to a climatic tipping point^29,30^. Climatic stress amplifies the effects of human-induced fire, because drought conditions substantially increase forest flammability^1,9–11,13,15,31–33^.

## The exceptional years 2023 and 2024

The 2023 wet season exhibited the largest carbon sink over our study period of 15 years (Fig. 4 and Extended Data Fig. 7), which occurred after three consecutive La Niña years (Fig. 4). The 2023 Amazonia carbon balance was similar to that of group 1 (0.11 ± 0.09 PgC yr^−1^), because the wet-season sink offset 75% of the carbon emissions from deforestation and burned area, which were, respectively, 38% and 52% higher than in group 1. The annual mean temperature was similar to that of group 1, starting colder than normal and warming progressively as strong TNA and El Niño events became unprecedented drought and heat wave^34,35^ (Fig. 4).

While 2023 began after 3 years of La Niña, 2024 began in the middle of strong El Niño and TNA events, which made its outcome completely different. The year 2024 exhibited the highest carbon emissions (0.81 ± 0.09 PgC yr^−1^) in the 15-year record (Extended Data Tables 2 and 3 and Extended Data Fig. 7), 605% higher than that of group 1. It was also the hottest, driest and most strongly degraded by fire over the past two decades^36^. Annual temperatures were 1.2 °C higher than in group 1, and annual precipitation was 462 mm lower than in group 1. The burned area was 182% higher than in group 1, producing the most extensive burned area observed since 2010 in Amazonia (Fig. 3a and Extended Data Tables 2 and 3). Following the reinstatement of public policies to curb deforestation in 2023 (ref. ^13^), fire became a widespread tool for deforestation. In 2024, PRODES identified that 38% deforestation was caused by fire^37^. Most Amazonian wildfires are intentionally set, and severe droughts exacerbate their extent and intensity^15,29,31,37^.

More detailed subregional (RBA, ALF, SAN, TTC) analyses are provided in Extended Data Tables 2 and 3, Extended Data Figs. 2 and 3, Supplementary Discussion 3 and Supplementary Figs. 3–5.

## Conclusion

The 15-year annual mean Amazonia carbon balance was 0.32 ± 0.08 PgC yr^−1^ (FCF, 0.40 ± 0.07 PgC yr^−1^; NBE, −0.08 ± 0.12 PgC yr^−1^). The pronounced interannual variability (−0.12 PgC yr^−1^ to 0.75 PgC yr^−1^) underscores the need for decadal-scale analyses to accurately define the role of Amazonia in the global carbon cycle, and it is also important to consider spatial representativeness, given the large variability across the subregions studied. Long-term deforestation and degradation have reduced annual precipitation by about 8% over the past 46 years and dry-season peak precipitation by about 20%, while increasing annual mean temperature by 1.13 ± 0.13 °C and dry-season temperature by 1.81 ± 0.18 °C. The grouping of years, in three distinct scenarios (Fig. 5), showed what Amazonia represents for the global carbon balance in each of these scenarios: (1) wet years, with low human activities (0.11 ± 0.08 PgC yr^−1^); (2) low climatic stress years with high human activities (0.43 ± 0.13 PgC yr^−1^); and (3) warm and dry years, associated with intense increase of fires, essentially human-caused (0.49 ± 0.07 PgC yr^−1^). It is important to note that groups 2 and 3 do not differ significantly in carbon emissions, and both are dominated by deforestation and biomass burning, respectively. It is also noteworthy that the NBE across all three groups is close to neutral, indicating that direct human activities are the main risks to the Amazon. Drastic fire reduction is therefore essential to avert a tipping point, especially as escalating climate stress amplifies human impacts, and vice versa. Based on the consequences of the very strong El Niño 2015–2016 in the southeast Amazon region, which has become an increasing source of carbon, similar consequences are expected after the strong El Niño and TNA events in 2023–2024 for Amazonia. These results highlight the urgent need to implement a zero-deforestation policy, strengthen controls on biomass burning and prioritize forest restoration programmes.

## Methods

### Sites, air sampling and analysis

Here, we report CO_2_ and CO measurement results from the four Amazonian aircraft vertical profile sites of the CARBAM project^38^ (SAN: 2.86° S 54.95° W; ALF: 8.80° S 56.75° W; RBA: 9.38° S 67.62° W; in 2010–2012 for TAB: 5.96° S 70.06° W; and since 2013 for TEF: 3.39° S 65.60° W, and since 2022 for CZS: 7.00° S 72.50° W), detailed in refs. ^1,13^. The regions of influence for the four sites representing the northeast (SAN), southeast (ALF), southwest (RBA) and northwest Amazon region (TAB, 2010–2012; TEF, 2013–2021; CZS, 2022–2024) are shown in Extended Data Fig. 2c–h. Our samples were typically collected twice per month, totalling 1,017 vertical profiles over 15 years, in a descending spiral from 4,420 m to about 300 m a.s.l. In 2015, the vertical profile sampling was stopped in April at all sites and resumed in November at RBA. In 2016, vertical profiles were performed only at RBA and ALF. The vertical profiles were usually taken between 12:00 and 13:00 local time. Air samples were analysed by a non-dispersive infrared analyser for CO_2_ and by gas chromatography with HgO reduction detection for CO. The detailed analytical and sampling methods were presented in previous studies^1,13^.

### Amazonia carbon study area

We defined the Amazonia study area according to subregions from ref. ^39^ and biomes from ref. ^40^, in which the studied area in Amazonia was determined to include only the forest ecosystems subregions: Amazonia stricto sensu, Guianas and Gurupi, with a total area of 6,700,798 km^2^.

### Annual mean vertical profiles

The annual mean VP for each site was calculated by first producing the monthly mean for each height and then the annual mean for each height. The annual mean ∆VP for each site and for Amazonia was calculated by subtracting the corresponding background (BKG) for each VP and each height (Fig. 2 and Extended Data Fig. 3). To calculate the Amazonia annual mean VP, we applied the same method used to obtain the Amazonia annual mean flux and balance. To scale to the whole of Amazonia, we divided it into three regions (see ref. ^13^ and Supplementary Fig. 5). To compose the VP for region 1 (SAN + ALF), we calculated the weighted mean CO_2_ concentration at each height, proportional to the area of each region. To calculate the VP for region 2 (RBA + TAB, 2010–2012; RBA + TEF, 2013–2021; RBA + CZS, 2022–2024), the same procedure was used as in region 1. And for VP of region 3, the remaining Amazonia area not covered by the regions of influence of the vertical profile, we used the same VP as region 2. To compose the VP for Amazonia, the weighted mean for each height CO_2_ concentration was calculated, considering the three regions for each height (Supplementary Fig. 5).

### Estimation of carbon fluxes

We used a column budget technique to estimate TCF, FCF and NBE, which consists of the difference between CO_2_ mole fraction measured in the vertical profile and the estimated background mole fraction^41^ (∆CO_2_), considering the travel time of air parcels along the trajectory from the coast to the site (equation (1)), following the methodology in refs. ^1,13,42^.

1 $${F}_{x}={\int }_{z=0(\mathrm{agl})}^{4.4\,\mathrm{km}(\mathrm{asl})}\frac{\Delta X}{t(z)}{\rm{d}}z.$$

### Background

Background mole fractions were estimated according to the methodology described in ref. ^41^, using the geographical position of each air-mass back-trajectory from the HYSPLIT model^43^ when it intersects two virtual limits: (1) a latitude limit, from the Equator southwards at 30° W and (2) a line between two observation sites at Ragged Point, Barbados (RPB), and Ascension Island, the United Kingdom (ASC), from NOAA/GML. We used CO_2_ (ref. ^44^) and CO (ref. ^45^) concentrations from the NOAA/GML Carbon Cycle Cooperative Global Air Sampling Network to determine the background.

### Carbon fire fluxes

We calculated fire emissions according to the methods published in refs. ^1,13^. We used observed CO/CO_2_ ratios by site from 2010 to 2024 (CO/CO_2_, ppb CO per ppm CO_2_) from measured CO mole fractions. The CO/CO_2_ ratios were calculated from clearly identifiable plumes in the vertical profiles during the dry season along period study (39 VP ALF CO/CO_2_ = 60.7 ± 30.7 ppb ppm^−1^; 35 VP SAN CO/CO_2_ = 56.1 ± 20.4 ppb ppm^−1^; 44 VP RBA CO/CO_2_ = 67.7 ± 24.1 ppb ppm^−1^; and 19 VP TTC (TAB_TEF_CZS) CO/CO_2_ = 74.2 ± 29.1 ppb ppm^−1^), which represents how many ppb CO is emitted for each 1 ppm CO_2_ during burning process. To calculate CO_2_ from fire, we used the CO flux and subtracted it from natural CO emissions (equation (2)). After the step ${F}_{\mathrm{CO}}-{F}_{\mathrm{CO}}^{\mathrm{Natu}{\rm{r}}\mathrm{al}}$, we used the CO/CO_2_ ratio to estimate the amount of CO_2_ from fire. NBE represents all processes from natural sources and sinks and all anthropogenic non-fire emissions (or after fire, without CO emissions) (NBE = total − fire).

2 $${{\rm{FC}}}_{{\rm{Fire}}}={R}_{{{\rm{CO}}}_{2}:{\rm{CO}}}({F}_{{\rm{CO}}}-{F}_{{\rm{CO}}}^{{\rm{Natural}}}).$$

### Flux uncertainty analysis by Monte Carlo error propagation

Flux uncertainties were estimated using Monte Carlo error propagation (1,000 iterations). CO_2_ measurement uncertainty was 0.03 ppm, and background (BKG) uncertainties were derived from residuals to the fitted CO_2_ smooth curve, according to ref. ^1^. Uncertainties in back-trajectory travel times^13^ were also included. For carbon fire emissions and NBE, uncertainties in CO/CO_2_ ratios, CO fluxes and a 25% uncertainty in natural CO emissions were considered. VP flux uncertainties were derived from the Monte Carlo ensemble. Monthly uncertainties were estimated using pooled standard deviation to account for intra-month variability within the annual mean flux, and period uncertainties were calculated using the quadratic mean.

An additional source of uncertainty is the limitation on sampling to 4.4 km a.s.l. Transit times of air parcels from the coast to sampling sites, based on HYSPLIT, can vary, with mean trajectory times of 2.6 days for SAN, 5.3 days for ALF, 6.6 days for RBA, 5.7 days for TAB, 5.0 days for TEF and 7.6 days for CZS. The convective process, together with long travel times of air masses, can represent loss of carbon sources and sink contributions from the surface, because our vertical profiles start at 4.4 km. Comparing the background concentration and the top of vertical profiles is one way to verify potential information loss^1,13^. According to the method we use, the flux is obtained by subtracting the measured CO_2_ mole fraction in the VP from the background concentration, accounting for the integration time (equation (1)). During the dry season, the loss of information is larger to positive (VP > BKG), because during the burning season (peak of the dry season), the top of VP starts with a higher CO_2_ concentration than BKG due to convective processes driven by biomass burning.

A previous study^46^ proposed a correction of our gas concentration measurements based on a comparison of laboratory analyses of samples collected on 12 vertical profiling flights above Manaus in 2017–2019. The in situ measurements were made with a cavity ring-down spectrometer (CRDS), and discrete flask samples (PFPs) collected on the same flights were analysed in the laboratory. Some of the continuous CRDS measurements differed from those determined in the laboratory from flask samples (PFP) collected on the same flights (Figure A4 in ref. ^46^). Based on linear regression of *N* = 158 paired CRDS-PFP measurements, ref. ^46^ reported that water vapour content explained only 13% of the variance in the CRDS-PFP differences (Figure A7 in ref. ^46^). Nevertheless, ref. ^46^ proposed applying this regression relationship to adjust all laboratory measurements. Applying such an adjustment would conflate multiple methodological differences between the two sampling and analytical systems. The regression in ref. ^46^ compares independent instruments sampling distinct air parcels under different pressures, flow rates and materials. The proposed correction assumes that the in situ CRDS measurements are accurate, that the laboratory analyses are biased, that discrepancies arise solely from water vapour, and that the regression relationship can be extrapolated to approximately 13,319 laboratory measurements collected over 15 years. For these reasons, the proposed adjustment was not applied in this study. LaGEE measurements used in this study are internally consistent across the full temporal and spatial domain.

An additional source of uncertainty is addressed in the calculation of fire carbon emissions and NBE. Over time, the forest is becoming drier and hotter. These changes promote a lower CO/CO_2_ ratio. The use of a single mean CO/CO_2_ ratio is another source of uncertainty, leading to lower fire emissions than those observed in recent years because the ratio tends to decline over time. It is likely that carbon emissions from fires were higher than our averages for 2023 and 2024 indicate, because both were drought years.

### Missing data imputation

We used the missForest algorithm to impute missing monthly TCF and FCF data at the ALF, SAN, RBA and TAB_TEF_CZS (TTC) sites. These gaps resulted from sampling and logistical issues in the laboratory. The missForest method is a non-parametric imputation approach based on random forests^47,48^, and we implemented it in R language^49^ through the missForest package^50^. To estimate missing values for TCF and FCF^1,13^, we included the following monthly variables in the imputation process: temperature (T), precipitation (P), vapour pressure deficit (VPD), ONI, TNA index, EVI, burned area (BA) and fire spots (FS). The missing data gaps were imputed using average values calculated from 1,000 runs of missForest. The normalized root mean square errors obtained for FC_Total_ and FC_Fire_ were 0.0014 and 0.0012 for ALF, 0.0011 and 0.0010 for RBA, 0.0055 and 0.0053 for SAN and 0.0018 and 0.0016 for TTC, respectively. NBE missing data were calculated by subtracting the fire from TCF.

### Regions of influence

We define regions of influence as those areas covered by the density of back-trajectories^51^. We updated the calculations for the regions of influence used in our published CO_2_ papers^1^. In this study, the regions received two treatment steps. In the first step, we calculated back trajectories for altitudes lower than 1,300 m. As we have only four heights below 1,300 m, the density of trajectories could be reduced, compromising the representativeness. To better characterize atmospheric circulation over the quarters and years, we calculated trajectories at each sampling height (>1,300 m) twice a week, at the same locations and time of day, over the 15 years for each VP sampling site. A second treatment in the grid cells was done to increase the weight for the grid cells near the sample site. We normalized the weight for the region of influence to be from 0 to 1. First, the grid with a value of 1 was calculated by disregarding 2.5% of the grids with the highest values, thereby increasing the area of the grid region with a value of 1. It was considered a regular grid of 1° × 1° generated in the WGS 84 georeference system (EPSG:4326).

### Precipitation, temperature, VPD and EVI data

We used the quarterly regions of influence maps as spatial weighting functions for all studied parameters to determine how each parameter influenced the carbon flux and for the missing data imputation process (missForest), following ref. ^4^. We used the GPCP database (http://eagle1.umd.edu/GPCP_ICDR/GPCP_Monthly.html), v.1.3, for precipitation analysis (described in refs. ^52–54^), which contains daily data at a 1° × 1° latitude–longitude resolution. We used 2-m temperatures from ERA-5 that are monthly means of daily means since 1959, and which were used with a resolution of 0.25° × 0.25° latitude–longitude, obtained from the European Centre for Medium-Range Weather Forecasts (ECMWF)^27,55^. The VPD product is a measure of the indirect vapour pressure deficit in kPa (resolution of 2.5 arcmin) of monthly means of temperature and humidity, provided by the Climatic Research Unit Ts4.0 (ref. ^56^). The dataset was resampled to a 1° × 1° spatial resolution using the monthly mean. The EVI is a vegetation index that aims to estimate the fraction of photosynthetically active radiation (fPAR) from terrestrial vegetation. The EVI product used^57^ was the MANVI: Moderate Resolution Imaging Spectroradiometer (MODIS) multiangle implementation of atmospheric correction nadir-solar (see the methods in ref. ^13^).

### Burned area

Two products of burned area (BA) were used: (1) for all annual burned areas, the BA from MapBiomas was used, integrating all types of vegetation that were burned (primary and secondary forest, pasture, agriculture and others), from the MapBiomas Fire Collection 4 (ref. ^25^; https://brasil.mapbiomas.org). MapBiomas Fire was validated with a global accuracy of 89% (ref. ^33^); (2) for monthly BA (used only for missForest calculation), MODIS products were used: MODIS Collection 6 MCD64A1 burned area product^13,58^.

### Deforestation

The procedures to retrieve deforestation as geographic data built by PRODES/INPE^21^ based on a historical series of Landsat images provide deforestation annual increments in the Brazilian Amazon. Detailed information on the PRODES methodology is available and can be accessed at http://urlib.net/rep/8JMKD3MGP3W34R/443H3RE (ref. ^59^). The dataset accuracy is 93.5 (ref. ^60^). We used the data period from 2010 to 2024. Using QGIS software, a 0.25° × 0.25° grid was generated for the entire Brazilian Amazon, which was filled with the absolute values of deforested area calculated for each cell and each year of the series. The mean deforestation area was calculated for the period 2010–2024 within each grid cell. Annual deforestation data correspond to the deforested area mapped by the PRODES project in a given year for Amazon forests.

### Monthly deforestation and degradation

Monthly deforestation and degradation data correspond to either the deforestation alerts (clear cut, deforestation with vegetation, and mining) or degradation alerts (ordered selective logging, disordered selective logging, burns and degradation) captured by the DETER^61^ Amazon system in a given month. This dataset is produced daily by the visual interpretation of medium spatial resolution optical imagery of National Institute for Space Research (INPE) and, although not a full estimate of deforestation and degradation in the Amazon, can indicate variations in deforestation patterns over time^33,62^. We used the raw version of the dataset provided by INPE that included both password-protected data and data collected from August 2015.

### Fire spots

Fire spots in Pan-Amazonia between 2010 and 2024 and burned area in Brazilian Amazon were retrieved from the BDQueimadas wildfire monitoring program of INPE^63^ (see the methods in ref. ^13^).

### ONI

Warm (El Niño) and cold (La Niña) periods based on a threshold of ±0.5 °C for the ONI, 3-month running mean of ERSST v.5 SST anomalies in the Niño 3.4 region (5° N–5° S, 120° W–170° W), based on centred 30-year base periods updated every 5 years (ref. ^26^). To analyse the intensity of the events, we used the ONI classification anomaly^28^—for El Niño events, weak (0.5–0.9 °C), moderate (1.0–1.4 °C), strong (1.5–1.9 °C), and very strong (≥2.0 °C).

### TNA index

The TNA index was computed as the mean sea-surface temperature (SST) anomaly over the region 23.5° N–5.5° N and 57.5° W–15° W (ref. ^64^). Monthly SST fields were obtained from ERA5 (0.25°), and anomalies were derived relative to a 30-year climatological baseline (1971–2000), consistent with standard TNA calculations^27^. We used the same ONI anomaly classification to analyse the intensity of the TNA warming events.

### Statistical analysis

Annual data (2010–2024) for the Amazon included TCF, precipitation (P), temperature (T), ONI, TNA index, fire spots (FS), burned area (BA), annual deforestation (DEF) and accumulated deforestation (DEF_acc). Variable distributions and homogeneity of variance were assessed using the Shapiro–Wilk and Levene’s tests (Supplementary Table 1), respectively. Although most variables met normality assumptions, robust statistical approaches were adopted because of the small sample size (*n* = 15) and potential deviations from Gaussian behaviour typical of environmental datasets (Supplementary Discussion 3). Principal component analysis was applied to standardized data, with the first two components considered to represent the dominant variability. The *k*-means clustering (*k* = 3) was performed in the principal component analysis space, with cluster characteristics summarized in a standardized heatmap. The optimal number of clusters was determined using the average silhouette width (Extended Data Fig. 4).

Group differences among clusters were assessed using Welch’s analysis of variance (Supplementary Table 1), which is robust to heteroscedasticity and unequal sample sizes. When significant differences were detected (*P* < 0.05), pairwise comparisons were conducted using the Games–Howell post hoc test (Supplementary Table 1), which does not assume equal variances and appropriately controls the type I error rate under these conditions. This approach provides a consistent and reliable framework for identifying differences among clusters in environmental variables (Supplementary Discussion 2). All analyses were conducted in R language^49^.

### Amazonia pasture, soybean and mining area

We obtained annual estimates of pasture, soybean cropland and mining area across Amazonia from the MapBiomas Collection 10^24^ dataset (30 m spatial resolution). Annual land-cover maps were used to isolate class-specific pixels (pasture, soybean and mining) and to compute their corresponding areas (km^2^) within the Amazonia carbon study area. This approach provided a consistent time series of land-use expansion and persistence for 2000–2024.

### Lightning-associated wildfires

After 2017, in group 3, the years with accurate data were 2023 and 2024, when a more accurate cloud-to-ground lightning dataset was available. We used cloud-to-ground strokes from the Earth Networks Total Lightning Network (ENTLN)^65^. In South America, particularly in Amazonia, ENTLN lacks a dense sensor network comparable to that in other regions of Brazil. Even so, it operates with a stroke detection efficiency above 50% (corresponding to a flash detection efficiency above 80%) and a mean location accuracy below 1 km. Just for reference, in regions with high sensor densities, after the 2021 upgrade, the ENTLN reaches cloud-to-ground flash detection efficiency levels above 95% and median location accuracy below 100 m (ref. ^66^). Although performance depends on regional sensor coverage, ENTLN is considered one of the most advanced and reliable lightning networks globally^67^. Active fires used in this study were provided by the Visible Infrared Imaging Radiometer Suite on the Suomi National Polar-Orbiting Partnership satellite, with a spatial resolution of 375 m. The Visible Infrared Imaging Radiometer Suite 375 m active fire product provides geolocation accuracy of the order of one pixel (about 375 m), with typical sub-pixel geolocation uncertainties (about 70 m), ensuring reliable spatial representation of fire locations. All overpasses, day and night, were used from the Wildfire Monitoring Program of the INPE, available at http://www.inpe.br/queimadas/ (ref. ^63^). To assess lightning-associated wildfire among all cloud-to-ground lightning strokes, we identified probable lightning candidates that occurred up to 72 h (3 days) before active fire detection, using a fixed buffer radius of 1 km around each active fire and accounting for the location accuracy of both datasets. A detailed description of lightning candidate characteristics in Brazil is provided in ref. ^68^.

## Online content

Any methods, additional references, Nature Portfolio reporting summaries, source data, extended data, supplementary information, acknowledgements, peer review information; details of author contributions and competing interests; and statements of data and code availability are available at https://doi.org/10.1038/s41586-026-11168-1.

## Supplementary information

Supplementary Information This file contains Supplementary Figs. 1–5, Supplementary Discussion 1–3 and Supplementary Table 1.

Peer Review File

## Figures and Tables

**Extended Data Table 1:**
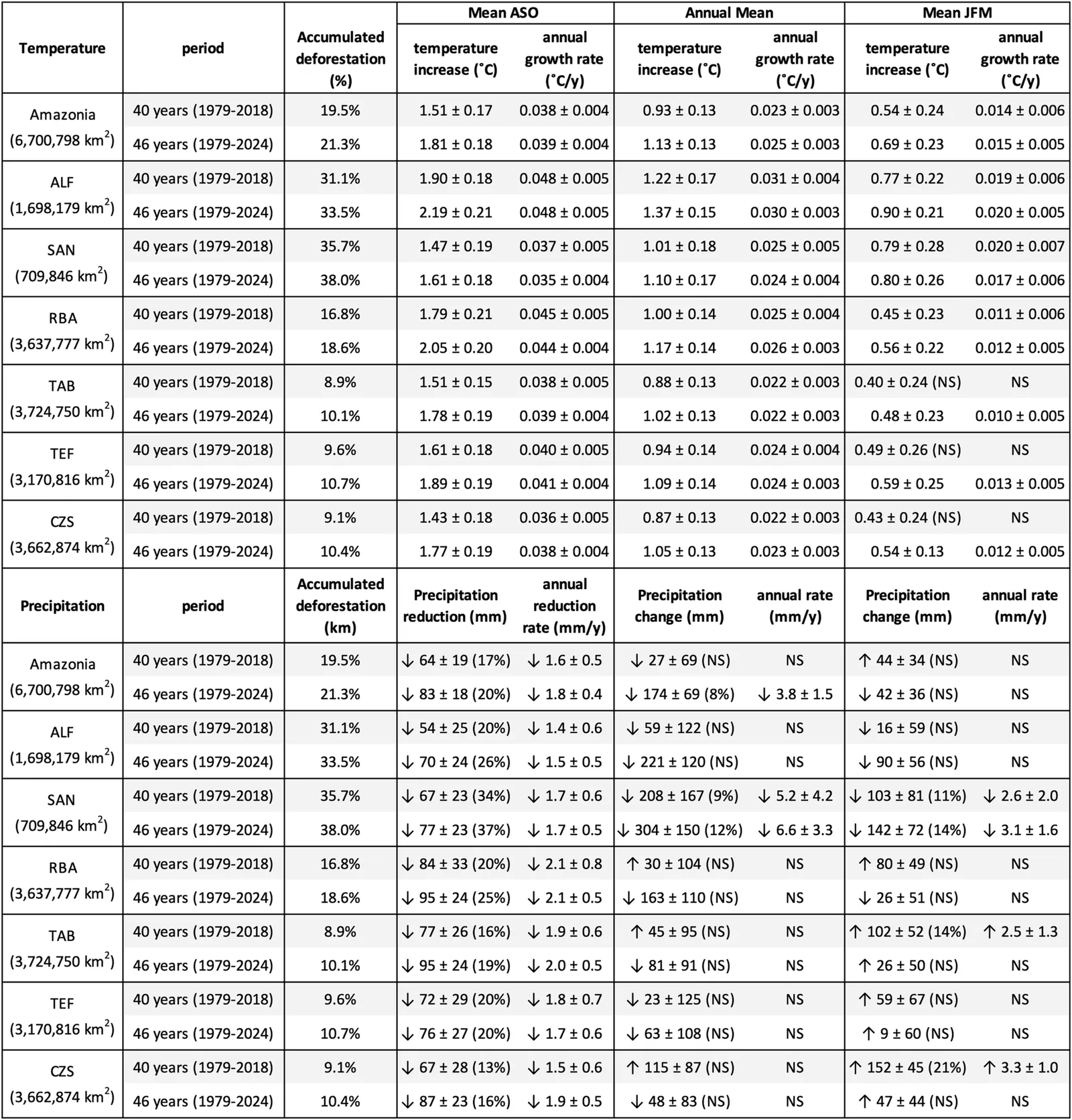
Amazon temperature and precipitation over the past 40 and 46 years

Observed trends for the periods 1979–2018, and 1979-2024, in annual mean temperature^27^, and accumulated annual precipitation^54^, for the whole Amazon, and the study sites RBA, ALF, SAN, TAB, TEF, CZS, using the region of influence from 2010–2024 as a spatial weighted method (see Methods). We also present trends for the wet (Jan, Feb, Mar) and dry (Aug, Sep, Oct) season peaks, as well as growth rates. Deforested areas for the sites were also calculated based on the weighted mean method. NS: non-significant.

**Extended Data Table 2:**
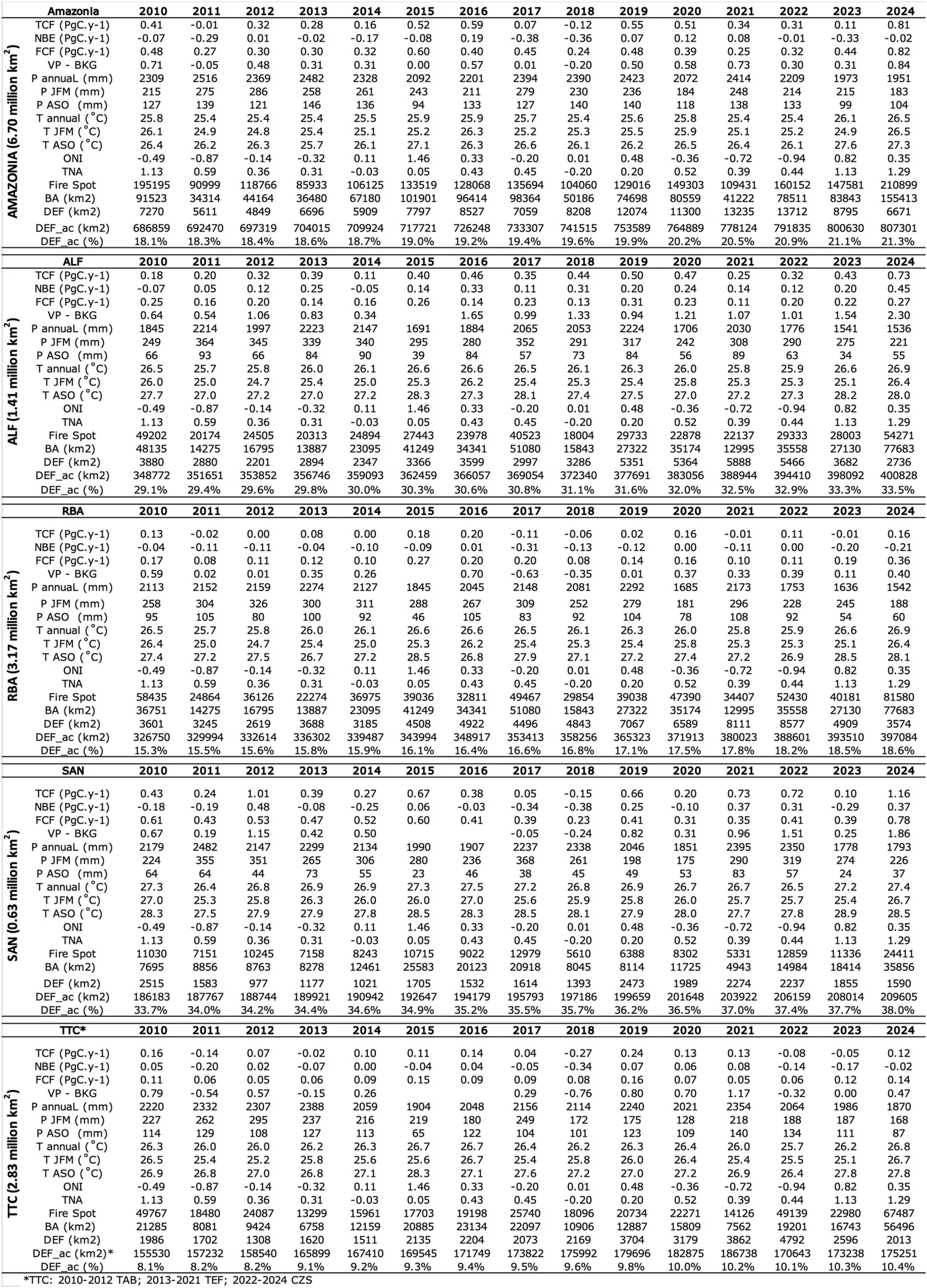
Annual Amazon and sites carbon flux and parameters

Results for all years presenting the carbon fluxes and the studied parameters: Total (TCF), Fire (FCF), and NBE, annual mean ∆VP, annual accumulated precipitation (P)^54^, monthly mean precipitation for the months Jan-Mar (P_JFM_), and for Aug-Oct (P_ASO_), annual mean temperature (T)^27^, monthly mean temperature for the months Jan-Mar (T_JFM_), and for Aug-Oct (T_ASO_), ONI (Oceanic Niño Index)^26^, TNA (Tropical Northern Atlantic Ocean anomaly (71-00) index^27^, Fire Spots (FS)^63^, Burned Area (BA)^25^, annual deforestation (DEF)^21^, accumulated Deforested (DEF_ac and in %) (see Methods).

**Extended Data Table 3:**
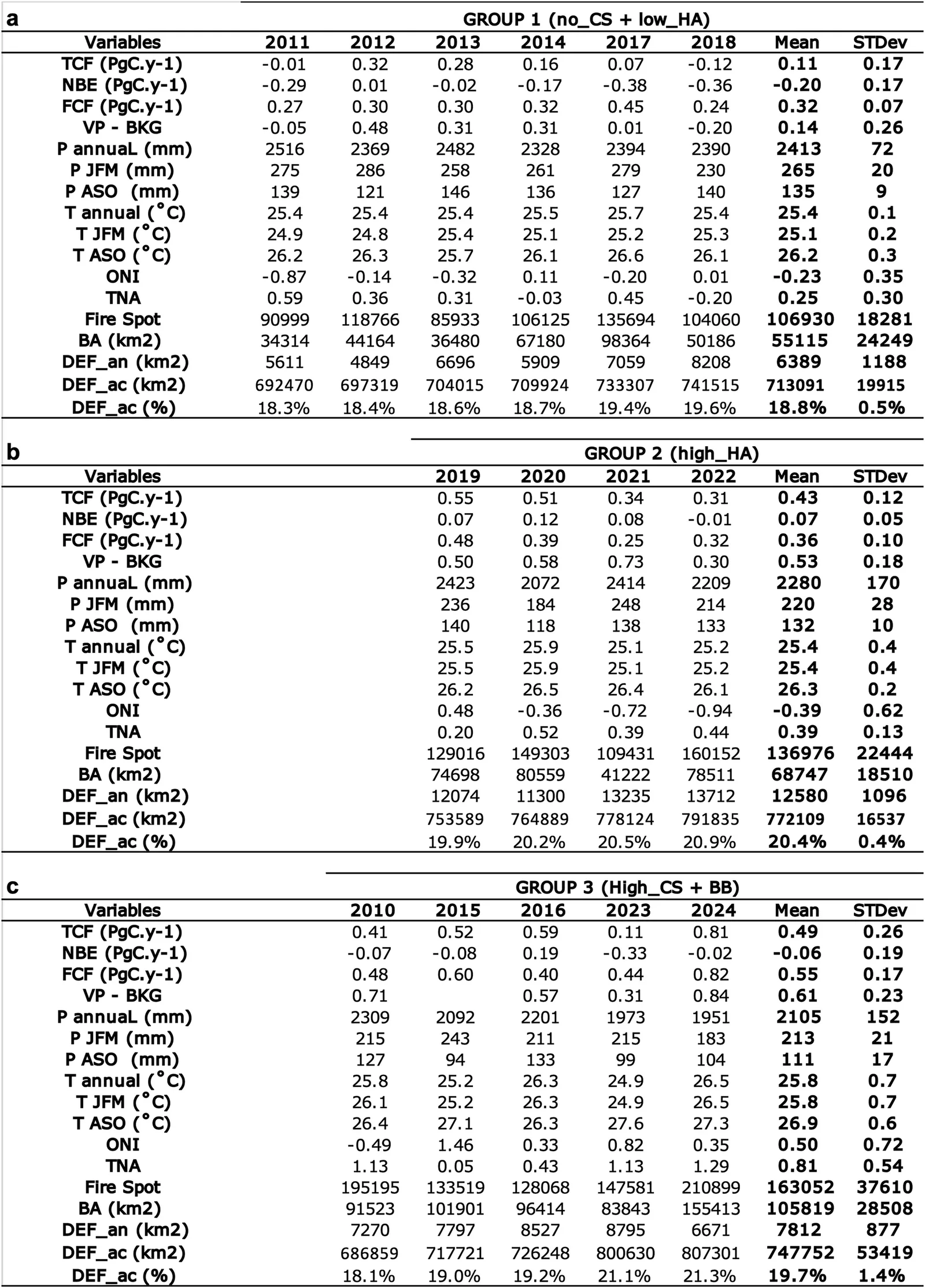
Carbon fluxes and studied parameters by groups

Group 1 represents years without climatic stress and low human activity. Group 2 represents high human activity and low climatic stress, and Group 3 represents high climatic stress associated with biomass burning. Items **a**, **b**, and **c** correspond to the Groups 1, 2 and 3, respectively, and present the annual and mean group carbon fluxes and the studied parameters. Total C Flux (TCF), Fire C Flux (FCF), NBE, annual mean ∆VP, precipitation (P)^54^, Jan-Mar mean precipitation (P_JFM_), and for Aug-Oct (P_ASO_), temperature (T)^27^, Jan-Mar mean temperature (T_JFM_), and for Aug-Oct (T_ASO_), ONI^26^, TNA^27^, fire spots (FS)^63^, burned area (BA)^25^, annual deforestation (DEF)^21^, and accumulated deforested (DEF_acc and in %).

## Data Availability

The CO_2_ and CO data, the calculated BKG CO_2_ and CO, and the time trajectory for each flask/height/day that were used to calculate the sites (TAB_TEF_CZS, SAN, ALF and RBA) and Amazon fluxes and balance that supported the findings of this study are available in the PANGAEA Data Archive, for data from 2010 to 2024 (https://doi.org/10.1594/PANGAEA.988417)^38^.
