# Supplementary material for: Amazon carbon emissions driven by human actions amplified by droughts
Source: Nature. 2026 Oct 21;658(8138):1188–93. doi: 10.1038/s41586-026-11168-1 (PMC13716835; doi:10.1038/s41586-026-11168-1)
Supplement: Supplementary file 1 [file 41586_2026_11168_MOESM1_ESM.pdf]

nature portfolio

https://doi.org/10.1038/s41586-026-11168-1

Supplementary information

Amazon carbon emissions driven by human actions amplified by droughts

In the format provided by the authors and unedited

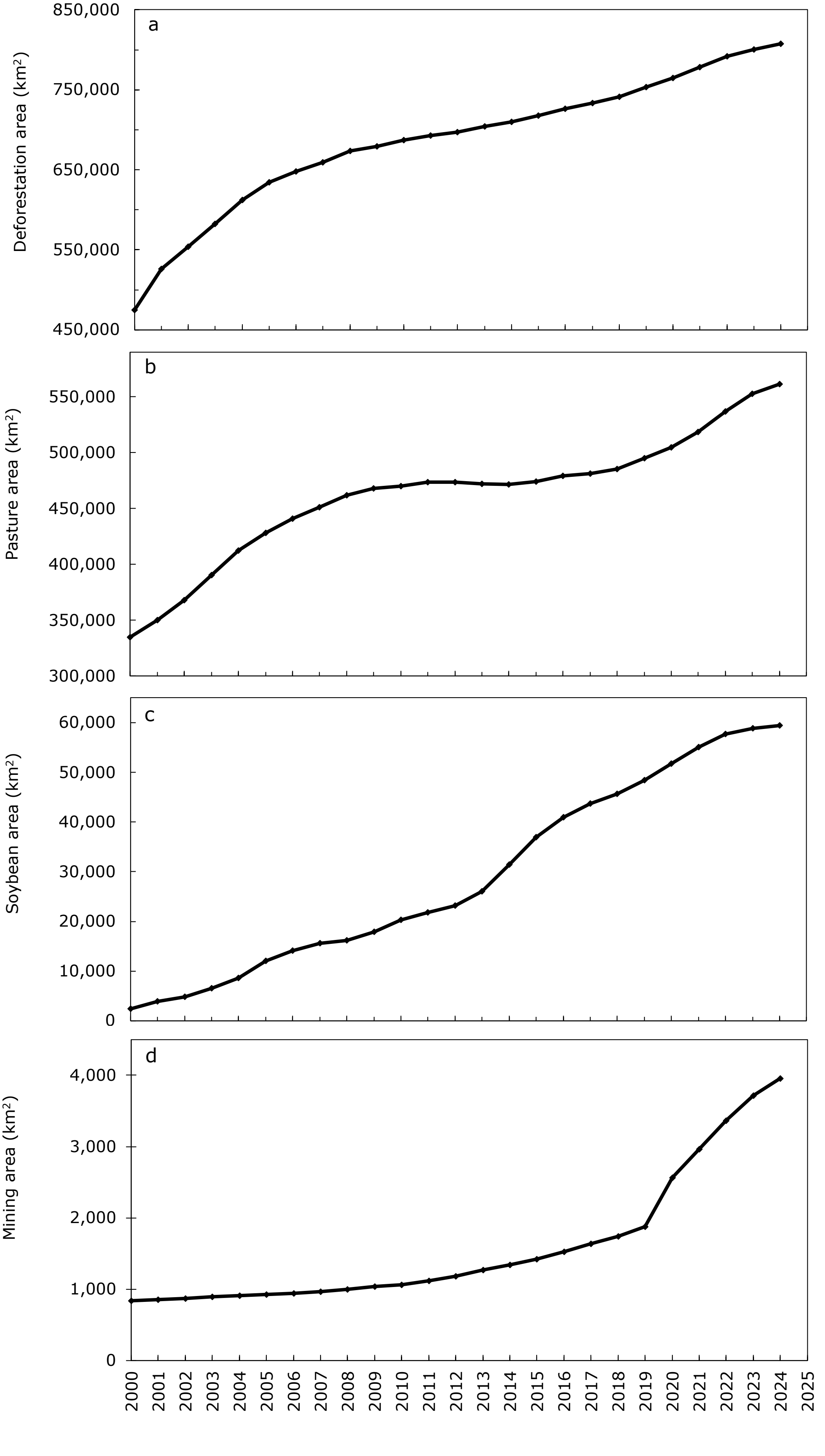

Supplementary Fig. 1 | Historic Amazon Land Use Change. a, Evolution of deforestation (PRODES)[21] in the Brazilian Amazon area, as limited in our study; **b**, pasture area for cattle raising; **c**, soybean plantation area; and **d**, mining area from 2000 to 2024 (MAPBIOMAS)[24].

Supplementary Discussion 1 | Climate Extremes in Amazonia

The Amazon has experienced intense droughts in 2005, 2010, 2015–2016, and 2023–2024, and intense floods in 2012 and 2021, each progressively more severe than the previous one[35,69]. Extreme climatic conditions such as drought are associated with El Niño events and a warmer tropical North Atlantic (TNA), or both[35,70]. On the other hand, La Niña means wet years in Amazonia. The El Niño 2023-2024 began in June 2023, following three consecutive La Niña events (from August 2020 to February 2023), and peaked in the austral summer of 2024.

Temperature increases in Amazonia, caused by deforestation and global climate change, have a significant impact on the region, including more severe droughts and floods, and intense heatwaves, which, when combined with drought, increase the risk of forest fires. The last decade has been the warmest. The warming trend has been most pronounced since 1980 and intensified since 2000[70]. In Amazonia, historical records show warming that is stronger from June to November, the peak season for fires and the onset of the wet season[4]. The higher warming rates in the eastern Amazon are attributed to changes in land cover and the resulting shift in the energy balance[71]. Both 2023 and 2024 were the warmest years in modern history, with intense heatwaves affecting Amazonia in both[72].

Rising temperatures and the intensification of extreme weather events, such as droughts, heatwaves and floods, are causing devastating damage to ecosystems and communities. Drought- and heat-induced changes have led to increased fire activity. The increases in deforestation, extreme wildfires, and the frequency of compound drought-heat events raise concerns that large portions of the Amazon Forest will experience significant degradation.

Supplementary Information References

69. Espinoza, J.-C., Marengo, J. A., Schongart, J. & Jimenez, J. C. The new historical flood of 2021 in the Amazon River compared to major floods of the 21st century: Atmospheric features in the context of the intensification of floods. Weather Clim. Extrem. 35, 100406 (2022).
70. Marengo, J. A. et al. The Drought of Amazonia in 2023-2024. Am. J. Clim. Chang. 13, 567–597 (2024).
71. Davidson, E. A. et al. The Amazon basin in transition. Nature 481, 321–328 (2012).
72. WMO. State of the Climate in Latin America and the Caribbean 2024. (2025).

Supplementary Discussion 2 | Statistical assessment of cluster differentiation

The Shapiro–Wilk test indicated that most variables were normally distributed, except for temperature ($p = 0.039$), suggesting a minor deviation from Gaussian behaviour (Supplementary Table 2a). Levene's test showed that homogeneity of variance was generally satisfied (Supplementary Table 2a), although total carbon flux (TCF) exhibited significant heteroscedasticity ($p = 0.020$). These results indicate that, despite overall compliance with parametric assumptions, violations were observed, warranting the use of robust statistical methods.

Cluster 1 (2011, 2012, 2013, 2014, 2017, and 2018) was associated with a strong impact of high precipitation, low TCF, fire spots, and accumulated deforestation. And moderately influenced by low temperature, TNA, burned area, and deforestation. Cluster 2 (2019, 2020, 2021, and 2022) is strongly influenced by deforestation (annual increment) and accumulated deforestation, and weakly influenced by all other climatic parameters (Precipitation, Temperature, ONI, and TNA) and biomass burning (fire spots and burned area). Cluster 3 (2010, 2015, 2016, 2023, and 2024) was characterised by the strong impact of ONI, temperature, TNA, burned area, fire spots, and low precipitation, and was moderately impacted by high total carbon flux. Overall, cluster structure was primarily driven by deforestation and fire-related processes, whereas large-scale climate variability (ONI and TNA) contributed less to group separation (Extended Data Fig. 4).

Significant differences among clusters were observed for TCF, precipitation (P), temperature (T), burned area (BA), fire spots (FS), deforestation (DEF), and accumulated deforestation (DEF_acc) (Welch's ANOVA, $p < 0.05$), whereas large-scale stressful climate events (ONI and TNA) did not show significant differences among groups (Supplementary Table 1). Pairwise comparisons (Games–Howell) showed that Cluster 3 was consistently distinct from Cluster 1, especially under conditions of low precipitation, high temperature and burned area, indicating that Cluster 3 represents a more fire-prone state with high carbon emissions. The comparison between clusters 1 and 2 revealed significant differences in deforestation and TCF. The differences between Clusters 2 and 3 are related to temperature and deforestation, suggesting that the difference in relation to human impact is concentrated in deforestation intensity.

Supplementary Table 1 | Statistical tests and cluster differences. **a**, Normality assessed using the Shapiro–Wilk test (H0: normality, H1: non-normality) and homogeneity of variances using Levene's test (H0: homogeneity, H1: non- homogeneity), applied to annual means of TCF, P, T, ONI, TNA, BA, FS, DEF and DEF_acc. **b**, Welch's ANOVA used to test for differences in group means among clusters derived from K-means clustering (k = 3), accounting for unequal variances. **c**, Games–Howell post hoc test (two-sided, 95% confidence level) performed for pairwise comparisons between clusters when significant differences were detected.

a

	Shapiro test (p-values)	Levene test (p-values)
	All years (n=15)	All years (n=15)
TCF	0.870	**0.020**
P	0.180	0.147
T	**0.039**	0.332
ONI	0.705	0.277
TNA	0.175	0.740
FS	0.312	0.416
BA	0.184	0.224
DEF	0.109	0.131
DEF_ACC	0.316	0.158

if p> 0.05 accept H0

b

variable	p-value
TCF	0.023
P	0.021
T	0.010
ONI	0.187
TNA	0.205
BA	0.048
FS	0.041
DEF	0.000
DEF_acc	0.005

c

variable	Cluster Comparison		Confidence interval (95%) lower bound	Confidence interval (95%) upper bound	p_adj
TCF	2	1	-2.3E-01	-2.0E-02	**0.023**
TCF	3	1	-2.9E-01	1.4E-03	0.052
TCF	3	2	-1.6E-01	1.3E-01	0.944
P	3	1	7.5E+01	5.4E+02	**0.017**
P	3	2	-1.6E+02	5.1E+02	0.313
P	2	1	-2.0E+02	4.7E+02	0.393
T	3	1	-9.8E-01	-2.1E-01	**0.008**
T	3	2	-9.1E-01	-5.3E-02	**0.031**
T	2	1	-4.6E-01	2.3E-01	0.557
ONI	3	1	-1.8E+00	3.7E-01	0.179
ONI	3	2	-2.2E+00	4.4E-01	0.190
ONI	2	1	-1.0E+00	1.3E+00	0.899
TNA	3	1	-1.4E+00	2.7E-01	0.175
TNA	3	2	-1.3E+00	4.2E-01	0.307
TNA	2	1	-5.5E-01	2.7E-01	0.591
BA	3	1	-9.7E+04	-4.5E+03	**0.033**
BA	3	2	-8.4E+04	9.6E+03	0.115
BA	2	1	-5.3E+04	2.5E+04	0.595
FS	3	1	-1.1E+05	1.7E+03	0.056
FS	2	1	-7.2E+04	1.2E+04	0.150
FS	3	2	-8.6E+04	3.4E+04	0.446
DEF	2	1	-8.3E+03	-4.0E+03	**0.000**
DEF	3	2	2.7E+03	6.9E+03	**0.001**
DEF	3	1	-3.2E+03	3.2E+02	0.111
DEF_acc	2	1	-9.3E+04	-2.5E+04	**0.003**
DEF_acc	3	1	-1.2E+05	4.8E+04	0.421
DEF_acc	3	2	-5.8E+04	1.1E+05	0.629

Supplementary Discussion 3 | The Subregions carbon flux

The measurements were performed in four sub-regions (northwest, northeast, southwest and southeast). Here, we analysed each subregion based on the results presented in Extended Data Tables 2 and Extended Data Figs. 2 and 3, and Supplementary Figs. 2, 3, and 4. The **southwest Amazon region (RBA)** is marked by the absence of climate stress, which during the dry season (<100 mm) usually still has enough precipitation for the forest, since the mean dry-season peak (Aug-Oct) for the period between 2010 and 2024 was 86 ± 20 mm and the temperature was 27.4 ± 0.6 ˚C. The mean RBA total carbon flux (TCF) from 2010 to 2024 was 0.06 ± 0.02 gC m^{-2} d^{-1}, representing near-neutral emissions; the mean fire carbon emission (FCF) was 0.16 ± 0.02 gC m^{-2} d^{-1}, and net biome exchange (NBE: TCF – FCF) was -0.10 ± 0.03 gC m^{-2} d^{-1}. Group 1 presented 2157 mm of annual precipitation, 92 mm during the dry-season peak period, an annual mean temperature of 26.2 ˚C and 27.3 ˚C during the dry-season peak period, neutral carbon emissions, and a carbon sink (NBE) of -0.13 ± 0.02 gC m^{-2} d^{-1}. This region, after 2019, began a new land-use change process involving three states (Rondônia, Amazonas, and Acre - AMACRO) to transform a large forest area into soybean and pasture activities, according to the agribusiness plan. At Supplementary Figs. 2, 3 and 4, it is clear that the increase in deforestation, which deforested a total of 30,344 km^2 during the period of Group 2 (2019-2022), was reduced to 4,909 km^2 in 2023 and 3,575 km^2 in 2024. During the Group 3 years, marked by El Niño and TNA periods, the lowest precipitation and highest temperatures were observed, leading to drought conditions. The year 2024 was the worst in the historical series, with a 28.5% reduction in annual precipitation compared with Group 1. The precipitation reduction during the wet-season peak (Jan-Mar) was 37%, and during the dry-season peak, it was 35%. The increase in Temperature, compared with Group 1, was 0.7 ˚C, 0.9 ˚C, and 0.8 ˚C, for the annual, wet-season peak, and dry-season peak, respectively. The strongest characteristic for this year, compared with Group 1, was a 289% increase in Biomass burning, representing a very large area of forest loss by fire (77,682 km^2). However, in 2023, public policies focused on preventing deforestation returned, resulting in a deforested area 33% higher than Group 1 but 35% lower than Group 2. The burned area was similar to that in Group 1, resulting in carbon emissions similar to those in Group 1.

The **southeast Amazon region (ALF)**, has the second highest carbon emissions in Amazonia (TCF: 0.37 ± 0.03 gC m^{-2} d^{-1}; NBE: 0.17 ± 0.05 gC m^{-2} d^{-1}; FCF: 0.20 ± 0.04 gC m^{-2} d^{-1}), and is the second most deforested region (33.5%). Over the past 46 years, in this region, the annual temperature has increased by 1.37 ± 0.15˚C, and during the dry-season peak, the increase was 2.19 ± 0.21˚C, the highest increase in the Amazon regions. Annual precipitation over the same period showed a 12% reduction (304 ± 150 mm) and a 37% decrease during the dry-season peak. From 2010 to 2024, NBE was a carbon source in almost all years. The exceptions were 2014 and 2010, which were carbon sinks (Extended Data Table 2). After the very strong El Niño 2015/16, this region has never again become a carbon sink.

Supplementary Discussion 3 | The Subregions carbon flux (continuation)

Observing the Extended Data Fig. 3 for ALF, it is clear that, looking at the annual VP (a) after this event, the CO_2 mixing ratio near the surface is always higher than the free troposphere CO_2 mixing ratio, indicating the region as a carbon source. The NBE (Extended Data Table 2) accounted for 42% of TCF from 2010 to 2018 and 50% from 2019 to 2024.

In 2024, under the worst climatic conditions and with severe degradation from human-induced fires (burned area 245% higher than in Group 1), the NBE reached a 62% TCF, indicating a significant change in the forest ecosystem in this region. This dramatic scenario underscores the need to declare a state of emergency in this region, with an immediate prohibition on deforestation (legal and illegal) and biomass burning, and prioritising forest restoration.

The **northeast Amazon region (SAN)**, which represents the region with the highest carbon emissions in Amazonia (annual mean: 0.47 ± 0.09 gC $m^{-2} d^{-1}$, NBE: 0.00 ± 0.11 gC $m^{-2} d^{-1}$, Fire: 0.47 ± 0.06 gC $m^{-2} d^{-1}$). This region is the most deforested (38%) and is under high human pressure from cattle ranching, soybean planting, logging, and mining. Precipitation over the last 46 years showed a 12% reduction (304 ± 150 mm) in the annual total, and decreases of 37% and 14% during the dry- and wet-season peaks, respectively. The annual temperature increased by 1.10 ± 0.17˚C, and during the dry- and wet-season peaks, the increases were 1.61 ± 0.18˚C and 0.80 ± 0.26˚C, respectively. Since Amazonia is very extensive, the climatic conditions do not always occur throughout the entire region, the classification of years for Amazonia is sometimes not adequate for each subregion. In Group 1, 2012, present strong carbon emissions, lower precipitation and higher temperatures. The TCF for the Group 1 was 0.30 ± 0.09 gC $m^{-2} d^{-1}$; NBE was -0.13 ± 0.11 gC $m^{-2} d^{-1}$, and FCF was 0.43 ± 0.06 gC $m^{-2} d^{-1}$. Removing 2012 from this group, TCF decreased by 50%, and NBE increased by 50%. The Group 2 showed a 72% increase in deforestation and a 262% increase in carbon emissions, if we exclude 2012 from the Group 1, and a 91% increase if we include it. Group 3 presented the largest burned area with fire emissions of 0.56 ± 0.04 gC $m^{-2} d^{-1}$. 2023 was the second lowest total carbon emission (0.10 ± 0.09 gC $m^{-2} d^{-1}$), and 2024 was the highest TCF observed over the 15 years (1.16 ± 0.09 gC $m^{-2} d^{-1}$) as a consequence of 220% increase in burned area during this year.

The **northwest region (TTC)** is the least deforested (~10.5%) and presents the lowest climatic stress. The annual mean: 0.05 ± 0.03 gC $m^{-2} d^{-1}$, NBE: -0.04 ± 0.03 gC $m^{-2} d^{-1}$, Fire: 0.09 ± 0.01 gC $m^{-2} d^{-1}$. This region presented a 124% increase in deforestation during 2019-2022 (Group 2), followed by a progressive decrease in deforestation, and a progressive increase in burned area in the two subsequent years (47% during 2023 and 388% during 2024), indicating the use of fire as a deforestation procedure.

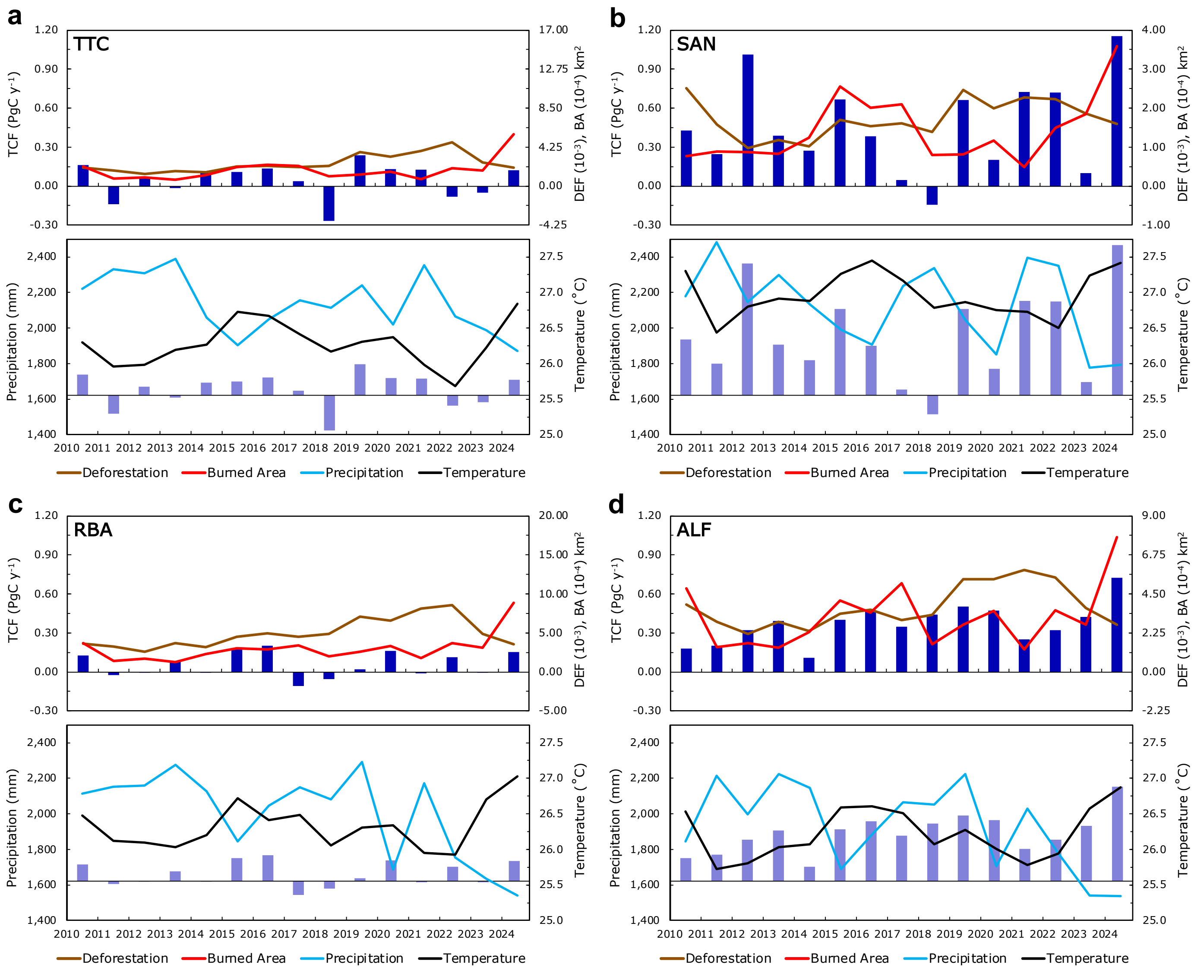

Supplementary Fig. 2 | Sites' annual mean Carbon balance and the drivers. The annual total carbon balance (blue bars) for the 4 sites TTC (**a**), SAN (**b**), RBA (**c**) and ALF (**d**), and the drivers: two anthropogenic (deforestation in brown line and burned area in red line), and two climatic (temperature in black line and precipitation in light blue line), see Methods.

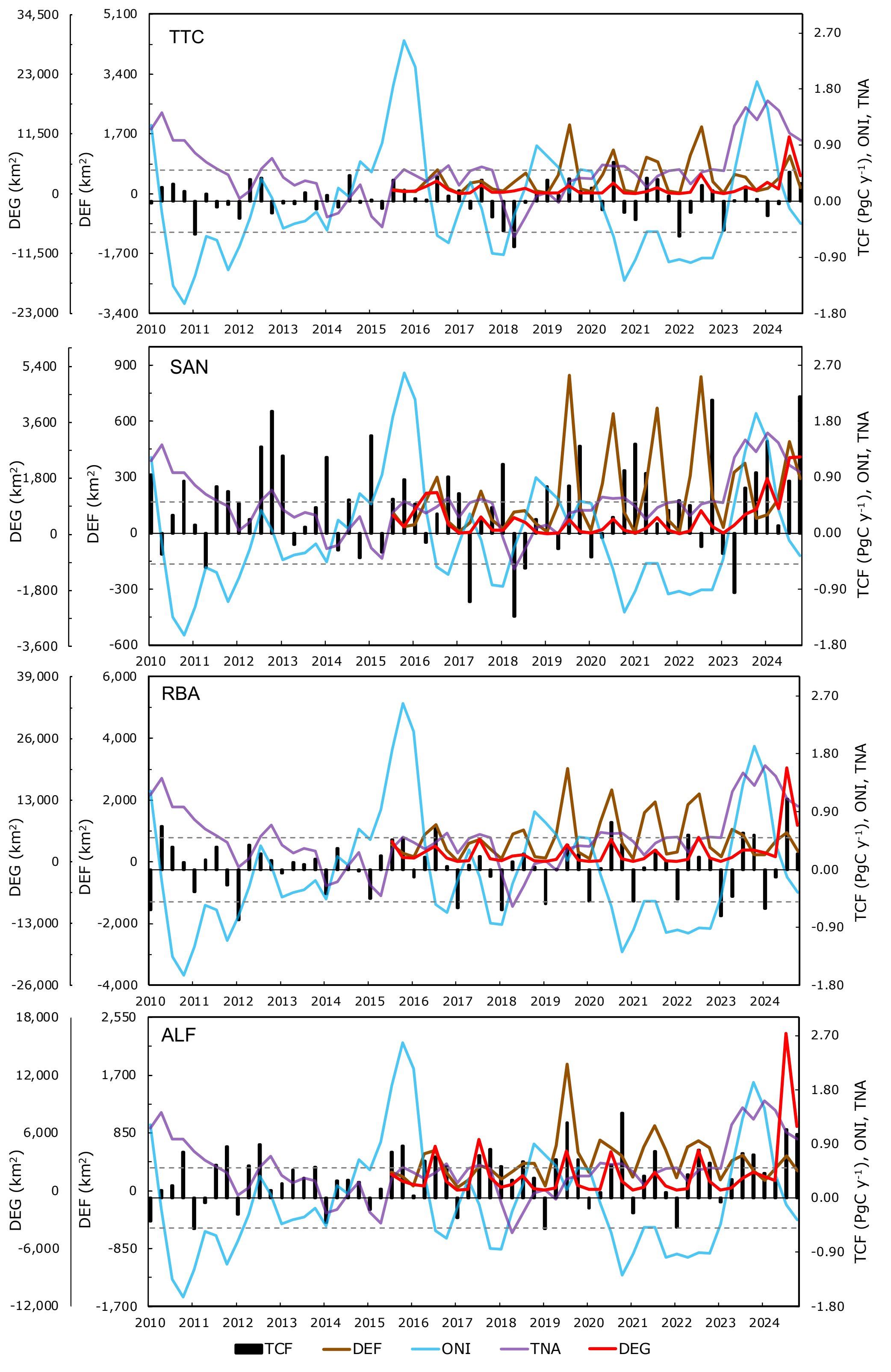

Supplementary Fig. 3 | Sites quarterly C flux and parameters. TTC (TAB_TEF_CZS), SAN, RBA, and ALF quarterly mean total C Flux from 2010 to 2024 (black bar), Oceanic Niño Index (ONI)[26] (light blue line), and Tropical Northern Atlantic Ocean Index (TNA)[27] (purple line), Degradation (brown line) and deforestation from DETER[61] (red line) was calculated for each site using the weighted mean method (see Methods).

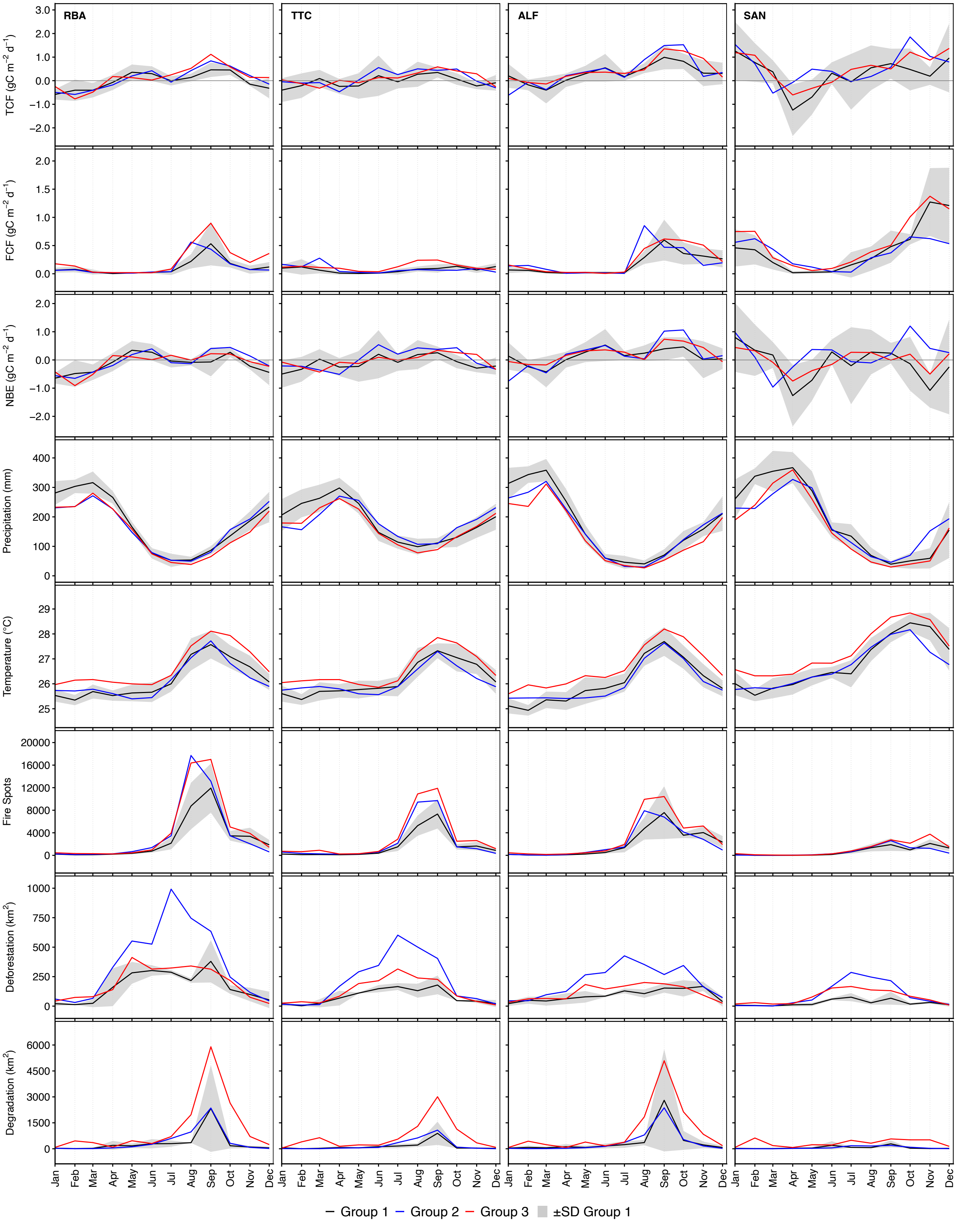

Supplementary Fig. 4 | Seasonal C Fluxes and parameters by Groups. Seasonal monthly mean Total C Flux, Fire C Flux and NBE for RBA, TTC (TAB_TEF_CZS), ALF, and SAN, and the weighted mean precipitation[54], temperature[27], fire spots[63], deforestation and degradation (available data from 2016)[61], all parameters subdivided by the three groups (in black line Group 1: 2011, 2012, 2013, 2014, 2017, 2018; in blue line Group 2: 2019, 2020, 2021, 2022; in red line Group 3: 2010, 2015, 2016, 2023, 2024), and in grey the variability of Group 1.

a

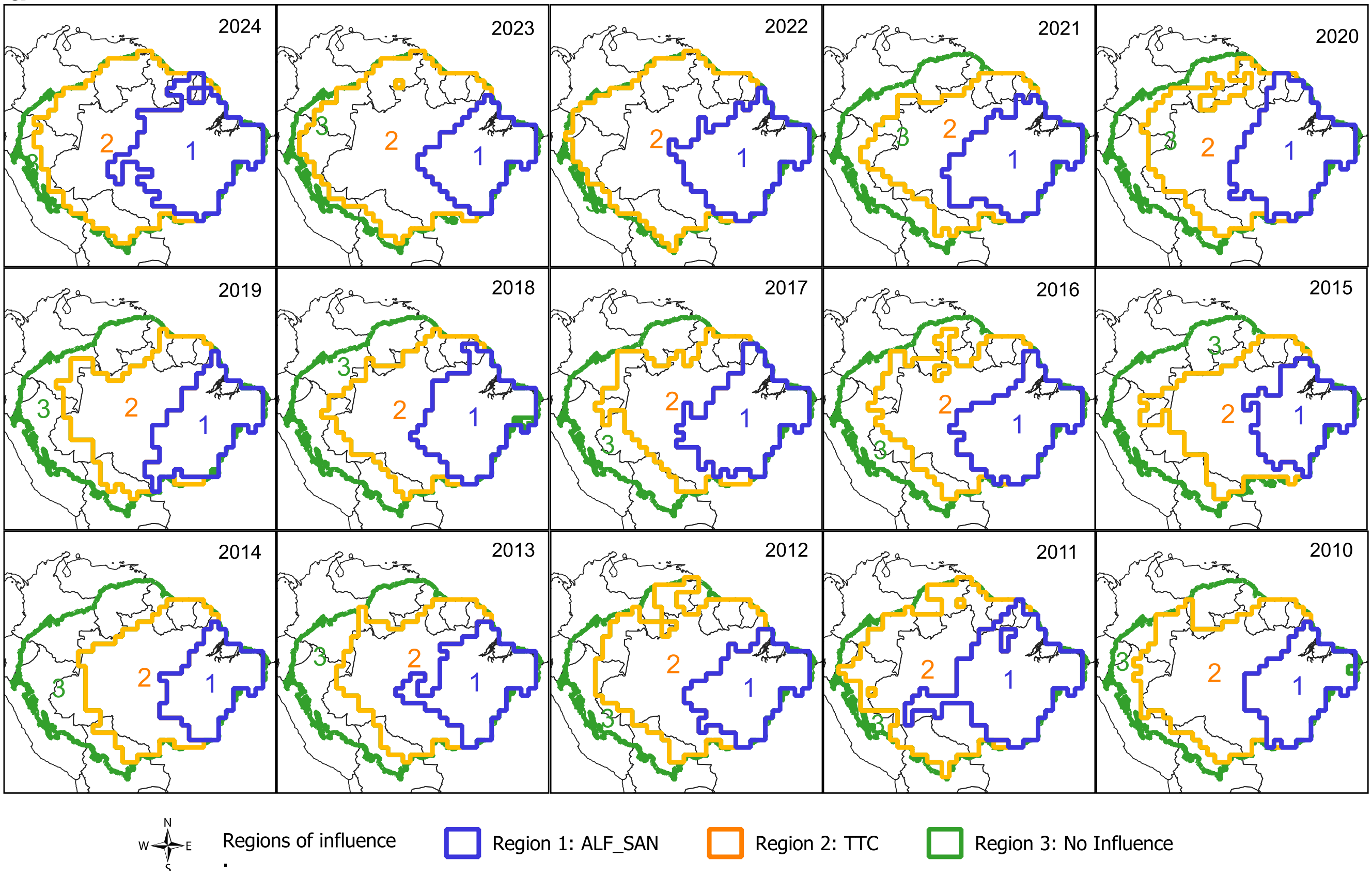

b

Years	Joining CARBAM areas (km²)								Total Amazonia Region	% of area not covered
	Region 1	Region 2						Region 3		
	ALF+SAN	RBA+TAB	RBA+TEF	RBA+CZS	(RBA+TAB)-(ALF+SAN)	(RBA+TEF)-(ALF+SAN)	(RBA+CZS)-(ALF+SAN)	Total Area - (Region 1 + Region 2)		
2010	1,743,337	5,316,368			3,573,030			1,384,430	6,700,798	21%
2011	2,592,327	6,136,448			3,544,121			564,350	6,700,798	8%
2012	1,779,830	5,355,634			3,575,804			1,345,164	6,700,798	20%
2013	1,855,531		5,143,327			3,287,796		1,557,471	6,700,798	23%
2014	1,498,921		4,614,307			3,115,386		2,086,491	6,700,798	31%
2015	1,623,597		4,883,233			3,259,636		1,817,565	6,700,798	27%
2016	2,049,203		5,474,447			3,425,244		1,226,351	6,700,798	18%
2017	2,113,210		5,183,361			3,070,152		1,517,437	6,700,798	23%
2018	2,101,036		5,230,053			3,129,017		1,470,745	6,700,798	22%
2019	1,790,849		4,995,610			3,204,760		1,705,188	6,700,798	25%
2020	2,383,976		5,781,567			3,397,591		919,231	6,700,798	14%
2021	2,271,149		5,340,159			3,069,010		1,360,639	6,700,798	20%
2022	2,198,314			6,676,750			4,478,436	24,048	6,700,798	0%
2023	1,708,379			6,420,179			4,711,799	280,619	6,700,798	4%
2024	2,581,225			6,442,170			3,860,946	258,628	6,700,798	4%

Supplementary Fig. 5 | Amazon subdivided into three regions. a, Subdivision of Amazonia into three regions from 2010 to 2024 was used to calculate Amazon's annual carbon balance and its annual mean vertical profile. Region 1 comprises the integration of the two eastern sites (SAN and ALF), which have the highest carbon emissions and the highest deforestation (35%). Region 2 comprises the integration of the two western-central sites (RBA and TTC, with less than 20% deforestation), with the area of Region 1 subtracted. Region 3 corresponds to the remaining area, not covered by the vertical profile's regions of influence. TTC corresponds to TAB (2010-12), TEF (2013-21), and CZS (2022-24). **b**, table presenting the three subregions from 2010 to 2024. ALF+SAN (Region 1), Region 2 [(RBA and TTC)-(SAN+ALF)], and Region 3, see Methods.
