# Supplementary material for: Amazon carbon emissions driven by human actions amplified by droughts
Source: Nature. 2026 Oct 21;658(8138):1188–93. doi: 10.1038/s41586-026-11168-1 (PMC13716835; doi:10.1038/s41586-026-11168-1)
Supplement: Supplementary file 2 [file 41586_2026_11168_MOESM2_ESM.pdf]

nature portfolio

Peer Review File

Amazon carbon emissions driven by human actions amplified by droughts

Corresponding Author: Dr Luciana Gatti

Any redactions in this file are there to maintain patient confidentiality, the confidentiality of unpublished data, or to remove third-party material.

This file contains all reviewer reports in order by version, followed by all author rebuttals in order by version.

Version 0:

Reviewer comments:

Referee #1

(Remarks to the Author)
Review of Gatti et al., Amazon carbon uptake is collapsing and shifting from sink to a source.

This is a very relevant and timely paper addressing key issues about the forcing of the Amazonian carbon balance through both anthropogenic effects such as deforestation and resulting climate impact through dry season lengthening and temperatures. If properly executed it would make significant contribution to our understanding of the vulnerability of the Amazonian carbon balance.

Major comment.

The profile method used here is a well established technique and resulting carbon flux estimates are well quantified and statistically well and soundly treated. This is unfortunately less the case in the grouping of the data, particularly the years on which the bulk of the conclusions rest. There is no statistical treatment of the selection of these yearly groups, some contain many years, other only one which makes the paper quite suggestive, but not robust. It would help enormously is the authors would spend more time on the selection, the underlying statistics, quantification of errors and the resulting correlation (or causal structure) of the interpretation. This is crucial to making the claims of the impact of climate versus deforestation in the paper.

Other comments

L32. Insert remaining before forest
L44. Do you mean resulting from shifts in climatic conditions
L53. Against which benchmark (year) was this calculated
L62. It would help the reader if you could add a few lines here telling the reader what you are going do in the remainder of the people
L77. Anthropic, you mean anthropogenic?
L92-l109. See major comment. Some form of error banding around the profiles would help.
L148-159. See major comment. This section requires statistical analysis to substantiate the rather strong claim in line 159-160. Now it is suggestive at best. One to one plots of anomalies plus statistics would also be nice. It is very difficult to make sense of figure 3.
L187. The fire emissions were
L212. Caption drivers in stead of drives
L233 presenting a reduction

Suppl. Figure 1. It would be good to include deforestation in this graph.

Han Dolman

Referee #2

(Remarks to the Author)
This is a comprehensive long-term study making use of 15 years of data from atmospheric profiles representing four key footprints in the Amazon Basin to assess interannual variation in the carbon balance. The data were divided into groups that related to climate conditions for years and policy drivers. They find that the Amazon shifted from a C sink to a source and show fire and anthropogenic pressure as dominant drivers. During La Nina the Amazon acted as a sink. 2024 was the worst year of emissions in their long record and was associated with El Nino. This is a robust study that builds on established past work by the team.

While the supplemental material provides more details by regions, the main text relies heavily on basin-wide temporal grouping. Given the heterogeneity of the basin and data, the main manuscript would benefit by including some of the spatial details, especially to support the policy vs climate distinction.

Consider that fire is more than simple emissions. It restructures the vegetation and has decade long impacts on above and below ground carbon. Fire converts rapidly decomposable carbon into recalcitrant black carbon (charcoal) with residence times of decades to millennia that will have long-term impacts on the carbon cycle; the total ecosystem loss might be different from what the atmospheric perspective implies. When you talk about record emission from fire, it would be interesting to know if those are coming from deforestation fires or fires associated with edges/degradation. The second is more challenging to deal with from a policy/management perspective.

Furthermore, there is limited field data available from other studies to understand how fire regimes vary with climate and degree of degradation, and how that affects smoldering combustion that affects CO ratios. Deforestation fires (flaming combustion) will have lower CO:CO2 than understory fires (smoldering). How could those interactions affect your fire emissions estimates and interpretation of the results. Using average ratios from across many years introduces uncertainty during anomalous fire years.

Other comments

Title
The wording of the title and some wording in the intro give the impression of a physiological response but the paper is framed in terms of land-use change. Consider changing the title to more clearly align with the work.

Abstract
--Line 30, "with fires" can mean a lot of things. Understory and fires in degraded forests are very different from fire from deforestation. Can you provide more detail here?

Intro

--what do you mean by overwhelmed and collapsed?
Lines 92-109: This text is dense. Perhaps summarise the group characteristics in a table to improve readability.
Lines 155: "Fire became a widespread deforestation tool following the reinstatement of public policies to curb deforestation in 2023." Reference required, or elaborate more.
Lines 100 Group 3: "absence of climatic stress", but note that "intense human activity" causes edge effects that are similar to drought effects and these effects extend deep into the forests. Consider "meteorological drought" instead.
Line105 Group 4: The strong wet-season sink, compensating for later losses, is interesting and could be explored more.
Line 153: "conditions that substantially increased forest flammability", while this is likely, was there evidence to show this? Later, at L162 you return to this topic and the statements could be more clearly linked.
Line 159: "These findings underscore the dominant role of human activity in driving year-to-year changes in Amazonian carbon fluxes". The link between human activity and the El Nino impact needs to be explicit, for example as additional ignition sources? Fragmentation reducing resilience?

--For the strong 2023 wet season sink and its potential interactions with La Nina, can you elaborate more on ecological mechanisms, e.g., access to groundwater (and associated literature). It looks like 2024 was not just due to a fire event, but the loss of the soil water buffer supporting physiological processes.

Figure1: Include units on the figure.
Figures throughout are of poor resolution and blurry, with the y-axes on Fig 3 illegible.
Figure 3: Are these colours colour-blind friendly?
Figure 4: Precipitation is presented, when it is fairly standard practice to use metrics like MCWD, as this provides a stronger indicator of water deficits experienced by vegetation, potential tree mortality, and as a potential metric of fuel dryness and fire risk.

The references provide appropriate credit to relevant work, but at times statements are made earlier in the text without reference (e.g., interpretation of the results) and a related statement made later in the text with a series of appropriate references.

Methods
--Could you provide more information how fire emissions were calculated. Emission factors vary substantially between agricultural areas, understory fires, and burning for deforestation. How was this variation addressed in your statistical approach and when quantifying uncertainty?
--The analysis uses deforestation and degradation data from PRODES and DETER. Estimating degradation for high biomass closed canopy forests is notoriously difficult for Amazonia, and it would be helpful to consider this uncertainty in estimates. Degradation is more than fire. Explicitly state whether the flux signal includes non-fire emission associated with degradation or are those included in the NBE or fire flux?
--The approach for group uncertainties, where group sizes were different, appears appropriate.

Referee #3

(Remarks to the Author)
Please note, that we reference line numbering as in the word document, not the PDF.

1. Summary of the key results
This work presents an extensive data set of 15 years of consecutive measurements of the carbon balance of the Amazon derived from the CARBAM measurement campaign, where Vertical Profiles (VP) of atmospheric CO2 content where collected across sites in the Amazon region. The data indicates that the Amazon is a carbon source in the majority of years within the study period mainly caused by years of extreme drought, high deforestation rates and/or fire activities which correspond to years with the highest deltaVP (positive CO2 balance, i.e. CO2 release to the atmosphere).

2. Originality and significance: if not novel, please include reference
The data set presented is extensive and consists of long-term observations of the severe anthropogenic impacts in the Amazon, which is a region of (global) significance as its huge carbon reservoir is important to stabilize continental and global climate, apart from being an important region rich in biodiversity and biocultural diversity. The 15-year time span covers different climatic extremes that occurred in the region, but also drastic changes in deforestation rates (all-time high and low rates) and fire activity which are strongly influenced by institutional and jurisdictional governance in Brazil. The measured CO2 profiles, together with multiple available remote sensing data sets, covering different drivers of forest change, allow the authors to investigate the impacts of these changing drivers to estimate the effect on Amazonia’s carbon balance (expressed as net biome productivity). To know if the entire Amazonia is a carbon source or sink as well as its trends is of high significance and interest to the wider readership.
However, we have some doubts if the manuscript has originality in a sense that similar results have been published before. We believe that the evaluation of this data set in its entirety (15 years), including the recent period 2018-2024 is novel, while parts of the same data set (the CARBAM campaign) have been published before with similar reasoning and argumentation of the drivers (deforestation, fire, dry periods) (Gatti et al. 2021, 2023). Now we want to bring the fact to your attention that the Methods section of this manuscript is almost identical to the one published in Gatti et al. 2023. We understand that the authors in Gatti et al., 2023 focused on a different aspect in the interpretation of the data (enforcement of deforestation laws on the carbon balance) and the topical overlap with this study is due to the nature of the data and evaluation methods. However, we do take issue with the fact that large parts of the wording of the Methods section in this manuscript are a replication to those in Gatti et al., 2023. To demonstrate that entire sections of previous articles were not simply copy-pasted, we expect the authors to describe their established methods in their own words in a different way, referencing the original publication.

3. Data & methodology: validity of approach, quality of data, quality of presentation
The authors describe how individual data sets were processed. It is concisely written and often sufficient information is provided. However, what is largely missing is a thorough statistical analysis of the drivers of carbon loss. At a first glance, the analysis is merely done by visual inspection. However, the analysis approach is hidden in one sentence in line 483, where “influence maps as spatial weighting functions for all studied parameters to determine how each parameter influenced the carbon flux” is mentioned, referencing Gatti et al. 2021. Unfortunately, tracing the methods description of this analysis step of that publication is not very detailed, so the reader needs to go back to this publication and still does not understand how the weighting process was done and what the statistical measures are. Additionally, since a large part of the results section of this manuscript is about time-series analysis of the entire study region, one would expect that additional (statistical) analysis is conducted to compare time series, including uncertainty measures, are provided – which is not the case. This is our main criticism where we regard the quality of presentation as being not sufficient for a high-ranking journal. Further, the presentation quality of results can be substantially improved. Sentences and figure captions are often incomplete, the presentation of results in some figures can be improved, incl. aggregating data to improve readability of the legend. We also miss a development of a thorough storyline that is easy to follow for readers outside this field. The reader who might not be familiar with the HYSPLIT model and the concept of back trajectory density is left in the dark, as it is not further explained in the main text how the regions were defined from the station data and how changes in weather conditions, incl. wind, influence the extent of the 3-dimensional packages (Fig. 1). Once the authors talk about Vertical Profile regions, when describing outcome of the next analysis step (next figure) groups are named according to their colour which is confusing for the reader.
The description of results changes from providing changes in absolute values to percentages in one sentence. We regard the presentation in percentages as problematic because the impact of small percent changes can be huge for one variable, but acceptable for another. Sometimes, the conclusion drawn from a set of results is unrelated to what the data actually show (e.g. L.154-156). This is especially evident in the section “Amazon precipitation and temperature changes (L114-135)”: it

seems that different time periods are compared and conclusions are drawn based on that. The section reads confusing in parts for which we provide examples here:
The paragraph starting in L116 compares the updated dataset (1979-2024) to the previously published part (1979-2018). It is not clear what the conclusions of this paragraph are. The authors first follow a clear logic of explaining impacts during the "new" period 2018-2024 (L 115-L124). Starting in L125 they write about regional analysis, but then discuss a multitude of different time intervals, which is confusing. For example, in L126-128, we cannot follow the logic how an increase in temperature during 1997-2018 is explained by deforestation between 2018-2024: "During 2018-2024, this region lost 37,930 km2 (2.4%) of primary forest due to deforestation. From 1997 to 2018, annual mean temperature rose by 0.93 ± 0.13˚C and by 1.90 ± 0.18˚C during the dry-season peak."
In L130 and following we do not understand the logic either: How is 1979-2024 a six-year period (the latter two sentences in the paragraph below), and how does the observation from 1979-2018 (first sentence) connect to this? "From 1997 to 2018, annual precipitation exhibited a non-significant downward trend of 208 ± 167 mm, while dry-season peak precipitation fell by 34% (67 ± 23 mm). From 1979 to 2024, annual precipitation decreased by 304 ± 150 mm (12%), and during dry-season peak declined by 37% (77 ± 23 mm). Over the same six-year period, SAN lost 15,023 km2 (2.3%) of primary forest. ". To us, these sentences are inconsistent and confusing.
Regarding deforestation and degradation, different established data sets are used. However, for the inexperienced reader (who has not read the long history of Brazilian deforestation and degradation products), little explanation is provided on how they are related and where they differ (with, e.g., DETER deforestation data being the preliminary data set at monthly resolution of the annual consolidated PRODES deforestation estimation).
Regarding fire, it is not sufficiently explained how the fire carbon emission in form of CO_2 is estimated using burned area, identifiable plumes in the VP during the dry season and fire activity data. But this is used in estimating the net biome carbon balance (L401 ff), whereas burned area is displayed in the time series (Fig. 4) and the connection to the other part of the analysis is unclear. It remains also unclear, why EVI data indicating vegetation activity are described in the Methods section but seem not to be used in the estimation of the carbon balance. Furthermore, it is unclear why only fire is considered in the calculation of the carbon balance and not carbon loss due to deforestation. We understand that this is difficult to separate from background data in comparison to fire, where it is possible to separate fire CO emissions from other environmental CO sources, but it needs to be clearly defined and explained. When the net biome productivity as a measure of the net carbon sink or source of Amazonia only considers fire in the carbon balance, how can conclusions be drawn with respect to deforestation and forest degradation?
On the use of climate data: it is unclear why VPD is used from CRU TS data set while air temperature data were taken from ERA5. Shouldn't both climate variables be consistent, i.e. used from the same data set? Furthermore, if precipitation data were used from GPCP because ERA5 data are biased this also has to be explained.
The regional analysis is provided in the supplementary material, its outcome, however, is not sufficiently presented in the main text.
Figure captions:
Abbreviations are often not explained. Colour-coding of different years could be improved, years belonging to the same phase of deforestation-fire development could be aggregated in the legend to improve readability. Typing errors were copied over to figure captions, indicating not a very careful editing of the manuscript by the author team, see list below.
Fig 1 ED. The numbering a-h mentioned in the caption (also referred to in Fig. 1 main manuscript) is missing in the Extended Data pdf document (cut off?)
Fig. 2 and 3 are blurry and not readable in both the submitted Word and the pdf document. We had to ask for a readable version from the Editor.
Caption typo Fig. 4 "Fig. 4 | Annual Mean Carbon Balance and the drives." - drivers
Caption typo Extended Data Fig. 6 | RBA and ALF annual mean Carbon balance and the drives. - drivers
Caption typo Extended Data Fig. 7 | TTC and SAN annual mean Carbon balance and the drives - drivers
Extended Data Fig. 8 | Seasonal RBA, TTC, ALF and SAN C Fluxes and Drivers. - fluxes and drivers OR always starting in capital letters in the entire manuscript.
Extended Data Fig. 8: What is the grey area indicating, the uncertainty as explained in the Methods section? This needs to be explained in the caption and /or included in the legend.

4. Appropriate use of statistics and treatment of uncertainties
No statistical methods are applied to the synthesis of time series data (see point 3. above). The determination of the uncertainties of the carbon fluxes is described in the Methods section, but not clearly indicated in any figure or table in the manuscript (but see our comment about ED Fig. 8). Uncertainty is provided for precipitation and temperature data, but not explained in the text.
5. Conclusions: robustness, validity, reliability
Given the challenges and issues reported above, it remains open if the conclusions, although they are clearly written, are valid and reliable. This refers especially to the numbers mentioned (e.g. 8% reduction in precipitation, we could not find this number within the manuscript). The severe carbon loss in the Southeast region has not been mentioned explicitly before, yet the conclusion should not present new findings to the reader.
6. Suggested improvements: experiments, data for possible revision
Provide statistical analysis of how provided time series are related and how significant those relations are, especially with regard to fire and deforestation/degradation as a source of carbon loss. Revise calculation of carbon balance that includes carbon loss estimation of deforestation and forest degradation.
7. References: appropriate credit to previous work?
Own previous work is referenced appropriately, with the exception of the Methods section. As we explain above, the authors copy-pasted entire paragraphs from their 2023 paper, which is not acceptable, even though the methods are similar.
8. Clarity and context: lucidity of abstract/summary, appropriateness of abstract, introduction and conclusions
Here, we highlight a selection of repetitive sentences and ask to adjust the text accordingly.

Abstract:
L25-26: remove duplicate of sentence “Forest loss reduces...” and also fix the references (different references cited in the duplicate): “Forest loss reduces precipitation, increases temperatures, and lengthens the dry season, driving higher carbon emissions4,5,8,10–12. Forest loss reduces precipitation, increases temperatures, and lengthens the dry season, driving higher carbon emissions13–16. “
Introduction:
While the authors elaborate on climatic stress and the evolution of deforestation in the region, they do not explain a brief timeline regarding burned area during the time period of the investigation. As burned area is one of the key variables investigated related to deltaVP and a major source of CO2, we suggest the authors add corresponding information in the introduction, including the source of the burned area data (as PRODES is mentioned for deforestation alerts).
At the end of the introduction, usually a short sentence or paragraph informs the reader what to expect in the following sections. Example from Gatti et al., 2023: “We compared the mean Amazonian carbon balance over 9 years (2010–2018)4 with the subsequent 2 years (2019 and 2020). (...)” . Such information is missing in the present manuscript.
We ask the authors to revise their manuscript in detail considering the following points, to improve readability, consistency and writing style. This applies especially to the Methods section, but also the other parts of the manuscript. The current version of the manuscript implies that the text has not been carefully revised among the team of authors before submission. Because of the many grammatical inaccuracies, missing words, differences in writing of abbreviations (small letters in one sentence, capital letters in another), punctation and so on, we expect a large task of manuscript editing in addition to the improvements on the analysis and presentation of results. Here we provide some examples for illustration:
– Consistent spelling of acronyms, e.g. PRODES or Prodes (both are used); BKG or bkg for background,
– Methods section: Some sentences appear unfinished or are not clear regarding their context. Example (L425-426): “To calculate the uncertainty for each vertical profile flux, from 1000 iterations using Monte Carlo randomization error propagation.” Example (L512-513): “The where the accuracy is 93.5%60.”
– use of dashes and hyphens: choose one form and stick with it. “–“ (e.g. L68) or “-“ (e.g. L69) or “—” (e.g. L92)

Parts we were out of scope of our expertise:
In the Methods section, L440 and following, the authors refer to a publication (Botia et al.) that criticized the authors' approach in uncertainty treatment of gas concentration measurements. The authors respond to this in the present manuscript, but it is out of our scope to assess if this approach is valid, or if the correction suggested by Botia et al. should rather be applied.

References
1. Gatti, L.V., C.L. Cunha, L. Marani, H.L.G. Cassol, C.G. Messias, E. Arai, A.S. Denning, L.S. Soler, C. Almeida, A. Setzer, L.G. Domingues, L.S. Basso, J.B. Miller, M. Gloor, C.S.C. Correia, others, Increased Amazon carbon emissions mainly from decline in law enforcement. Nature, 2023. doi: 10.1038/s41586-023-06390-0.
2. Gatti, L.V., L.S. Basso, J.B. Miller, M. Gloor, L. Gatti Domingues, H.L.G. Cassol, G. Tejada, L.E.O.C. Aragão, C. Nobre, W. Peters, L. Marani, E. Arai, A.H. Sanches, S.M. Corrêa, L. Anderson, others, Amazonia as a carbon source linked to deforestation and climate change. Nature, 2021. doi: 10.1038/s41586-021-03629-6.

Referee #4

(Remarks to the Author)
I co-reviewed this manuscript with one of the reviewers who provided the listed reports.

Version 1:

Reviewer comments:

Referee #1

(Remarks to the Author)
Review of Gatti et al,

The authors have made a very thorough restructuring of the paper that handles all my previous comments very adequately, in particular the cluster analysis. The paper is now also better organised and easier to read. I must also complement the authors on Figure 4, which is a great summary.

I have a few minor remaining comments/suggestions:

L32 insert :an analysis” before 15-year.
L46 through sink processes. I would suggest replace by carbon sequestration processes.
L49 insert with before atmospheric and replace accounts in the next line by accounting.
L81 it is not well explained why you choose 2018 as a break point. Did you use breakpoint regression analysis?
L156 how do you define moderate and strong etc.?
L209 it is interesting that the net balance is statistically not different between group 2 and 3. Maybe mention this? In fact you may consider writing a few lines on the fact that the NBE is always close to zero through the years (Fig 4), hence the difference is in the deforestation/fires.

Figure 2. It is interesting that the profile of group 3 extends well in the troposphere and appears structurally different from the other two. Maybe a small comment would help the reader. Is this transport by fire induced enhanced deep convection for instance?

Fig 5 Extended data. You have the Portuguese spelling for the months (i.e. Fev instead of Feb). Please change.

Referee #2

(Remarks to the Author)
The authors have improved the revised manuscript. I appreciate their careful work to respond to questions about the previous version.

Overall, the authors have substantially addressed my original critiques. The additional info about spatial heterogeneity and policy vs climate was helpful. The additional deeper look into potential human vs lightning drivers of fire was also useful. More could have been stated about the distinction between deforestation fires (flaming with higher CO2 fraction) and degradation/understory fires (smoldering with higher CO fraction), although I am aware the authors are up against word limits. I agree with the authors that my comments about the strong 2023 wet season sink and its potential interactions with La Nina could be another extensive analysis and may not be possible to address within space limitations.

L153 stated that Group 3 comprises years characterised by “intense global climate events, such as El Nino and TNA warming”. However, it looks like your Welch’s ANOVA for ONI and TNA were non-significant compared to other groups (Suppl. Table 1).

For the intro, it would help to have some background information on your atmospheric measurements to support the first mention in the text “However, our direct atmospheric measurements…”
The conclusions could be strengthened to clarify the message and provide a clearer, forward-looking statement based on the results. Also, the second-to-last sentence referring to 2023/24 as a question is unclear.

Referee #3

(Remarks to the Author)
A. Summary of the key results
The study represents an extensive data set covering 15 years of consecutive air column CO2 and CO measurements which were derived from the CARBAM measurement campaign for four sites in the Amazon biome. Each site integrates carbon fluxes from a region of a different size which altogether represent changes in the carbon balance of the Brazilian Amazon. The author team analysed the carbon balance as the result of changes in its anthropogenic (fire, deforestation, degradation, land-use) and climatological drivers for individual years in the 15-year time period. They conducted a statistical analysis to classify individual years into groups of wet/dry years in combination with high/low anthropogenic activities that include setting fires, deforest and degrade tropical rainforest.
B. Originality and significance: if not novel, please include reference
We appreciate the authors’s response to our criticism regarding originality of the manuscript. We value the fact that by applying a statistical analysis that groups the individual years into 3 classes of wet/dry years, which can be characterized by ENSO events or Tropical North Atlantic Ocean influence, with low/high deforestation rates and fire occurrence. Especially, the additional work on lightning strikes and lightning-caused fires is novel and we highly value this additional analysis as it increases the originality of the manuscript. However, in some places the authors miss to take the final step, i.e. conclude the exercise with – in this example – a clear statement that the fire carbon emissions are human-caused and lightning-caused fires make up 5-10% of all fires. This also applies to the analysis of the other drivers (deforestation, degradation, land-use for agriculture and pasture, temperature increase and precipitation decrease) as well. Their changes are presented (with improved quality), but the reasoning that they are indeed explaining the measured total carbon fluxes is left to visual inspection. An example on how to interpret TCF, FCF and NBE, when TCF is positive and NBE is negative is important, see comment below.
The manuscript text still uses similar framing of previous publications (Gatti et al. 2021 and 2023) which is acceptable, but the baseline text of those previous publications is not revised in such a way that it increases readability of the text to a wide audience, i.e. the average Nature reader. Moreover, the analysis of deforestation, degradation and fire has been analysed for each individual region that the measurement sites cover in Tejada et al. (2023) and been compared against bottom-up carbon budgets, which analysed if individual years were either a source or sink of carbon. The study of a similar author team showed that large uncertainties can remain with respect to the identification of individual year’s carbon balance and that the source-sink distribution of years and/or the size of the source (sink) can differ between the top-down (this study approach) and bottom-up (Tejada et al. 2023). We suggest, instead of adding more and more analysis and expanding the current manuscript with even more extended data and supplementary text, that the author team focusses on the grouping of the individual years and explains
- the implication of lighting- vs. human-caused fires as well as deforestation and degradation on the total carbon flux in those three groups of years, i.e. translating the area loss into influence of measured carbon fluxes.
- the specific conditions that might have led to the carbon source in the 2023-24 El Niño event and the acceleration in droughts due to increased deforestation and degradation in the previous years.
by focussing on the translation of the changes in area to likely carbon fluxes (which is BTW explained better in the previous Gatti et al. publication, and can be referred to in this manuscript). The material is more or less there but needs the

quantification and description from area-based changes to the influence on the TCF and NBE in a concisely written text that it understandably written for a wide readership. Of course, explanatory text that is required to understand applied methods and context in this manuscript has to stay but should clearly reference previous publications. It would help to avoid giving the impression that too many things are repeated and confuse the current originality of the study. We provide examples in the next section C.

C. Data & methodology: validity of approach, quality of data, quality of presentation

We thank the author team for their response and how they explained the applied statistical methods (site vs. region of reference). Again, referencing to the Tejada et al. 2023 study would also be helpful for the reader to understand how process (changes) influence the measurements taken at the sites. Furthermore, we suggest to focus the regional analysis on the two points suggested in the previous section.

We understand that 3000 words set a limitation to explain the applied methods in an understandable way, the current solution is not helping either. In previous publications Gatti et al. did explain why EVI, VPD, burned area, deforestation etc. were used, namely in the statistical analysis. The text blocks in the methods sections have remained, but the central sentence similar to Gatti et al. 2023 is missing. Specifically, the use of EVI and VPD are not explained, not in the Methods section of the main paper, nor in the Supplementary Material section, even though it was mentioned in the response letter. We would ask the author team to add this important point. So, is it the same method to use all those variables in the imputation of the total carbon flux (TCF)? Please describe what exactly is meant by imputation.

VPD is used in the analysis, the title states that droughts are analysed, but VPD is not prominently described and discussed in the main text. When removing previously published text components, it should give space to discussing VPD changes in addition to precipitation and temperature.

We think it is important to describe the historic land-use changes (section from lines 65-77), incl. increases in pasture, soybean and mining areas. However, this detailed information only makes sense to be reported if it explains how pastures, soybean fields and mining have influenced the measured carbon fluxes in the air column. If this cannot be differentiated, it could be a way to remove this text and describe the influence of deforestation and degradation on the 3 groups, incl. fire, and specifically the El Niño event.

Quality of presentation. The quality of presentation has improved in many ways and we appreciate the detailed effort the author team put in this manuscript revision. We also acknowledge the effort put in to address detailed comments of every reviewer and still produce a consistent manuscript. Our suggestions for improvements aim at further increasing the manuscripts quality and ask the author team to implement the following:

1. Section Amazon climate changes:

a. The changes in precipitation and temperature for the two time periods compared in the text are not displayed in Extended Figure 1, the annual precipitation and dry-season precipitation should be plotted instead in two separate panels in this figure to substantiate the text from lines 82-87.

b. Unclear why deforestation numbers are mentioned here again. Please clarify the difference to the above section on historic land-use change.

2. Caption of Fig. 3:

a. To allow readers outside your field understand the meaning of the TCF, we suggest to label (and mention in the manuscript text) what negative and positive TCF numbers mean, even though one can deduct it from the methods and equations.

b. Panel c: We would suggest to colour the axis and labels in the colour of the precipitation graph to help visual reading of the line colour.

3. We suggest to report changes in VPD and EVI to report drought conditions in the individual years in addition to precipitation and temperature which display their changes, not absolute numbers, to indicate increasing drought conditions relative to average climate. We suggest to add this to Figure 3 and adapt respective descriptive text.

a. In the response letter the author team seem to suggest that MCWD was used but we could not find it in the text. We understand that it is important to have also a monthly metric for drought conditions. While the author team already uses VPD and EVI (among others) for gap filling, these variables could also be used to quantify drought conditions. In addition to precipitation the argument then still holds that deforestation and forest degradation starts to use precipitation. We suggest to clearly state this as an interpretation from the data rather than a proof arising from carbon fluxes shown here.

4. Figure 5:

a. Top line of data boxes displaying the bar plots of a number of drivers: The size of the bars displaying NBE, DEF and BA do not match. The NBE bar for group 2 is unreasonable large (value of 0.07) compared to group 3 (value of -0.06). For BA the bar in Group 3 should be roughly the double in size compared to BA in Group 1. Furthermore, the legend for these bars in the left-most box only mentions carbon flux units and misses the area-based units. Please check and revise as this figure might be widely used.

b. Legend: Please check index for precipitation to have capital letters. Precipitation should be marked as annual precipitation, Temperature as mean average temperature, or use abbreviations and explain them in the legend text.

5. The concluding general numbers for the Amazon region appear in the Conclusion section for the first time, we recommend to close Section "Grouping of years" with basin-wide numbers and reference corresponding data and figures (which do not include those number so far). Therefore, this section should describe and discuss Figure 5, which now appears in the conclusion section for the first time.

6. Even though it has been published in Nature before, the terms used here Total carbon flux, fire carbon and net biome exchange (NBE) remains confusing for reader outside your field. It remains unclear, how from equation M1 (for which the definition of variables and their units needs to be added) these fluxes can be deducted from the background (lines 389-393 do explain one bit, the main text (lines 119-124 another) and seem not to match. Please explain the difference in carbon balance then TCF is positive and NBE is negative, or different in quantity – how should these different numbers be interpreted? To take one example from Figure 5, Group 3 has a TCF of 0.49 PgC p.a., because FCF is 0.55 PgC p.a. the remaining other carbon fluxes (uptake and release) lead to a negative NBE (-0.06 PgC p.a.). So, TCF means it is 0.49 PgC higher than the background carbon flux, which points to large carbon emissions. The calculation of the fire carbon flux, using

equation M1, assigns a carbon flux of 0.55 PgC p.a. which exceeds TCF. What is the interpretation of the carbon budget for a negative NBE, does it mean that the ecosystem is reducing its carbon stocks? We think, explaining those examples clearly, helps to understand how the findings should be interpreted correctly. Further comments for this section are:
a. The description should focus on the different interpretations for the 3 groups and 2023-2024 years.
b. Description of figures 2 and 3 in the text fall short of the content that both figures show, i.e. do not use the text to list the variables shown, rather the describe the content. When the individual years show quite some variability can you conclude a trend as indicated in the other sections?
7. Description of Groups: Please check if all references to extended data figures and tables are necessary. The listed quantities refer to Extended Data Table 3 in all paragraphs. Which information is mentioned from the other display items and requires citations?
Detailed comments
1. Line 49: revise language to connect the text after semicolon to message of sentence.
2. Line 59: add references to statement on “extreme events as a reason for increased carbon emissions.”
3. Line 125 revise title of section to improve language and clarity
4. Line 145: numbers are not shown in Table 3b
5. Line 145-146: it is the mean TCF and mean delta VP which values are shown, please revise.
6. Line 149-150: please mark qualitative meaning of ONI and TNA values in Figure.
7. Line 151-152: statement not substantiated by display items, please add.
8. Line 154-155 Since ONI and TNA provide changes in temperatures I would suggest not to mention the difference in percentages
9. Figure 3c could profit from marking El Niño years with a symbol in figure panel
10. Line 159: add “(compare Extended Table 3a with 3b)” after Group 1.
11. Line 164, add reference to Extended Table 3c
12. Line 166: Mentioning Data Fig 7 is not needed since monthly changes are not mentioned in this sentence. Add “Mean” to “Fire carbon flux”
13. Extended Figure 7: please mark wet-season months in figure panel or delineate them using stippled lines
14. Line 194: do you mean people used fire to degrade the forest to then be able to deforest?
15. Line 196: the section should close with a summary of biome-scale and time-average numbers. The reference to detailed subregional analysis should be moved where regional differences are discussed (and be included only when necessary for understanding the main story).
16. Line 362: spell out abbreviations of sites when first mentioned.
D. Appropriate use of statistics and treatment of uncertainties
We thank the author team for their efforts to improve the use of statistics in this manuscript. We suggest to use the regional analysis only to explain regionally different impacts on 2023 and 2024 El Niño drought year.
As mentioned under point A, uncertainties in estimating the years’ carbon balance (source or sink year) and the size of the source or sink depending on the top-down (this study) or bottom-up (Tejada et al. 2023) to be included in the discussion of this study. Readers would possibly interpret the findings of this study as a given...
E. Conclusions: robustness, validity, reliability
The Conclusion section has very much improved. It now substantiates better the findings of this manuscript. With the suggested updates in the sections A and B, we think the Conclusions are robust.
There are a few things we recommend to make it consistent and valid:
The Statement in Line 200 is fine if there is a biome-wide summary in the corresponding sections above.
Line 204-205: this information was not provided, please mention in text on climatic influence to be able to refer to these changes in conclusion
Line 206: to which time period do the temperature changes refer?
Line 215-216: you should be able to provide an answer to this question on how the 2023-24 El Niño event influenced TCF and NBE.
F. Suggested improvements: experiments, data for possible revision
Our concerns raised in the first review were addressed.
G. References: appropriate credit to previous work?
Yes, can be improved wrt to previous findings of author team. Citing Tejada et al. to refer to the regional pattern the site measurements capture important information and can help to make the new measurements and knowledge provided with this study update more concise.
H. Clarity and context: lucidity of abstract/summary, appropriateness of abstract, introduction and conclusions
Our concerns regarding Abstract, introduction and Conclusion were addressed appropriately.
References
1. Gatti, L.V., C.L. Cunha, L. Marani, H.L.G. Cassol, C.G. Messias, E. Arai, A.S. Denning, L.S. Soler, C. Almeida, A. Setzer, L.G. Domingues, L.S. Basso, J.B. Miller, M. Gloor, C.S.C. Correia, others, Increased Amazon carbon emissions mainly from decline in law enforcement. Nature, 2023. doi: 10.1038/s41586-023-06390-0.
2. Gatti, L.V., L.S. Basso, J.B. Miller, M. Gloor, L. Gatti Domingues, H.L.G. Cassol, G. Tejada, L.E.O.C. Aragão, C. Nobre, W. Peters, L. Marani, E. Arai, A.H. Sanches, S.M. Corrêa, L. Anderson, others, Amazonia as a carbon source linked to deforestation and climate change. Nature, 2021. doi: 10.1038/s41586-021-03629-6.
3. Tejada G, Gatti LV, Basso LS, Cassol HLG, Silva-Junior CHL, Mataveli G, Marani L, Arai E, Gloor M, Miller JB, Cunha CL, Domingues LG, Ipia A, Correia CSC, Crispim SP, Neves RAL and Von Randow C (2023) CO2 emissions in the Amazon: are bottom-up estimates from land use and cover datasets consistent with top-down estimates based on atmospheric measurements? Front. For. Glob. Change 6:1107580. doi: 10.3389/ffgc.2023.1107580

Referee #4

(Remarks to the Author)
I co-reviewed this manuscript with one of the reviewers who provided the listed reports.

Open Access This Peer Review File is licensed under a Creative Commons Attribution 4.0 International License, which permits use, sharing, adaptation, distribution and reproduction in any medium or format, as long as you give appropriate credit to the original author(s) and the source, provide a link to the Creative Commons license, and indicate if changes were made.
In cases where reviewers are anonymous, credit should be given to 'Anonymous Referee' and the source.
The images or other third party material in this Peer Review File are included in the article's Creative Commons license, unless indicated otherwise in a credit line to the material. If material is not included in the article's Creative Commons license and your intended use is not permitted by statutory regulation or exceeds the permitted use, you will need to obtain permission directly from the copyright holder.
To view a copy of this license, visit https://creativecommons.org/licenses/by/4.0/

São José dos Campos, 14 May 2026

Response to Nature Editor and the Reviewers Dr Han Dolman, Reviewers 2, 3, and 4, about the submitted paper Nature manuscript 2025-11-29496

Thank you for all the suggestions, comments, and critical reviews that helped produce a better, clearer manuscript. We recognise that many suggestions are making the article stronger, with clear arguments. Please see our answer and the description of all modifications we have made in the manuscript.

We would like to thank everyone for their suggestions and criticisms, which enriched our article and helped the scientific community and society better understand it.
We would like to apologise for the time it took to produce this second version; however, the changes introduced by the statistical analysis led to the restructuring of much of the content, figures, and tables. But we believe the result was much better, and for that, we greatly appreciate the suggestions for change.
The main suggestion that led to a major change in the article was to use statistics to justify separating the groups by year. Using statistical methods, as detailed in this response and in the article itself, we obtained 3 groups of years rather than the 5 groups in the first version of the article. We revised the paper's structure to make it clearer. At the end of the Introduction, we add the main objective:
Lines 60-64: "Here, we use our 15-year history of Amazonian carbon fluxes derived from atmospheric CO2 measurements to answer important questions: What are the main factors driving increased carbon emissions and variability in the Amazon? Are they caused by global extreme climate events or direct human activities (deforestation, biomass burning and degradation) in the Amazon?"
Then we change the paper structure: first, a description and analysis of deforestation, separated by a specific subheading, followed by changes in precipitation and temperature resulting from deforestation. We presented all results from annual mean vertical profiles and carbon fluxes, as well as the climatic and anthropogenic parameters. After all results, we introduced the statistical analysis, presenting and analysing the three groups to understand the impacts of each driver.

Since the changes were very large, we will submit 2 versions: 1 - the new version with all changes, with track changes activated. 2- the revised version with accepted changes to get the correct lines and pages for the answer comments (Gatti_et_al_revised version_without track changes.doc). The version that shows all changes makes the line and page counts incorrect, since it includes the deleted part of the text.

The statistical method used to determine the number of groups/years was K-means, applied in the PCA space. The classification of **Group 1** remained exactly the same, with the same years (2011, 2012, 2013, 2014, 2017 and 2018) and the same characteristics: group of rainy years and low human activity, characterised by deforestation and fires.

The current **Group 2** was classified as Group 3 in the previous version: the group characterised by a large increase in deforestation and low climate stress (2019, 2020, 2021, and 2022).

The current **Group 3** is the combination of groups 2 (2010, 2015, and 2016), 4 (2023), and 5 (2024), in the first version, characterised as years of extreme El Niño and TNA events. Interestingly, the division remained exactly as we had done previously; however, in the previous version, we separated the years 2023 and 2024 because they were very exceptional years (2023 showing the highest carbon absorption during the wet season, and 2024 the highest carbon emission, with the largest burned area, since the beginning of our study in 2010). However, with the statistical basis showing group separation, we believe the grouping of years is more scientifically grounded. Because, in the characteristics of Group 3, as determined by statistics, the years were characterised by El Niño and TNA climatic extremes, accompanied by a significant increase in biomass burning. It is now clear that increased biomass burning is also a key characteristic of this group. And so, it was necessary to add a study about lightning-induced fires. Was this increase natural or anthropogenic?

We added a paragraph at the end of the introduction that explains this main objective, pointing out the use of 15 years to answer the question of the strongest influence in Amazonia: global climate change or anthropogenic activities, such as deforestation and biomass burning. and the article was practically rewritten and restructured, now having only 3 groups. Almost all the figures were updated and simplified in line with your suggestions.

We believe that the changes made the study more robust and clearer. We are very grateful for these suggestions, which truly improved the article. Thank you very much.

Because we added the fire-lightning study, we added 2 more authors: Vanucia Schumacher and Kleber Naccarato. We also added John B. Miller and 2 other colleagues for NOAA: Xin Lan and Gaby Patron. John Miller has been an important part of this Project since 2004, participating in the construction of this network. The first submission was made during a prohibition imposed by the Trump administration and during NOAA's closed period.

To organise the corrections, we have made to the text, we will repeat the request and the changes made point to point:

Answering a point-by-point response to our referees and to any editorial suggestions

Referees' comments:

Referee #1 (Remarks to the Author):

Dear Han Dolman

We would like to thank you for your suggestions, which led to a qualitative improvement in the paper and helped the scientific community and society better understand it.

The main suggestion that led to a major change in the article was to use statistics to justify separating the groups by year. Using statistical methods, as detailed in this response and in the

article itself, we obtained 3 groups of years rather than the 5 groups in the first version of the article.

The statistical method used to determine the number of groups/years was K-means, applied in the PCA space. The classification of **Group 1** remained exactly the same, with the same years (2011, 2012, 2013, 2014, 2017 and 2018) and the same characteristics: group of rainy years and low human activity, characterised by deforestation and fires.
The current **Group 2** was classified as Group 3 in the previous version: the group characterised by a large increase in deforestation and low climate stress (2019, 2020, 2021, and 2022).
The current **Group 3** is the combination of groups 2 (2010, 2015, and 2016), 4 (2023), and 5 (2024), in the first version, characterised as years of extreme El Niño and TNA events. Interestingly, the division remained exactly as we had done previously; however, in the previous version, we separated the years 2023 and 2024 because they were very exceptional years (2023 showing the highest carbon absorption during the wet season, and 2024 the highest carbon emission, with the largest burned area, since the beginning of our study in 2010). However, with the statistical basis showing group separation, we believe the grouping of years is more scientifically grounded. Because, in the characteristics of Group 3, as determined by statistics, the years were characterised by El Niño and TNA climatic extremes, accompanied by a significant increase in biomass burning. It is now clear that increased biomass burning is also a key characteristic of this group. And so, it was necessary to add a study about lightning-induced fires. Was this increase natural or anthropogenic?

Review of Gatti et al., Amazon carbon uptake is collapsing and shifting from sink to a source.

This is a very relevant and timely paper addressing key issues about the forcing of the Amazonian carbon balance through both anthropogenic effects such as deforestation and resulting climate impact through dry season lengthening and temperatures. If properly executed it would make significant contribution to our understanding of the vulnerability of the Amazonian carbon balance.

Major comment.

The profile method used here is a well established technique and resulting carbon flux estimates are well quantified and statistically well and soundly treated. '\'

Other comments

L32. Insert remaining before forest
Authors' answer:
Thank you for the suggestion: included. Line 32 in this version is now line 28.

L44. Do you mean resulting from shifts in climatic conditions

Authors' answer:
Thank you again for the suggestion. The phrase was not clear. To become clearer:

we replaced "…and to shifts in climatic condition" to "…and changes in regional climatic conditions modified by deforestation"

L53. Against which benchmark (year) was this calculated

Authors' answer:
PRODES began in 1988, but this year, the total deforested area in the Amazon was calculated. We introduced 3 modifications in this part:
We included the description since 2000 to harmonise the time series lengths for deforestation, soybean plantations, pasture, and mining.
The topic Historic Land Use Change in Amazonia is from line 65 to 77.

L62. It would help the reader if you could add a few lines here telling the reader what you are going do in the remainder of the people

Authors' answer:
We agree completely with you. We add the paper's objective explanation to the final introduction (lines 60-64). Now the paper structure is different to be clearer to the reader. First, we introduced the main objective. Then we analysed historical deforestation and the consequences for precipitation and temperature. In the sequence, we presented all results from annual mean vertical profiles and carbon balance. We add the important parameters that drive the variability in carbon fluxes for each year. After that, we presented the statistical analysis and the resulting groups. The analysis of the groups identifies the main drivers and answers the main question.

L77. Anthropic, you mean anthropogenic?

Authors' answer:
Thank you for pointing out this mistake. We substitute the term in lines 109, 115, and 121.

L92-l109. See major comment. Some form of error banding around the profiles would help.

Authors' answer:
Thank you for the suggestion. The updated Figure 2c (3 groups) now shows bars representing the standard deviation of the year's mean inside each group. Now we also add the Ext. Data Table 2 presents all annual data and results from 2010 to 2024 for all parameters studied. The topic (Annual mean vertical profiles) is now on lines 100-115.

L148-159. See major comment. This section requires statistical analysis to substantiate the rather strong claim in line 159-160. Now it is suggestive at best. One to one plots of anomalies plus statistics would also be nice. It is very difficult to make sense of figure 3.

Authors' answer:
Again, many thanks for the suggestion, which led to a significant improvement in the paper.

We also changed Figures 3 and 4, which were very complex. The initial idea was to share all information. But now the idea is to show in the figures only the information relevant for the interpretation and conclusions. The remaining information is presented in Extended Data Tables 2 and 3. The statistical study was included in the text, and all group descriptions and interpretations are presented from lines 125 to 179. Statistics are also described/presented in Methods, Extended Data Fig 4, Supplementary Discussion 2 and Supplementary Table 1.

L187. The fire emissions were

Authors' answer:
Thank you again. Very well pointed. Corrected (now line 140)

L212. Caption drivers in stead of drives

Authors' answer:

The Figure was updated (from before Figure 4 to Figure 3), and the term was corrected. Thank you again.

L233 presenting a reduction

Authors' answer:

This recommendation was completely rewritten to reduce the text, and it now spans lines 212-217. I did a more direct recommendation in this current version.

Suppl. Figure 1. It would be good to include deforestation in this graph.

Authors' answer: Thank you again. We also included deforestation and mining in the Suppl. Fig. 1. In the current version, the text and figures are from the same period: from 2000 to 2024.

Han Dolman

Referee #2 (Remarks to the Author):

This is a comprehensive long-term study making use of 15 years of data from atmospheric profiles representing four key footprints in the Amazon Basin to assess interannual variation in the carbon balance. The data were divided into groups that related to climate conditions for years and policy drivers. They find that the Amazon shifted from a C sink to a source and show fire and anthropogenic pressure as dominant drivers. During La Nina the Amazon acted as a sink. 2024 was the worst year of emissions in their long record and was associated with El Nino. This is a robust study that builds on established past work by the team.

While the supplemental material provides more details by regions, the main text relies heavily on basin-wide temporal grouping. Given the heterogeneity of the basin and data, the main

manuscript would benefit by including some of the spatial details, especially to support the policy vs climate distinction.

Dear Referee #2

We would like to thank you for your suggestions, which led to a qualitative improvement in the paper and helped the scientific community and society better understand it.

Authors' answer:

We agree completely, thank you for the comment, but the problem is the paper's size limit. This is why the regional analyses are presented in Supplementary Discussion 3 (2 pages) and many Figures. In some sections, we add a bit of discussion, as presented in the section on Annual precipitation and temperature changes (from line 92 to 99). In line 195, we inform readers that the detailed discussion of the region is in Suppl Discussion 3, Extended Data Tables 1 and 2, Extended Data Figs. 2 and 3, and Supplementary Figs. 3, 4, and 5.

Consider that fire is more than simple emissions. It restructures the vegetation and has decade long impacts on above and below ground carbon. Fire converts rapidly decomposable carbon into recalcitrant black carbon (charcoal) with residence times of decades to millennia that will have long-term impacts on the carbon cycle; the total ecosystem loss might be different from what the atmospheric perspective implies. When you talk about record emission from fire, it would be interesting to know if those are coming from deforestation fires or fires associated with edges/degradation. The second is more challenging to deal with from a policy/management perspective.
Furthermore, there is limited field data available from other studies to understand how fire regimes vary with climate and degree of degradation, and how that affects smoldering combustion that affects CO ratios. Deforestation fires (flaming combustion) will have lower CO:CO2 than understory fires (smoldering). How could those interactions affect your fire emissions estimates and interpretation of the results. Using average ratios from across many years introduces uncertainty during anomalous fire years.

Authors' answer:
Thanks for the comment. In this restructured text and discussion, we sought to address the cumulative effects of deforestation and biomass burning on regional/subregional climatic conditions that affect forest function. The change in vegetation after the fire was not explored.
We are writing another paper focused on the fire emissions. We agree about this uncertainty. In this paper, we attempted to use a monthly CO/CO2 ratio per site to reduce one source of uncertainty, but the results became less correlated with fire spots. Then we decided to explore the variability between months and years in depth in the next paper. We have 15 years, more than 1100 vertical profiles. It will be an important contribution to explore both variabilities further. Over time, as Amazonia becomes drier and hotter, the ratios are consistently lower every time we add more VP, with new ratios CO/CO2. In this paper, we calculated the mean ratios again and presented them in the Methods.
We add to the Methods section, this source of uncertainty, from lines 447 to 451

“An additional source of uncertainty is addressed in the calculation of fire carbon emissions and NBE. Over time, the forest is becoming drier and hotter. These changes promote a lower CO/CO2 ratio. The use of a single mean CO/CO2 ratio is another source of uncertainty, leading to lower fire emissions than those observed in recent years because the ratio tends to decline over time. It is likely that carbon emissions from fires were higher than our averages for 2023 and 2024 indicate, because both were drought years."
Many thanks again for the suggestions.

Other comments

Title
The wording of the title and some wording in the intro give the impression of a physiological response but the paper is framed in terms of land-use change. Consider changing the title to more clearly align with the work.

Authors’ answer:
Thanks for the comment. We agree and changed the title to: “***Direct human actions, amplified by droughts, dominate Amazon C emissions***”. We believe this new title is more related to the main scientific conclusions.

Abstract
--Line 30, “with fires” can mean a lot of things. Understory and fires in degraded forests are very different from fire from deforestation. Can you provide more detail here?

Authors’ answer:
Thank you for your comment, and you are absolutely right. The initial problem is that we identify the fire based on CO concentrations in the atmosphere. We have no way of identifying its origin, and the abstract has an enormous word limit. The recommended length is 200 words, and we're already at 230 (the maximum allowed in exceptional cases, according to the publisher).

Intro

--what do you mean by overwhelmed and collapsed?

Authors’ answer:
The editor instructed us to move a portion of the Introduction into the Abstract and to avoid repetition in the Introduction. We have rephrased this section and hope the message is clearer. These issues are addressed in lines 26-27 of the abstract and lines 44-47 of the introduction.

Lines 92-109: This text is dense. Perhaps summarise the group characteristics in a table to improve readability.

Authors’ answer:
Thank you for the suggestion. After introducing statistical analysis to define year groupings, we now have 3 distinct year groups. The old groups 1 and 3 continue the same. Groups 1, the wet

years with low human activities, such as deforestation and degradation; and group 2, marked by strong human activities (deforestation mainly) and low climatic stress (the old group 3). The current Group 3 is the integration of the old groups 2,4, and 5. Now it is much more consistent, because the main characteristics are extreme climatic events that are associated with increased biomass burning, highlighting the important association between droughts and the amplification of burning areas. With this strong association, it was necessary to include the study to identify the origin of fires: whether natural or anthropogenic. We also included this study, which showed 98% of fires are anthropogenically induced. In the current version, we have added the Figure resume (Fig. 5) and the Extended Data Table 3.

The new discussion, in this current version, about the groups integrates the results for the annual mean vertical profile and the annual C flux. From lines 126 to 179.

Lines 155: “Fire became a widespread deforestation tool following the reinstatement of public policies to curb deforestation in 2023.” Reference required, or elaborate more.

Authors’ answer:
Thank you for the suggestion. This discussion is now on the lines from 194 to 195, now with references. PRODES identified 38% of deforestation in 2024 was caused by fire (reference 36).

Lines 100 Group 3: “absence of climatic stress”, but note that “intense human activity” causes edge effects that are similar to drought effects and these effects extend deep into the forests. Consider “meteorological drought” instead.

Authors’ answer:
Thank you for the suggestion. In the current version, we changed to “Group 1, representing the wettest years with low levels of human disturbance (deforestation and degradation), shows a near-neutral annual mean Total Carbon Flux (from line 157 to 160).

Line105 Group 4: The strong wet-season sink, compensating for later losses, is interesting and could be explored more.

Authors’ answer:
Thank you for the suggestion. After the introduction of the statistical study and the number of groups increased to 3, this discussion was moved to a new subheading titled: The exceptional years 2023 and 2024, because these years are part of the current group 3.

The current paragraph is: The 2023 wet season exhibited the largest carbon sink over our 15-years study period (Fig. 4 and Extended Data Fig. 7), which occurred after three consecutive La Niña years (Fig. 4). The 2023 Amazonia carbon balance was similar to the Group 1 (0.11 ± 0.09 PgC y-1), because the wet-season sink offset 75% of the carbon emissions from deforestation and burned area, which were, respectively, 38% and 52% higher than in Group 1

Line 153: “conditions that substantially increased forest flammability”, while this is likely, was

there evidence to show this? Later, at L162 you return to this topic and the statements could be more clearly linked.

Authors' answer:
Thank you for the suggestion. The current Group 3 integrated all drought and warming years, and it became clear that biomass burning increased, indicating an association between the two. This discussion is now presented between lines 181 and 196.

The 2024 discussion is now in the lines from 213 to 219, and text now is: "While 2023 began after 3 years of La Niña, 2024 began in the middle of strong El Niño and TNA events, which made its outcome completely different. 2024 exhibited the highest carbon emissions (0.81± 0.09 PgC y-1) in the 15-year record (Extended Data Tables 2 and 3, Extended Data Fig. 7), 605% higher than Group 1. It was also the hottest, driest, and strongly degraded by fire over the last two decades35. Annual temperatures were 1.2 ˚C higher than in Group 1, and annual precipitation was 462 mm lower than in Group 1. The burned area was 182% higher than in Group 1, producing the most extensive burned area observed since 2010 in Amazonia (Fig. 3a, Extended Data Tables 2 and 3)."

Line 159: "These findings underscore the dominant role of human activity in driving year-to-year changes in Amazonian carbon fluxes". The link between human activity and the El Nino impact needs to be explicit, for example as additional ignition sources? Fragmentation reducing resilience?

Authors' answer:
Thank you for the suggestion. With the strong association between drought and warming years and the substantial increase in burned area, it was necessary to include the study to identify the origin of fires: whether natural or anthropogenic. We included this study, which showed 98% of fires are anthropogenically induced.

--For the strong 2023 wet season sink and its potential interactions with La Nina, can you elaborate more on ecological mechanisms, e.g., access to groundwater (and associated literature). It looks like 2024 was not just due to a fire event, but the loss of the soil water buffer supporting physiological processes.

Authors' answer:
Thank you for the suggestion. Dear colleague, I think this could be another paper. To address the questions you are asking, it is necessary to integrate our top-down and bottom-up measurements to provide more physiological evidence for 2023 and 2024. We will also include COS results to bring the impact on photosynthesis.

Figure1: Include units on the figure.

Authors' answer:
This region of influence is calculated using the density of hourly locations at each back-trajectory in each grid cell. Then, they are the point densities in one grid cell, which each point corresponds to an hourly location from the back trajectories. For this reason, we do not have unity for the cells of the regions of influence.

Figures throughout are of poor resolution and blurry, with the y-axes on Fig 3 illegible.

Authors' answer:
Sorry about the resolution quality. The problem occurred during the process in the Nature system to produce the PDF files. We corrected these problems, and now we will submit the PDF rather than the doc file.

Figure 3: Are these colours colour-blind friendly?

Authors' answer:
Thank you again. We changed the colours in Figure 1 and the Extended Data Figures 2, considering colour-blind friendly police

Figure 4: Precipitation is presented, when it is fairly standard practice to use metrics like MCWD, as this provides a stronger indicator of water deficits experienced by vegetation, potential tree mortality, and as a potential metric of fuel dryness and fire risk.

Authors' answer:
Thank you for the suggestion. We adopted precipitation because, to understand carbon flux, it is important to know the exact amount for all months of the study. Monthly precipitation is used in the correlations and missforest procedures. MCWD is on an annual basis, and we calculated the weighted mean across the regions of influence.

The references provide appropriate credit to relevant work, but at times statements are made earlier in the text without reference (e.g., interpretation of the results), and a related statement is made later in the text with a series of appropriate references.

Authors' answer:
Thank you for the suggestion. In the current version, with many changes, we tried to better address the references.

Methods
--Could you provide more information how fire emissions were calculated. Emission factors vary substantially between agricultural areas, understory fires, and burning for deforestation. How was this variation addressed in your statistical approach and when quantifying uncertainty?
Authors' answer:
Thank you for the comments. Unfortunately, we were instructed to reduce the description of the published methodology (Gatti et al., 2021). We revised the text, and the resume was really incomplete. Now the description (still resumed) is "To calculate CO_2 from fire, we used the CO flux, subtracted from the natural CO emissions (eq. M2). After the step $F_{CO} – F_{CONatural}$, we used the CO/CO_2 ratio to calculate how much CO_2 came from the fire (all calculations were done in moles).

--The analysis uses deforestation and degradation data from PRODES and DETER. Estimating degradation for high biomass closed canopy forests is notoriously difficult for Amazonia, and it would be helpful to consider this uncertainty in estimates. Degradation is more than fire. Explicitly state whether the flux signal includes non-fire emission associated with degradation or are those included in the NBE or fire flux?

Authors' answer:
In the first version of the paper, we used 3 types of data correlated to burned area:
1 - Monthly data from DETER, whose motivation for use was the monthly result; however, the beginning of this data was in 2026. For this reason, we only used Supplementary Figures 3 and 4.
2 – The MODIS product also didn't show a nice correlation with fire emissions derived from CO measured in our study. We used this data only for the miss forest calculations, because it is available monthly.
3- The product that best correlated with the calculated fire emissions was the area burned by Mapbiomas, integrating all types of vegetation that burned, with annual temporal resolution. Then, in all annual analyses, the parameter used was burned area from Mapbiomas, including all types of burned vegetation (primary and secondary forest, pastures, agricultural areas and other vegetation). When comparing the fire emissions in 2024 with group 1 and with other years, the increase is exactly the same as the burned area from Mapbiomas. Fire spots, compared with fire carbon emission, didn't show similar variabilities.

--The approach for group uncertainties, where group sizes were different, appears appropriate.

Authors' answer: The propagation of uncertainty for the groups used the same equation we used for the time series mean: IUPAC recommendations - Quadratic mean for many years

$\bar{x}_q = \sqrt{\frac{\sum x_i^2}{n}}$, $\bar{x}_q =$ Quadratic mean; x_i is the uncertainty for each year, and n is the number of years.

Referee #3 (Remarks to the Author):

Please note, that we reference line numbering as in the word document, not the PDF.

1. Summary of the key results
This work presents an extensive data set of 15 years of consecutive measurements of the carbon balance of the Amazon derived from the CARBAM measurement campaign, where Vertical Profiles (VP) of atmospheric CO2 content where collected across sites in the Amazon region. The data indicates that the Amazon is a carbon source in the majority of years within the study period mainly caused by years of extreme drought, high deforestation rates and/or fire activities which correspond to years with the highest deltaVP (positive CO2 balance, i.e. CO2 release to the atmosphere).

Authors' answer: We would like to thank you for your suggestions.

2. Originality and significance: if not novel, please include reference

The data set presented is extensive and consists of long-term observations of the severe anthropogenic impacts in the Amazon, which is a region of (global) significance as its huge carbon reservoir is important to stabilize continental and global climate, apart from being an important region rich in biodiversity and biocultural diversity. The 15-year time span covers different climatic extremes that occurred in the region, but also drastic changes in deforestation rates (all-time high and low rates) and fire activity which are strongly influenced by institutional and jurisdictional governance in Brazil. The measured CO2 profiles, together with multiple available remote sensing data sets, covering different drivers of forest change, allow the authors to investigate the impacts of these changing drivers to estimate the effect on Amazonia's carbon balance (expressed as net biome productivity). To know if the entire Amazonia is a carbon source or sink as well as its trends is of high significance and interest to the wider readership. However, we have some doubts if the manuscript has originality in a sense that similar results have been published before. We believe that the evaluation of this data set in its entirety (15 years), including the recent period 2018-2024 is novel, while parts of the same data set (the CARBAM campaign) have been published before with similar reasoning and argumentation of the drivers (deforestation, fire, dry periods) (Gatti et al. 2021, 2023). Now we want to bring the fact to your attention that the Methods section of this manuscript is almost identical to the one published in Gatti et al. 2023. We understand that the authors in Gatti et al., 2023 focused on a different aspect in the interpretation of the data (enforcement of deforestation laws on the carbon balance) and the topical overlap with this study is due to the nature of the data and evaluation methods. However, we do take issue with the fact that large parts of the wording of the Methods section in this manuscript are a replication to those in Gatti et al., 2023. To demonstrate that entire sections of previous articles were not simply copy-pasted, we expect the authors to describe their established methods in their own words in a different way, referencing the original publication.

Authors' answer:
We thank the reviewer for your criticisms and suggestions.

Regarding originality.
In addition to the fact that the years from 2021 to 2024 are original, we also have a completely different approach to interpreting the results.
Considering the reviewers' suggestions to use statistical methods to define the groups of years, since in the first version of the paper we worked with 5 groups of years, the statistical analysis indicated 3 groups.
The 3 groups' definitions and analyses led to an important change in the paper.
First, we make clear, at the end of the introduction, the main objective of this paper:

"There are many questions about the main drivers of Amazonia's carbon emissions and variability. Studies based on modelling and bottom-up techniques show increased emissions during extreme climate (warm and dry) years, suggesting that extreme events are the primary driver of increased carbon emissions. However, our direct atmospheric measurements reveal a more complex situation."

"Here, we use our 15-year history of Amazonian carbon fluxes derived from atmospheric CO_2 measurements to answer important questions: What are the main factors driving increased carbon emissions and variability in the Amazon? Are they caused by global extreme climate events or direct human activities (deforestation, biomass burning and degradation) in the Amazon?"

This is an extremely important question. Since our study produced direct measurements of CO_2 in the atmosphere, we can address it using our time series of more than 1100 vertical profiles in the Amazon over 15 years.

Then, the originality is based not only on new, unpublished data, but also on the use of direct CO_2 measurements from the atmosphere, with spatial (regional scale) and temporal resolution. The use of the 15-year period, separating the years as one group of wet years (2011, 2012, 2013, 2014, 2017, 2018) with low human activities (such as deforestation and burned area), another group that essentially presented high human activities (mainly deforestation from 2019 to 2022), and another group characterised by extreme climatic events, such as El Nino and TNA, associated with increase of biomass burning. We also included a study verifying the lightning-induced fires. Since biomass burning consistently increased during drought years in Amazonia, it was important to clarify whether this increase was due to natural or anthropogenic causes. We found that only 2% is due to natural causes.
We believe all these improvements in the revised paper will resolve the originality issue.

3. Data & methodology: validity of approach, quality of data, quality of presentation
The authors describe how individual data sets were processed. It is concisely written and often sufficient information is provided. However, what is largely missing is a thorough statistical analysis of the drivers of carbon loss. At a first glance, the analysis is merely done by visual inspection. However, the analysis approach is hidden in one sentence in line 483, where "influence maps as spatial weighting functions for all studied parameters to determine how each parameter influenced the carbon flux" is mentioned, referencing Gatti et al. 2021. Unfortunately, tracing the methods description of this analysis step of that publication is not very detailed, so the reader needs to go back to this publication and still does not understand how the weighting process was done and what the statistical measures are. Additionally, since a large part of the results section of this manuscript is about time-series analysis of the entire study region, one would expect that additional (statistical) analysis is conducted to compare time series, including uncertainty measures, are provided – which is not the case.

Authors' answer:
We thank you for the suggestions. We believe that the use of statistical analysis led to a qualitative improvement in the paper.

About the weight mean analysis.
- The flux calculation does not consider the region of influence. It is based on the integration of the 12 height samples in the vertical profiles, which we consider the CO_2 (or CO) concentration measured at each height/flask, subtracted from the background concentration, then divided by the travel time of the air masses from the coast to the sampling site for each flask/height.

Since the flux calculation is more influenced by the surface near the local site, this influence decreases with distance. It is necessary to consider human activities and climatic conditions based on the distance from the local sample site. This is why we use regions of influence. For example, if biomass burning occurs 2000 km away from the sample site, the carbon flux measured is expected to be much lower than if it had occurred only 10 or 100 km away. The same influence happens with all other parameters. This is why it was necessary to use the weighted mean for the

not-measured parameters, such as precipitation, temperature, deforestation, burned area, fire spots, EVI, VPD, etc.

The Methodology section allows us to write 3000 words. Since every paper brings many updates to the methodology and adds more parameters and studies, it is unfortunately impossible to describe all methodologies. Based on the answer to the first submission with comments and requests, we were instructed not to repeat the published methodology but to cite the previous papers. In the current version, the published methodologies are still more limited. Sorry, we understand the extra work. Include because now we add statistical and lightning-induced fire studies. The main text, figures, methodology, results and discussion for both new studies add many additional materials and content to the paper.
As an example, see the changes in the Extended data and Supplementary information.
Before answering the reviews, we described all the changes this current version of the paper has, compared to the first submitted version.
In a simple description, the uncertainty analysis is based on the Monte Carlo error propagation technique, applied in the CO_2 flux calculation, which is well described in Gatti et al. al. 2021 paper. The flux is calculated from atmospheric CO2 measurements, and we treated the analytical uncertainty as the standard deviation of 20 monthly measurements taken since 2004. Here, we used the historical mean (0.03 ppm for CO2). The travel time uncertainty was calculated by comparing 3 different back-trajectory methods for all samples/heights over 1 year at the 4 sites.
We hope the current version has addressed many of the reviewers' criticisms and questions. The background uncertainty considers the measurement uncertainty from the NOAA laboratory (the concentrations for the global stations RPB, ASC, and CPT) and, again, the residual from the smooth curve calculated from NOAA weekly samples (to transform into daily information) and the travel-time trajectories. This is a simple description, but the processes are very complex and were published by Gatti et al. (2021). The equations are based on IUPAC recommendations for uncertainty calculations. After computing all specific uncertainties for all terms included in the Column Budget Technique, we ran 1000 iterations, using Monte Carlo Randomization error propagation.
The other parameters are not produced by us, but their uncertainties are also published in the old papers.

This is our main criticism where we regard the quality of presentation as being not sufficient for a high-ranking journal.

Authors' answer:
Sorry about the resolution quality on the first submitted version. The problem occurred during the process in the Nature system to produce the PDF files. We corrected these problems, and now we will submit the PDF rather than the doc file.

Further, the presentation quality of results can be substantially improved. Sentences and figure captions are often incomplete, the presentation of results in some figures can be improved, incl. aggregating data to improve readability of the legend. We also miss a development of a thorough storyline that is easy to follow for readers outside this field. The reader who might not be familiar with the HYSPLIT model and the concept of back trajectory density is left in the dark, as it is not further explained in the main text how the regions were defined from the station data

and how changes in weather conditions, incl. wind, influence the extent of the 3-dimensional packages (Fig. 1). Once the authors talk about Vertical Profile regions, when describing outcome of the next analysis step (next figure) groups are named according to their colour which is confusing for the reader.

Authors' answer:
We thank you for the suggestions. We believe the current version of the paper is easier to read and understand, including for non-specialists in this area.

The description of results changes from providing changes in absolute values to percentages in one sentence. We regard the presentation in percentages as problematic because the impact of small percent changes can be huge for one variable, but acceptable for another. Sometimes, the conclusion drawn from a set of results is unrelated to what the data actually show (e.g. L.154-156). This is especially evident in the section "Amazon precipitation and temperature changes (L114-135)": it seems that different time periods are compared and conclusions are drawn based on that. The section reads confusing in parts for which we provide examples here: The paragraph starting in L116 compares the updated dataset (1979-2024) to the previously published part (1979-2018).

Authors' answer:
We respectfully disagree with the reviewer's interpretation because the data used for the 1979 to 2018 and 1979 to 2024 periods were the same: ERA5 data. This information is present in the text. In the Gatti et al (2021) the data available was ERA Interin. There are substantial differences between ERA Interim and ERA5 for the temperature data.

It is not clear what the conclusions of this paragraph are. The authors first follow a clear logic of explaining impacts during the "new" period 2018-2024 (L 115-L124). Starting in L125 they write about regional analysis, but then discuss a multitude of different time intervals, which is confusing. For example, in L126-128, we cannot follow the logic how an increase in temperature during 1997-2018 is explained by deforestation between 2018-2024: "During 2018-2024, this region lost 37,930 km2 (2.4%) of primary forest due to deforestation. From 1997 to 2018, annual mean temperature rose by 0.93 ± 0.13˚C and by 1.90 ± 0.18˚C during the dry-season peak."
In L130 and following we do not understand the logic either: How is 1979-2024 a six-year period (the latter two sentences in the paragraph below), and how does the observation from 1979-2018 (first sentence) connect to this? "From 1997 to 2018, annual precipitation exhibited a non-significant downward trend of 208 ± 167 mm, while dry-season peak precipitation fell by 34% (67 ± 23 mm). From 1979 to 2024, annual precipitation decreased by 304 ± 150 mm (12%), and during dry-season peak declined by 37% (77 ± 23 mm). Over the same six-year period, SAN lost 15,023 km2 (2.3%) of primary forest. ". To us, these sentences are inconsistent and confusing.

Authors' answer:
We re-wrote part of the discussion to make it clearer for readers. Thank you for pointing out the difficulty in understanding the text.
We used StatPlus software (version 7.3.3.0, free) to calculate changes in long-term precipitation and temperature variability, presented in the Extended Data Table 2 (in the first version of the paper; in the current version, Extended Data Table 1). P>0.05 indicates a not significant (NS) result.

Example of analyses we did for annual precipitation for SAN from 1979 to 2024:

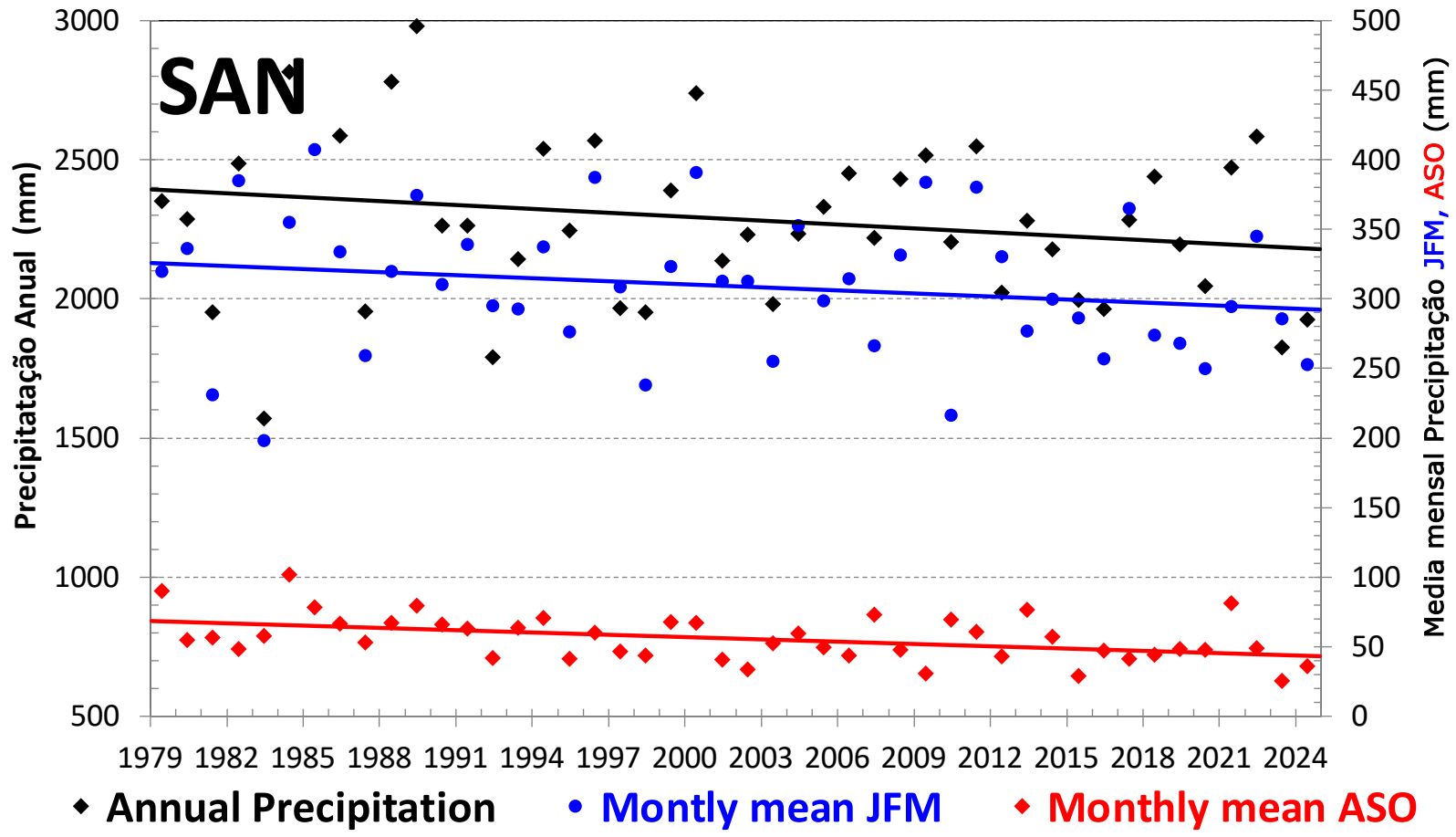

Below the statistical analysis

**SAN**								
**Acumulado Annual**								
**Annual Precipitacao Tendency**				p-value	Uncertanty			
R	0.29	R-quadrado		0.05				
	Coeficientes	Std Err						
Intercepto	2,976.74	337.43683						
data juliana	-0.01807	0.00895						
Annual total = 2,976.73834 - 0.01807 * dia Juliano								
2455				1/1/79	28856.00	-0.01807	303.6	
2152				1/1/25	45658.00	8.95E-03	**-150**	-3.3
**304**	**12%**	6.60						
**JFM**								
**JFM Precipitacao Tendency**				Valor-p				
**R**	0.287	R-quadrado		0.054				
	Coeficientes	Std Err						
Intercepto	1250.98451	161.32826						
data juliana	-0.01	0.00428						
soma JFM = 1,250.98451 - 0.00846 * dia Juliano								
1007				28856		0	142	
865				1/1/25		0.00428	-71.9	-1.6
142	0.14117606	3.09010696						
**AGO**								
**ASO Precipitacao Tendency**				Valor-p				
**R**	0.448	R-quadrado		0.00189				
	Coeficientes	Std Err						
Intercepto	339.67964	52.49074						
data juliana	0.00	0.00139						
soma ASO = 339.67964 - 0.00461 * dia Juliano								
207				28856		0	77	
129				1/1/25		0.00139	-23.4	-0.5
77	0.37481692	1.68385261						

StatPlus analyses

Linear Regression										
Dependent variable		Annual total Precipitation								
Independent variables		dia Juliano								
N		45								
Regression Statistics										
R		0.294	R-Squared		0.087	Adjusted R-Squared		0.065		
MSE		82928.616	S		287.973	MAPE		10.136		
Durbin-Watson (DW)		1.682	Log likelihood		-317.658					
Akaike inf. criterion (AIC)		14.207	AICc		14.209					
Schwarz criterion (BIC)		14.287	Hannan-Quinn criterion (HQC)		14.237					
PRESS		3940966.078	PRESS RMSE		295.934	Predicted R-Squared		-0.010		
Annual total Precipitation = 2,976.73834 - 0.01807 * dia Juliano										
ANOVA										
	d.f.	SS	MS	F	p-value					
Regression	1	337,947.14	337,947.14	4.07516	0.04978					
Residual	43	3,565,930.50	82,928.62							
Total	44	3,903,877.64								
	Coefficients	Std Err	LCL	UCL	t Stat	p-value	H0 (5%)	VIF	TOL	Beta
Intercept	2,976.74	337.43683	2,296.23	3,657.24	8.82162	3.36E-11	Rejected			
dia Juliano	-0.01807	0.00895	-0.03612	-0.00002	-2.0187	0.04978	Rejected	1	1	-0.29422
T (5%)	2.01669									
LCL - Lower limit of the 95% confidence interval										
UCL - Upper limit of the 95% confidence interval										

Regarding deforestation and degradation, different established data sets are used. However, for the inexperienced reader (who has not read the long history of Brazilian deforestation and degradation products), little explanation is provided on how they are related and where they differ (with, e.g., DETER deforestation data being the preliminary data set at monthly resolution of the annual consolidated PRODES deforestation estimation).

Authors' answer:
Thank you again. We also rewrote this part of the discussion to make it clearer for readers.

Regarding fire, it is not sufficiently explained how the fire carbon emission in form of CO2 is estimated using burned area, identifiable plumes in the VP during the dry season and fire activity data. But this is used in estimating the net biome carbon balance (L401 ff), whereas burned area is displayed in the time series (Fig. 4) and the connection to the other part of the analysis is unclear.

Authors' answer: Thank you again for pointing out the difficulty in understanding the concept clearly. Our results for fire carbon emissions are based solely on CO_2 and CO measurements we obtained (LaGEE/INPE) from the Vertical profiles. We provide further explanations at the beginning of the reply letter.
We explained in line 196 (previous version) and in the Methods section, lines 411-412. In the current version, this explanation is in lines 125-126, 192, and 409-410. NBE (net biome exchange) represents all non-fire carbon fluxes. We can separate the CO_2 from fire using CO. All other carbon emissions/absorptions from natural and anthropogenic non-fire sources remain included in what we named NBE.

It remains also unclear, why EVI data indicating vegetation activity are described in the Methods section but seem not to be used in the estimation of the carbon balance.

Authors' answer: We used the missing data imputation process (missforest) to fill gaps in the monthly carbon flux time series, and both EVI and VPD parameters were used for this purpose. Missforest description in the Methods section

Furthermore, it is unclear why only fire is considered in the calculation of the carbon balance and not carbon loss due to deforestation. We understand that this is difficult to separate from background data in comparison to fire, where it is possible to separate fire CO emissions from other environmental CO sources, but it needs to be clearly defined and explained. When the net biome productivity as a measure of the net carbon sink or source of Amazonia only considers fire in the carbon balance, how can conclusions be drawn with respect to deforestation and forest degradation?

Authors' answer: The carbon balance was calculated based on direct CO2 and CO measurements from the atmosphere, sampled in biweekly vertical profiles. We don't use indirect methods, such as the bottom-up technique. We use a top-down methodology based on direct measurements with regional resolution (4 vertical profile sites, with 2 measures per month). From the vertical profile samples, we calculated the flux. Total carbon flux, representing the total carbon balance (the resultant from all processes, natural and anthropogenic, emissions and absorptions) from both fire emissions and net biome exchange (TCF = FCF + NBE), as explained in the Methods section ('Carbon Fluxes Estimation'). The conclusions were based on the identified groups, in which emissions in Group 2 (2019, 2020, 2021, and 2022) were predominantly associated with deforestation. In contrast, in Group 3, emissions were mainly driven by fires, amplified by extreme climatic events.

On the use of climate data: it is unclear why VPD is used from CRU TS data set while air temperature data were taken from ERA5. Shouldn't both climate variables be consistent, i.e. used from the same data set? Furthermore, if precipitation data were used from GPCP because ERA5 data are biased this also has to be explained.

Authors' answer:
We used the missing data imputation process (missforest) to fill gaps in the monthly carbon flux time series, and both EVI and VPD parameters were used for this purpose. Missforest description in the Methods section
Precipitation data have been obtained from GPCP since 2021, when a specialist meteorological colleague told us it was a reliable product after extensive testing and validation. We also produced validation tests on the papers by Gatti et al 2021 and 2023. Since the data remain unchanged, we continue to use the GPCP in this paper.

The regional analysis is provided in the supplementary material; however, its results are not sufficiently presented in the main text.

Authors' answer: We agree, thank you for the comment, but the problem is the paper's size limit (2500 words). This is why the regional analyses are presented in Supplementary Discussion 3 and in many Figures. In some sections, we add a bit of discussion, as presented in the section on Annual precipitation and temperature changes (from line 92 to 99). In lines 197-198, we inform readers that the detailed discussion of the region is in Suppl Discussion 3, Extended Data Tables 1 and 2, Extended Data Figs. 2 and 3, and Supplementary Figs. 3, 4, and 5.

Figure captions:
Abbreviations are often not explained. Colour-coding of different years could be improved, years belonging to the same phase of deforestation-fire development could be aggregated in the legend to improve readability. Typing errors were copied over to figure captions, indicating not a very careful editing of the manuscript by the author team, see list below.
Fig 1 ED. The numbering a-h mentioned in the caption (also referred to in Fig. 1 main manuscript) is missing in the Extended Data pdf document (cut off?)

Authors' answer: In the revised manuscript, Extended Data Fig. 1 was renumbered as Extended Data Fig. 2, and panels a–h were included with increased detail.

Fig. 2 and 3 are blurry and not readable in both the submitted Word and the pdf document. We had to ask for a readable version from the Editor.
Caption typo Fig. 4 "Fig. 4 | Annual Mean Carbon Balance and the drives." - drivers
Caption typo Extended Data Fig. 6 | RBA and ALF annual mean Carbon balance and the drives. - drivers
Caption typo Extended Data Fig. 7 | TTC and SAN annual mean Carbon balance and the drives - drivers
Extended Data Fig. 8 | Seasonal RBA, TTC, ALF and SAN C Fluxes and Drivers. - fluxes and drivers OR always starting in capital letters in the entire manuscript.
Extended Data Fig. 8: What is the grey area indicating, the uncertainty as explained in the Methods section? This needs to be explained in the caption and /or included in the legend.

Authors' answer: Sorry about the resolution quality on the first submitted version. The problem occurred during the process in the Nature system to produce the PDF files. We corrected these problems, and now we will submit the PDF rather than the doc file. We reviewed and rewrote all figure captions (main text, Extended Data and Supplementary Material), including more details.

4. Appropriate use of statistics and treatment of uncertainties
No statistical methods are applied to the synthesis of time series data (see point 3. above). The determination of the uncertainties of the carbon fluxes is described in the Methods section, but not clearly indicated in any figure or table in the manuscript (but see our comment about ED Fig. 8). Uncertainty is provided for precipitation and temperature data, but not explained in the text.

Authors' answer: We would like to thank you for your suggestions, which led to a qualitative improvement in the paper and helped the scientific community and society better understand it. The main suggestion that led to a major change in the article was to use statistics to justify separating the groups by year. Using statistical methods, as detailed in this response and in the

article itself, we obtained 3 groups of years rather than the 5 groups in the first version of the article.
The statistical method used to determine the number of groups/years was K-means, applied in the PCA space. The classification of **Group 1** remained exactly the same, with the same years (2011, 2012, 2013, 2014, 2017 and 2018) and the same characteristics: group of rainy years and low human activity, characterised by deforestation and fires.
The current **Group 2** was classified as Group 3 in the previous version: the group characterised by a large increase in deforestation and low climate stress (2019, 2020, 2021, and 2022).
The current **Group 3** is the combination of groups 2 (2010, 2015, and 2016), 4 (2023), and 5 (2024), in the first version, characterised as years of extreme El Niño and TNA events. Interestingly, the division remained exactly as we had done previously; however, in the previous version, we separated the years 2023 and 2024 because they were very exceptional years (2023 showing the highest carbon absorption during the wet season, and 2024 the highest carbon emission, with the largest burned area, since the beginning of our study in 2010). However, with the statistical basis showing group separation, we believe the grouping of years is more scientifically grounded. Because, in the characteristics of Group 3, as determined by statistics, the years were characterised by El Niño and TNA climatic extremes, accompanied by a significant increase in biomass burning. It is now clear that increased biomass burning is also a key characteristic of this group. And so, it was necessary to add a study about lightning-induced fires. Was this increase natural or anthropogenic?

The uncertainty analysis is based on the Monte Carlo error propagation technique, applied in the CO_2 flux calculation, which is well described in Gatti et al. al. 2021 paper.
The flux is calculated from atmospheric CO_2 measurements, and we treated the analytical uncertainty as the standard deviation of 20 monthly measurements taken since 2004. Here, we used the historical mean (0.03 ppm for CO_2). The travel time uncertainty was calculated by comparing 3 different back-trajectory methods for all samples/heights over 1 year at the 4 sites.
We hope the current version has addressed many of the reviewers' criticisms and questions. The background uncertainty considers the measurement uncertainty from the NOAA laboratory (the concentrations for the global stations RPB, ASC, and CPT) and, again, the residual from the smooth curve calculated from NOAA weekly samples (to transform into daily information) and the travel-time trajectories. This is a simple description, but the processes are very complex and were published by Gatti et al. (2021). The equations are based on IUPAC recommendations for uncertainty calculations. After computing all specific uncertainties for all terms included in the Column Budget Technique, we ran 1000 iterations, using Monte Carlo Randomization error propagation.
The other parameters are not produced by us, but their uncertainties are also published in the old papers.

5. Conclusions: robustness, validity, reliability
Given the challenges and issues reported above, it remains open if the conclusions, although they are clearly written, are valid and reliable. This refers especially to the numbers mentioned (e.g. 8% reduction in precipitation, we could not find this number within the manuscript). The severe carbon loss in the Southeast region has not been mentioned explicitly before, yet the conclusion should not present new findings to the reader.

Authors answer:
We disagree with the reviewer's assessment. We conducted a significant amount of statistical work, presented in Extended data table 2 of the first version, and in this version, it appears as Extended data table 1. This is the second time we have performed this study. The first time was in the 2021 Nature paper (which the reviewer assumed was also used in this paper); however, there was no information indicating that we did so. We even shared our spreadsheets with the statistical results and the equation and coefficient data used in the analyses on pages 21, 22, and 23 of this cover letter.
The correlation between deforestation and rainfall loss is already well recognised in numerous other studies. At the time of our first publication, several articles by Argemiro Leite Filho already existed, including mathematical equations for predicting precipitation reduction (Fu et al., 2013; Costa et al., 2010; others), which were cited in the text (lines 28, 47, and others). More recent papers also show that deforestation is associated with reduced precipitation.

6. Suggested improvements: experiments, data for possible revision
Provide statistical analysis of how provided time series are related and how significant those relations are, especially with regard to fire and deforestation/degradation as a source of carbon loss. Revise calculation of carbon balance that includes carbon loss estimation of deforestation and forest degradation.

Authors answer:
The precipitation data is public available from GPCP and temperature is from ERA5.

7. References: appropriate credit to previous work?
Own previous work is referenced appropriately, with the exception of the Methods section. As we explain above, the authors copy-pasted entire paragraphs from their 2023 paper, which is not acceptable, even though the methods are similar.

Authors answer:
Since the authors are the same, the same person wrote, which makes it difficult for each paper to change words. But, since we are the authors, there is no plagiarism problem. Just resume issue.

8. Clarity and context: lucidity of abstract/summary, appropriateness of abstract, introduction and conclusions
Here, we highlight a selection of repetitive sentences and ask to adjust the text accordingly.
Abstract:
L25-26: remove duplicate of sentence “Forest loss reduces...” and also fix the references (different references cited in the duplicate): “Forest loss reduces precipitation, increases temperatures, and lengthens the dry season, driving higher carbon emissions4,5,8,10–12. Forest loss reduces precipitation, increases temperatures, and lengthens the dry season, driving higher carbon emissions13–16. “
Introduction:

Authors answer:
Thank you for the comment. The abstract was rewritten, and the repeated part was removed.

While the authors elaborate on climatic stress and the evolution of deforestation in the region, they do not explain a brief timeline regarding burned area during the time period of the investigation. As burned area is one of the key variables investigated related to deltaVP and a major source of CO_2, we suggest the authors add corresponding information in the introduction,

including the source of the burned area data (as PRODES is mentioned for deforestation alerts). At the end of the introduction, usually a short sentence or paragraph informs the reader what to expect in the following sections. Example from Gatti et al., 2023: “We compared the mean Amazonian carbon balance over 9 years (2010–2018)4 with the subsequent 2 years (2019 and 2020). (...)” . Such information is missing in the present manuscript.
We ask the authors to revise their manuscript in detail considering the following points, to improve readability, consistency and writing style. This applies especially to the Methods section, but also the other parts of the manuscript. The current version of the manuscript implies that the text has not been carefully revised among the team of authors before submission. Because of the many grammatical inaccuracies, missing words, differences in writing of abbreviations (small letters in one sentence, capital letters in another), punctation and so on, we expect a large task of manuscript editing in addition to the improvements on the analysis and presentation of results. Here we provide some examples for illustration:
– Consistent spelling of acronyms, e.g. PRODES or Prodes (both are used); BKG or bkg for background,
– Methods section: Some sentences appear unfinished or are not clear regarding their context. Example (L425-426): “To calculate the uncertainty for each vertical profile flux, from 1000 iterations using Monte Carlo randomization error propagation.” Example (L512-513): “The where the accuracy is 93.5%60.”
– use of dashes and hyphens: choose one form and stick with it. “–“ (e.g. L68) or “-“ (e.g. L69) or “—” (e.g. L92)
Parts we were out of scope of our expertise:
In the Methods section, L440 and following, the authors refer to a publication (Botia et al.) that criticized the authors’ approach in uncertainty treatment of gas concentration measurements. The authors respond to this in the present manuscript, but it is out of our scope to assess if this approach is valid, or if the correction suggested by Botia et al. should rather be applied.

Authors’ answer: We disagree with the treatment that supposedly has the objective to reduce uncertainty, but in fact brings more uncertainty, for many reasons. It was a modelling technique used to calculate water contents in the Amazon atmosphere. Many studies show that reproducing water vapour in the Amazon atmosphere is very challenging. The water was calculated for each of the more than 1100 vertical profiles sampled, and many others commented in the section and elsewhere. It was largely discussed with the authors of the Botia paper all the many reasons why we disagree with the treatment they did. In the end, we requested that our names be removed from the paper.

References
1. Gatti, L.V., C.L. Cunha, L. Marani, H.L.G. Cassol, C.G. Messias, E. Arai, A.S. Denning, L.S. Soler, C. Almeida, A. Setzer, L.G. Domingues, L.S. Basso, J.B. Miller, M. Gloor, C.S.C. Correia, others, Increased Amazon carbon emissions mainly from decline in law enforcement. Nature, 2023. doi: 10.1038/s41586-023-06390-0.
2. Gatti, L.V., L.S. Basso, J.B. Miller, M. Gloor, L. Gatti Domingues, H.L.G. Cassol, G. Tejada, L.E.O.C. Aragão, C. Nobre, W. Peters, L. Marani, E. Arai, A.H. Sanches, S.M. Corrêa, L. Anderson, others, Amazonia as a carbon source linked to deforestation and climate change. Nature, 2021. doi: 10.1038/s41586-021-03629-6.

Authors’ answer: We would like to thank you for your suggestions and comments. The current version was rewritten because the statistical results necessitated significant changes to the text,

figures, discussion, and conclusions. We believe the current version is easier to understand and satisfactory in terms of the form.

Referee #4 (Remarks to the Author):

I co-reviewed this manuscript with one of the reviewers who provided the listed reports.

Author comments: *Thank you for the suggestions and comments. We hope* the modified version is in accordance with the editor's and reviewer's suggestions and comments, and that the text is better and clearer. The detailed corrections are listed point by point in this response letter

We look forward to your response.

Thank you again for all the comments and suggestions that improved the manuscript.

Best Regards,

Luciana V. Gatti and the co-authors.

São José dos Campos, 24 August 2026

Response to Nature Editor and the Reviewers Dr Han Dolman, Reviewers 2, 3, and 4, about the submitted paper Nature manuscript 2025-11-29496

Thank you for all the suggestions, comments, and critical reviews that helped produce a better, clearer manuscript. We recognise that many suggestions are making the article stronger by providing clear arguments. Please see our answer and the description of all modifications we have made in the manuscript.

Referees' comments:

Referee #1 (Remarks to the Author):

Review of Gatti et al,

The authors have made a very thorough restructuring of the paper that handles all my previous comments very adequately, in particular the cluster analysis. The paper is now also better organised and easier to read. I must also complement the authors on Figure 4, which is a great summary.

Dear Han Dolman

Your suggestions in the first revision were very helpful! Many thanks. We also think that this version of the paper is easier to understand. We also learned more about how the Amazonian Forest functions in response to climate conditions, as well as the changes driven by the sequence of the three La Niña years.
Thank you again for your contribution.

I have a few minor remaining comments/suggestions:

L32 insert :an analysis” before 15-year.
Authors' answer: We included it

L46 through sink processes. I would suggest replace by carbon sequestration processes.
Authors' answer: We accepted and made the suggested change

L49 insert with before atmospheric and replace accounts in the next line by accounting.
Authors' answer: We accepted and made the suggested change

L81 it is not well explained why you choose 2018 as a break point. Did you use breakpoint regression analysis?

Authors' answer: Thank you for pointing out that it was unclear why we compared these two time periods, 1979-2018 and 1979-2024. We conducted this analysis in Gatti et al., 2021

(reference 1). Since the idea was to assess the influence of deforestation on precipitation and temperature, and given that we have lost a large amount of forest in the last six years (2018-2024), we decided to verify the effect of this recent loss on the overall scenario.

We changed the sentence to better explain this context.

It was: "To assess the influence of cumulative deforestation on the precipitation and temperature changes, we compared trends from 1979 to 2018[1] and from 1979 to 2024"

New sentence: "To assess the impact of the past six years, during which deforestation accelerated sharply between 2019 and 2022, on precipitation and temperature, we compared the previously studied period (1979–2018)[1] with the current period from 1979 to 2024, using the same database for both periods."

L156 how do you define moderate and strong etc.?

Authors' answer: Thank you again for pointing it out. We used the same ONI classification for TNA events. We added information on the criteria in this version and described them in the Methods.

It was: The years with TNA events were 2010 (moderate) and 2023/24 (moderate-strong), and the years with El Niño events were 2015/16 (strong-very strong) and 2023 (strong; see Fig. 3c).

The new sentence became: "The El Niño events occurred in 2015/16 (strong to very strong) and 2023 (strong; see Fig. 3c), while TNA warming events occurred in 2010 (moderate) and 2023/24 (moderate to strong). We applied the same ONI classification criterion28 to the TNA events (see Methods).

We added this information to the Methods, in the ONI section: To analyse the intensity of the events, we used the ONI classification anomaly28. For El Niño events: weak (0.5–0.9 °C), moderate (1.0–1.4 °C), strong (1.5–1.9 °C), and very strong (≥2.0 °C). We also add a new reference for this classification (ref 28).

28. CPC, C. P. C. I. Official NOAA CPC ENSO Strength Probabilities. *NOAA Climate Prediction Center* (2026).
https://cpc.ncep.noaa.gov/products/analysis_monitoring/enso/roni/strengths/

L209 it is interesting that the net balance is statistically not different between group 2 and 3. Maybe mention this? In fact you may consider writing a few lines on the fact that the NBE is always close to zero through the years (Fig 4), hence the difference is in the deforestation/fires.

Authors' answer: Thank you again for pointing it out. In fact, our conclusion was not clear about the main conclusion!

The new version now is (lines 218-226):
"…..essentially human-caused (0.49 ± 0.07 PgC yr-1). It is important to note that groups 2 and 3 do not differ significantly in carbon emissions, and both are dominated by deforestation and biomass burning, respectively. It is also noteworthy that the NBE across all three groups is close to neutral, indicating that direct human activities are the main risks to the Amazon. Drastic fire reduction is therefore essential to avert a tipping point, especially as escalating climate stress amplifies human impacts, and vice versa. Based on the consequences of the very strong El Niño 2015/16 in the southeast Amazon region, which has become an increasing source of carbon, similar consequences are expected after the strong El Niño and TNA 2023/24 for Amazonia. These results highlight the urgent need to implement a zero-deforestation policy, strengthen controls on biomass burning, and prioritise forest restoration programmes." We reduced part of the conclusion related to 2023 and 2024 to compensate for the increase in this comment.

Figure 2. It is interesting that the profile of group 3 extends well in the troposphere and appears structurally different from the other two. Maybe a small comment would help the reader. Is this transport by fire induced enhanced deep convection for instance?

Authors' answer: Thank you for the suggestion. We added a sentence in the Group 3 description (lines 160-162)
"We also observed in Fig. 2c that the Group 3 vertical profile showed an increase in the free-troposphere layer, unlike the other two; this is due to convective transport induced by the fire."

Fig 5 Extended data. You have the Portuguese spelling for the months (i.e. Fev instead of Feb). Please change.

Authors' answer: Thank you again for observing this mistake. We corrected. The Figure was Extended Data Fig. 6 (Extended Data 9 in submission)

Referee #2 (Remarks to the Author):

The authors have improved the revised manuscript. I appreciate their careful work to respond to questions about the previous version.

Overall, the authors have substantially addressed my original critiques. The additional info about spatial heterogeneity and policy vs climate was helpful. The additional deeper look into potential human vs lightning drivers of fire was also useful. More could have been stated about the distinction between deforestation fires (flaming with higher CO2 fraction) and degradation/understory fires (smoldering with higher CO fraction), although I am aware the authors are up against word limits. I agree with the authors that my comments about the strong 2023 wet season sink and its potential interactions with La Nina could be another extensive analysis and may not be possible to address within space limitations.

L153 stated that Group 3 comprises years characterised by "intense global climate

events, such as El Nino and TNA warming”. However, it looks like your Welch’s ANOVA for ONI and TNA were non-significant compared to other groups (Suppl. Table 1).

Authors’ answer: Thank you for this helpful comment. We agree that the description of Group 3 should more clearly distinguish the occurrence and intensity of the major climate anomalies from their differences among groups.

The revised text (lines 155-160): “Group 3 (Extended Data Table 3c, Extended Data Fig. 5) included years affected by major climate anomalies, including El Niño and TNA warming events. The mean ONI and TNA values for Group 3 were higher than those for Group 1 and Group 2. Any years from Groups 1 and 2 presented El Niño and/or warming TNA events. The El Niño events occurred in 2015/16 (strong to very strong) and 2023 (strong; see Fig. 3c), while TNA warming events occurred in 2010 (moderate) and 2023/24 (moderate to strong). We applied the same ONI classification criterion[28] to the TNA events (see Methods).

For the intro, it would help to have some background information on your atmospheric measurements to support the first mention in the text “However, our direct atmospheric measurements…”

Authors’ answer: Thank you for this observation. This issue really deserves a more detailed discussion in the text.

The new text is now in lines 169-173:
“…. which were 325% higher than Group 1. However, for this group, Net Biome Exchange (TCF – FCF = NBE) was -0.06 ± 0.10 PgC y-1 (nearly neutral-sink), indicating that the main source of carbon is not the forest itself, but, again, human influence that promotes fires. Furthermore, recent climatic conditions interfere with the consequences of an intense El Niño year on the forest, as can be observed in 2023 and 2024 (Extended Data Tables 2 and 3, Extended Data Fig. 7).”

The conclusions could be strengthened to clarify the message and provide a clearer, forward-looking statement based on the results.
Also, the second-to-last sentence referring to 2023/24 as a question is unclear.

Authors’ answer: Thank you again for pointing this out. We modified part of the conclusions to address your suggestions and those from Reviewer 1.
From lines 218-226:
“It is important to note that groups 2 and 3 do not differ significantly in carbon emissions, and both are dominated by deforestation and biomass burning, respectively. It is also noteworthy that the NBE across all three groups is close to neutral, indicating that direct human activities are the main risks to the Amazon. Drastic fire reduction is therefore essential to avert a tipping point, especially as escalating climate stress amplifies human impacts, and vice versa. Based on the consequences of the very strong El Niño 2015/16 in the southeast Amazon region, which has become an increasing source of carbon, similar consequences are expected after the strong El Niño and TNA

2023/24 for Amazonia. These results highlight the urgent need to implement a zero-deforestation policy, strengthen controls on biomass burning, and prioritise forest restoration programmes."

Referee #3 (Remarks to the Author):

A. Summary of the key results
The study represents an extensive data set covering 15 years of consecutive air column CO2 and CO measurements which were derived from the CARBAM measurement campaign for four sites in the Amazon biome. Each site integrates carbon fluxes from a region of a different size which altogether represent changes in the carbon balance of the Brazilian Amazon. The author team analysed the carbon balance as the result of changes in its anthropogenic (fire, deforestation, degradation, land-use) and climatological drivers for individual years in the 15-year time period. They conducted a statistical analysis to classify individual years into groups of wet/dry years in combination with high/low anthropogenic activities that include setting fires, deforest and degrade tropical rainforest.
B. Originality and significance: if not novel, please include reference
We appreciate the authors's response to our criticism regarding originality of the manuscript. We value the fact that by applying a statistical analysis that groups the individual years into 3 classes of wet/dry years, which can be characterized by ENSO events or Tropical North Atlantic Ocean influence, with low/high deforestation rates and fire occurrence. Especially, the additional work on lightning strikes and lightning-caused fires is novel and we highly value this additional analysis as it increases the originality of the manuscript. However, in some places the authors miss to take the final step, i.e. conclude the exercise with – in this example – a clear statement that the fire carbon emissions are human-caused and lightning-caused fires make up 5-10% of all fires.

Authors' answer: Thank you again for pointing this out. We modified parts of the conclusions to address your suggestions and those of Reviewers 1 and 2 as well. The study concluded that lightning-caused fires accounted for less than 2% of all fires in group 3 (1.6% in 2023 and 1.0% in 2024). We never cited that fires are caused by lightning 5- 10%.
Also, regarding the criticism that we "miss to take the final step", in version 2, lines 176-179, and in this version 3 of the revised manuscript, can be found at lines 183-187.

We also made changes to the conclusion text:
From lines 218-226: "It is important to note that groups 2 and 3 do not differ significantly in carbon emissions, and both are dominated by deforestation and biomass burning, respectively. It is also noteworthy that the NBE across all three groups is close to neutral, indicating that direct human activities are the main risks to the Amazon. Drastic fire reduction is therefore essential to avert a tipping point, especially as escalating climate stress amplifies human impacts, and vice versa. Based on the consequences of the very strong El Niño 2015/16 in the southeast Amazon region, which has become an increasing source of carbon, similar consequences are expected after the strong El Niño and TNA 2023/24 for Amazonia. These results highlight the urgent need to implement a zero-deforestation policy, strengthen controls on biomass burning, and prioritise forest restoration programmes."

This also applies to the analysis of the other drivers (deforestation, degradation, land-use for agriculture and pasture, temperature increase and precipitation decrease) as well. Their changes are presented (with improved quality), but the reasoning that they are indeed explaining the measured total carbon fluxes is left to visual inspection. An example on how to interpret TCF, FCF and NBE, when TCF is positive and NBE is negative is important, see comment below.
The manuscript text still uses similar framing of previous publications (Gatti et al. 2021 and 2023) which is acceptable, but the baseline text of those previous publications is not revised in such a way that it increases readability of the text to a wide audience, i.e. the average Nature reader.

Author's comments: We didn't use any part of the text to explain the findings from our previous paper, except in the Methods section. Our Gatti et al, 2021 paper is classified by Nature as "*This article is in the 99th percentile (ranked 45th) of the 476,377 tracked articles of a similar age in all journals and the 99th percentile (ranked 2nd) of the 927 tracked articles of a similar age in Nature*". We believe the readability was good, and the current citation count is 937.

Moreover, the analysis of deforestation, degradation and fire has been analysed for each individual region that the measurement sites cover in Tejada et al. (2023) and been compared against bottom-up carbon budgets, which analysed if individual years were either a source or sink of carbon.
Author's comments: The deforestation didn't come from the Tejada paper, but from a completely new study performed by the PRODES group. The fire numbers (not accounted for by Tejada) were performed by MapBiomas and BD Queimadas grups.

The study of a similar author team showed that large uncertainties can remain with respect to the identification of individual year's carbon balance and that the source-sink distribution of years and/or the size of the source (sink) can differ between the top-down (this study approach) and bottom-up (Tejada et al. 2023). We suggest, instead of adding more and more analysis and expanding the current manuscript with even more extended data and supplementary text, that the author team focusses on the grouping of the individual years and explains
- the implication of lighting- vs. human-caused fires as well as deforestation and degradation on the total carbon flux in those three groups of years, i.e. translating the area loss into influence of measured carbon fluxes.

Author's comments:
In this type of study, we begin with knowledge of the CO2 flux and balance. The objective of analysing many parameters and all available information is to understand the result for the whole region and/or the whole Amazon. To understand the immense variability observed, both from one region to another and from one year to another (not possible from bottom-up studies that always used the same tax for all years). Therefore, the aim is to learn, in an integrated way, how the various factors are affecting the forest. This is very different from the structure of bottom-up studies that start with areas lost due to deforestation and degraded by various types of activities and factors (fires, selective logging, border effect, mining, drought, etc.) and apply an average rate for all years for each of these factors, which may not be realistic because there are many

variations. In the case of Tejada et al., for example, they failed to calculate the emissions from fires, which is the largest source of carbon, and they also failed to test the trend of change in the factors for the decade in which our study was conducted (only the factor from the previous decade was used). All studies are valid and greatly expand our knowledge, but their development approaches differ significantly.

- the specific conditions that might have led to the carbon source in the 2023-24 El Niño event and the acceleration in droughts due to increased deforestation and degradation in the previous years.

Author's comments:
Our method, for example, showed us that the year 2023, despite having two extreme El Niño and La Niña events (starting in May), had the largest carbon sink observed in 15 years during the wet season. We were only able to learn the impact of La Niña events on the Amazon from our monthly and quarterly analyses, which would not have been possible using the bottom-up method and modelling. Many studies have reported that 2023 was a major source of carbon; however, our measurements showed that the sink during the 2023 rainy season offset 75% of the emissions from the dry season. We were also very surprised by this result. This is just one example of how direct measurement allows us to observe nature's reactions, and this is completely unprecedented and surprising.

by focussing on the translation of the changes in area to likely carbon fluxes (which is BTW explained better in the previous Gatti et al. publication, and can be referred to in this manuscript). The material is more or less there but needs the quantification and description from area-based changes to the influence on the TCF and NBE in a concisely written text that it understandably written for a wide readership. Of course, explanatory text that is required to understand applied methods and context in this manuscript has to stay but should clearly reference previous publications. It would help to avoid giving the impression that too many things are repeated and confuse the current originality of the study. We provide examples in the next section C.
C. Data & methodology: validity of approach, quality of data, quality of presentation
We thank the author team for their response and how they explained the applied statistical methods (site vs. region of reference). Again, referencing to the Tejada et al. 2023 study would also be helpful for the reader to understand how process (changes) influence the measurements taken at the sites. Furthermore, we suggest to focus the regional analysis on the two points suggested in the previous section.

Author's comments: The same comments from the authors are valid here**.**

We understand that 3000 words set a limitation to explain the applied methods in an understandable way, the current solution is not helping either. In previous publications Gatti et al. did explain why EVI, VPD, burned area, deforestation etc. were used, namely in the statistical analysis. The text blocks in the methods sections have remained, but the central sentence similar to Gatti et al. 2023 is missing. Specifically, the use of EVI and VPD are not explained, not in the Methods section of the main

paper, nor in the Supplementary Material section, even though it was mentioned in the response letter. We would ask the author team to add this important point. So, is it the same method to use all those variables in the imputation of the total carbon flux (TCF)? Please describe what exactly is meant by imputation.
VPD is used in the analysis, the title states that droughts are analysed, but VPD is not prominently described and discussed in the main text. When removing previously published text components, it should give space to discussing VPD changes in addition to precipitation and temperature.

Author's answer:
In the first revision, reviewer 3 commented, "*Now we want to bring the fact to your attention that the Methods section of this manuscript is almost identical to the one published in Gatti et al. 2023. We understand that the authors in Gatti et al., 2023 focused on a different aspect in the interpretation of the data (enforcement of deforestation laws on the carbon balance) and the topical overlap with this study is due to the nature of the data and evaluation methods. However, we do take issue with the fact that large parts of the wording of the Methods section in this manuscript are a replication to those in Gatti et al., 2023. To demonstrate that entire sections of previous articles were not simply copy-pasted, we expect the authors to describe their established methods in their own words in a different way, referencing the original publication."*

As you requested, we revised the Methods section to summarise the methodology published in other articles, but the Missing data imputation section (lines 465 to 476) was still 174 words, and EVI and VPD were still 91 words (lines 495 to 501). We are sorry for not providing a complete explanation, but all these methods were published in Gatti et al. (2021) and (2023).

We used MissForest to impute missing values in the monthly carbon flux time series, using both EVI and VPD as predictors. A detailed description of the MissForest procedure is provided in the Methods section. Although both variables were subsequently evaluated in the analyses, neither EVI nor VPD provided additional explanatory power for the variability in the carbon fluxes or improved model performance. Therefore, they were not included in the final models.

We think it is important to describe the historic land-use changes (section from lines 65-77), incl. increases in pasture, soybean and mining areas. However, this detailed information only makes sense to be reported if it explains how pastures, soybean fields and mining have influenced the measured carbon fluxes in the air column. If this cannot be differentiated, it could be a way to remove this text and describe the influence of deforestation and degradation on the 3 groups, incl. fire, and specifically the El Niño event.

Author's answer:
Since the study was published in Gatti 2021, we have learned how the historical deforestation and land-use change impact forest function and the ability to absorb carbon. This is why, after this time, we present this kind of analysis to help readers understand the level of stress in the Amazon due to human activities.

Quality of presentation. The quality of presentation has improved in many ways and we appreciate the detailed effort the author team put in this manuscript revision. We also acknowledge the effort put in to address detailed comments of every reviewer and still produce a consistent manuscript. Our suggestions for improvements aim at further increasing the manuscripts quality and ask the author team to implement the following:
1. Section Amazon climate changes:
a. The changes in precipitation and temperature for the two time periods compared in the text are not displayed in Extended Figure 1, the annual precipitation and dry-season precipitation should be plotted instead in two separate panels in this figure to substantiate the text from lines 82-87.

Author's answer:
Unfortunately, we used all the elements we could in the publication, considering the text, the method, the 10 extended data, and the 10 supplementary information.
In Extended Data Figure 1, we have 14 panels. It is impossible to add more elements to the figure and still have it show the details. We used the same scale to highlight that the most deforested areas show a large difference between minimum and maximum rainfall, and the same holds for temperature.
But the requested information is included in the text when it was considered important for understanding the region's situation, and in Extended Data Table 1.

b. Unclear why deforestation numbers are mentioned here again. Please clarify the difference from the above section on historic land-use change.

Author's answer:
Because deforestation interferes with precipitation and temperature. The main idea is not to inform about deforestation again, but to make the correlation between deforestation, reduced precipitation and increased temperature.

2. Caption of Fig. 3:
a. To allow readers outside your field to understand the meaning of the TCF, we suggest labelling (and mentioning in the manuscript text) what negative and positive TCF numbers mean, even though one can deduce it from the methods and equations.

Author's answer: We know that for the bottom-up community, the signals are opposite, but in the text, when we presented the NBE results, we added a sink or source to facilitate understanding (lines 33, 37, 39, 170, 171, 174).

b. Panel c: We would suggest to colour the axis and labels in the colour of the precipitation graph to help visual reading of the line colour.

Author's answer: Nature has very specific rules for figures, and axes cannot be coloured. Colour information can go in the legend.

3. We suggest to report changes in VPD and EVI to report drought conditions in the

individual years in addition to precipitation and temperature which display their changes, not absolute numbers, to indicate increasing drought conditions relative to average climate. We suggest to add this to Figure 3 and adapt respective descriptive text.
a. In the response letter, the author team seem to suggest that MCWD was used, but we could not find it in the text. We understand that it is also important to have a monthly metric for drought conditions. While the author team already uses VPD and EVI (among others) for gap filling, these variables could also be used to quantify drought conditions. In addition to precipitation the argument then still holds that deforestation and forest degradation starts to use precipitation. We suggest to clearly state this as an interpretation from the data rather than a proof arising from carbon fluxes shown here.

Authors' answer:
In the second submission, it seems the reviewers liked the changes to the Figures, which made them easier to understand. We also prefer to keep Figure 3c focused only on the parameters relevant to the paper's analysis and conclusion.
The main objective of the article was to separate direct human actions on the Amazon rainforest from climatic conditions influenced by global extreme events, to determine which factor(s) are primarily responsible for the changes in the Amazon.
In this case, we chose the parameters that could clarify the human impact would be deforestation and fires, which are directly produced by humans, and, in the case of climate change, we chose both the Oceanic Niño Index (ONI) and the Tropical North Atlantic Index (TNA), together with precipitation and temperature. We did not consider that either the EVI or the VPD could answer the proposed objective. Both are a consequence of climatic conditions, not the cause. Both EVI and VPD are correlated with other variables (such as temperature and precipitation) that are already being used in the analysis and that were determinant in the grouping of the years.

4. Figure 5:
a. Top line of data boxes displaying the bar plots of a number of drivers: The size of the bars displaying NBE, DEF and BA do not match.

Authors' answer: The Flux bars are on the same scale, but deforestation and burned area couldn't be on the same scale in the small centimetres of the figure space.

The NBE bar for group 2 is unreasonable large (value of 0.07) compared to group 3 (value of -0.06).

Authors' answer: The result is based on direct measurements of total concentration, and the fires and NBE are calculated using the site-specific ratios. Of course, the ratios could be separated by wet and dry years, and this will be possible in the future. We don't have them yet. But we have 4 ratios from direct measurements. This is more realistic than the bottom-up ratio, which uses a single value for all tropical forests.

For BA the bar in Group 3 should be roughly the double in size compared to BA in Group 1. Furthermore, the legend for these bars in the left-most box only mentions

carbon flux units and misses the area-based units. Please check and revise as this figure might be widely used.

Authors' answer: the same answer as the two answers above

b. Legend: Please check index for precipitation to have capital letters. Precipitation should be marked as annual precipitation, Temperature as mean average temperature, or use abbreviations and explain them in the legend text.

Authors' answer: We added clarification about Precipitation and Temperature in the legend.

5. The concluding general numbers for the Amazon region appear in the Conclusion section for the first time, we recommend to close Section "Grouping of years" with basin-wide numbers and reference corresponding data and figures (which do not include those number so far). Therefore, this section should describe and discuss Figure 5, which now appears in the conclusion section for the first time.

Authors' answer: Thank you for pointing out the absence of a Figure 5 citation. It is now cited in line 183.
Regarding the mean carbon balance over 15 years, it was ultimately omitted from the discussion because the aim of this paper was not the 15-year mean but to use the groups to address the main forest drivers (human activities x climate change). But the information is presented anyway in the Abstract and in the conclusion (2 times in lines 32-33 and 208-209). Our effort to reduce the number of words in the paper is always presented in the role paper.

6. Even though it has been published in Nature before, the terms used here Total carbon flux, fire carbon and net biome exchange (NBE) remains confusing for reader outside your field. It remains unclear, how from equation M1 (for which the definition of variables and their units needs to be added) these fluxes can be deducted from the background (lines 389-393 do explain one bit, the main text (lines 119-124 another) and seem not to match. Please explain the difference in carbon balance then TCF is positive and NBE is negative, or different in quantity – how should these different numbers be interpreted? To take one example from Figure 5, Group 3 has a TCF of 0.49 PgC p.a., because FCF is 0.55 PgC p.a. the remaining other carbon fluxes (uptake and release) lead to a negative NBE (-0.06 PgC p.a.). So, TCF means it is 0.49 PgC higher than the background carbon flux, which points to large carbon emissions. The calculation of the fire carbon flux, using equation M1, assigns a carbon flux of 0.55 PgC p.a. which exceeds TCF. What is the interpretation of the carbon budget for a negative NBE, does it mean that the ecosystem is reducing its carbon stocks? We think, explaining those examples clearly, helps to understand how the findings should be interpreted correctly.

Author's answer: We know that for the bottom-up community, the signals are opposite, but in the text, when we presented the NBE results, we added a sink or source to facilitate understanding (lines 33, 37, 39, 170, 171, 174).
About our Method, unfortunately we can't describe it again, but in the Methods, we presented enough to clarify these kinds of questions, as:

The background is for the measured concentrations, not for flux, but for one step in the flux calculation (there is a paper explaining the method carefully).

Further comments for this section are:
a. The description should focus on the different interpretations for the 3 groups and 2023-2024 years.

Author's answer: We agree.

b. Description of figures 2 and 3 in the text fall short of the content that both figures show, i.e. do not use the text to list the variables shown, rather the describe the content. When the individual years show quite some variability can you conclude a trend as indicated in the other sections?

Author's answer: The statistical method promotes the grouping of the years.

7. Description of Groups: Please check if all references to extended data figures and tables are necessary. The listed quantities refer to Extended Data Table 3 in all paragraphs. Which information is mentioned from the other display items and requires citations?

Author's answer: We checked all references in the legends

Detailed comments
1. Line 49: revise language to connect the text after semicolon to message of sentence.
Author's answer: Done
2. Line 59: add references to the statement on "extreme events as a reason for increased carbon emissions."
Author's answer:
There is a misunderstanding of what we stated in the paragraph from line 57 to 60, which includes 3 references.
The paragraph is below:
"During periods of extreme climatic variability, such as El Niño or anomalously high tropical North Atlantic sea-surface temperatures (TNA), these impacts are amplified through the interaction of accumulated forest loss, global warming, changing circulation, and large-scale anomalies[9,14,16]".
We discussed how cumulative forest loss amplifies El Niño and warming TNA, because forest loss itself causes drought and increases temperatures; global warming also increases the intensity and frequency of El Niño and warming in the tropical North Atlantic Ocean; circulation changes occur when these phenomena happen. We cited 3 studies that discuss these correlations in the Amazon.
3. Line 125 revise title of section to improve language and clarity
Author's answer: Unfortunately, there is a limit of 40 characters including space
4. Line 145: numbers are not shown in Table 3b
Author's answer: All numbers are there. The unique missing value is explained in the paper. The mean vertical profile for 2015 is missing because the VP was partial during the year. Then,

it was not correct to calculate the mean that does not reflect the year, but this is explained in the paper.

5. Line 145-146: it is the mean TCF and mean delta VP which values are shown, please revise.

Author's answer: Sorry, we didn't understand your suggestion.

6. Line 149-150: please mark qualitative meaning of ONI and TNA values in Figure.

Author's answer: This figure was simplified because more than one reviewer pointed out that the figure was complex in the first version. The Figure showed, in the first version, monthly means for Amazon carbon flux, ONI and TNA. In this version, we simplified by presenting quarterly means for Amazon carbon flux, ONI and TNA. Other reviewers liked the improvement. The qualitative ONI and TNA are already shown in Figure 3c (dashed lines) and explained in the legends (lines 324-326).

7. Line 151-152: statement not substantiated by display items, please add.

Author's answer: It is better explained in Group 2 (lines 145-154)

8. Line 154-155 Since ONI and TNA provide changes in temperatures I would suggest not to mention the difference in percentages.

Authors' answer: Thank you for this comment. We removed the difference in percentages.

9. Figure 3c could profit from marking El Niño years with a symbol in figure panel

Authors' answer: The dotted line is exactly for this purpose.

10. Line 159: add "(compare Extended Table 3a with 3b)" after Group 1.

Authors' answer: We cited the Extended Data Table 3 initially in the section and in other parts in this Grouping section. Because of the paper's size limit, we don't cite figures and tables every time.

11. Line 164, add reference to Extended Table 3c

Authors' answer: We cited the Extended Data Table 3 initially in the section and in other parts in this Grouping section. Because of the paper's size limit, we don't cite figures and tables every time.

12. Line 166: Mentioning Data Fig 7 is not needed since monthly changes are not mentioned in this sentence. Add "Mean" to "Fire carbon flux"

Authors' answer: Extended Data Figure 7 presents the seasonality within Group 3, which is important for readers. The name Fire Carbon Flux has been used since our published paper. Thanks for the suggestion.

13. Extended Figure 7: please mark wet-season months in figure panel or delineate them using stippled lines

Authors' answer: Extended Data Figure 7 presents the seasonality in 10 panels. We think adding more details will not be helpful for readers.

14. Line 194: do you mean people used fire to degrade the forest to then be able to deforest?

Authors' answer: yes.

15. Line 196: the section should close with a summary of biome-scale and time-average numbers. The reference to detailed subregional analysis should be moved where regional differences are discussed (and be included only when necessary for understanding the main story).

Authors' answer: the same answer as item 5.

16. Line 362: spell out abbreviations of sites when first mentioned.

Authors' answer: The name is exactly this. The global network has roles for Station names. And we adopted the criterion. The explanation is the coordination. If you think the name is related to the city, we don't measure the city.

D. Appropriate use of statistics and treatment of uncertainties
We thank the author team for their efforts to improve the use of statistics in this manuscript. We suggest to use the regional analysis only to explain regionally different impacts on 2023 and 2024 El Niño drought year.
As mentioned under point A, uncertainties in estimating the years' carbon balance (source or sink year) and the size of the source or sink depending on the top-down (this study) or bottom-up (Tejada et al. 2023) to be included in the discussion of this study. Readers would possibly interpret the findings of this study as a given...
Authors' answer: The same was pointed out twice above, and we answered.

E. Conclusions: robustness, validity, reliability
The Conclusion section has very much improved. It now substantiates better the findings of this manuscript. With the suggested updates in the sections A and B, we think the Conclusions are robust.
There are a few things we recommend to make it consistent and valid:
The Statement in Line 200 is fine if there is a biome-wide summary in the corresponding sections above.
Line 204-205: this information was not provided, please mention in text on climatic influence to be able to refer to these changes in conclusion
Line 206: to which time period do the temperature changes refer?
Line 215-216: you should be able to provide an answer to this question on how the 2023-24 El Niño event influenced TCF and NBE.
F. Suggested improvements: experiments, data for possible revision
Our concerns raised in the first review were addressed.
Authors' answer: Thank you for your suggestions. We adopted all suggestions allowed by the Nature roles.

G. References: appropriate credit to previous work?
Yes, can be improved wrt to previous findings of author team. Citing Tejada et al. to refer to the regional pattern the site measurements capture important information and can help to make the new measurements and knowledge provided with this study update more concise.
Authors' answer: Thank you for your suggestions. In this type of study (Top-down), we begin with knowledge of the CO2 flux and balance. The objective of analysing multiple parameters and all available information is to understand the result for the entire region and/or the entire Amazon. To understand the immense variability observed, both from one region to another and from one year to another (not possible from bottom-up studies that always used the same tax for all years). Therefore, the aim is to learn, in an integrated way, how the various factors are affecting the forest. This is very different from the structure of bottom-up studies that start with areas lost due to deforestation and degraded by various types of activities and factors (fires, selective logging, border effect, mining, drought, etc.) and apply an average rate for all years for each of these factors, which may not be realistic because there are many variations. In the case of

Tejada et al., for example, they failed to calculate the emissions from fires, which is the largest source of carbon, and they also failed to test the trend of change in the factors for the decade in which our study was conducted (only the factor from the previous decade was used). All studies are valid and greatly expand our knowledge, but their approaches to development differ significantly.

H. Clarity and context: lucidity of abstract/summary, appropriateness of abstract, introduction and conclusions
Our concerns regarding Abstract, introduction and Conclusion were addressed appropriately.

Authors' answer: Thank you for your very detailed paper revision.

References

1. Gatti, L.V., C.L. Cunha, L. Marani, H.L.G. Cassol, C.G. Messias, E. Arai, A.S. Denning, L.S. Soler, C. Almeida, A. Setzer, L.G. Domingues, L.S. Basso, J.B. Miller, M. Gloor, C.S.C. Correia, others, Increased Amazon carbon emissions mainly from decline in law enforcement. Nature, 2023. doi: 10.1038/s41586-023-06390-0.
2. Gatti, L.V., L.S. Basso, J.B. Miller, M. Gloor, L. Gatti Domingues, H.L.G. Cassol, G. Tejada, L.E.O.C. Aragão, C. Nobre, W. Peters, L. Marani, E. Arai, A.H. Sanches, S.M. Corrêa, L. Anderson, others, Amazonia as a carbon source linked to deforestation and climate change. Nature, 2021. doi: 10.1038/s41586-021-03629-6.
3. Tejada G, Gatti LV, Basso LS, Cassol HLG, Silva-Junior CHL, Mataveli G, Marani L, Arai E, Gloor M, Miller JB, Cunha CL, Domingues LG, Ipia A, Correia CSC, Crispim SP, Neves RAL and Von Randow C (2023) CO2 emissions in the Amazon: are bottom-up estimates from land use and cover datasets consistent with top-down estimates based on atmospheric measurements? Front. For. Glob. Change 6:1107580. doi: 10.3389/ffgc.2023.1107580

Referee #4 (Remarks to the Author):

I co-reviewed this manuscript with one of the reviewers who provided the listed reports.

Author comments: Thank you for the suggestions and comments. We hope the modified version is in accordance with the editor's and reviewers' suggestions and comments, and that the text is better and clearer. The detailed corrections are listed point by point in this response letter

We look forward to your response.

Thank you again for all the comments and suggestions that improved the manuscript.

Best Regards,

Luciana V. Gatti and the co-authors.
